# Synthesis and SAR Studies of Acyl-Thiourea Platinum(II) Complexes Yield Analogs with Dual-Stage Antiplasmodium Activity

**DOI:** 10.1021/acsmedchemlett.4c00545

**Published:** 2025-02-17

**Authors:** Fatima-Zahra Ishmail, Dina Coertzen, Sizwe Tshabalala, Meta Leshabane, Shante da Rocha, Mathew Njoroge, Liezl Gibhard, Lyn-Marie Birkholtz, John G. Woodland, Timothy J. Egan, Kathryn J. Wicht, Kelly Chibale

**Affiliations:** † Department of Chemistry, University of Cape Town, Rondebosch 7701, South Africa; ‡ Department of Biochemistry, Genetics and Microbiology, Institute for Sustainable Malaria Control, University of Pretoria, Hatfield 0028, South Africa; § Institute for Sustainable Malaria Control, School of Public Health and Health Systems, University of Pretoria, Hatfield 0028, South Africa; ∥ Department of Biochemistry, Stellenbosch University, Stellenbosch, Matieland 7602, South Africa; ⊥ Holistic Drug Discovery and Development (H3D) Centre, University of Cape Town, Rondebosch 7701, South Africa; # South African Medical Research Council Drug Discovery and Development Research Unit, Institute of Infectious Disease and Molecular Medicine, University of Cape Town, Observatory 7925, South Africa

**Keywords:** Platinum(II) complexes, antiplasmodium, malaria, gametocyte, acyl-thiourea, bipyridine

## Abstract

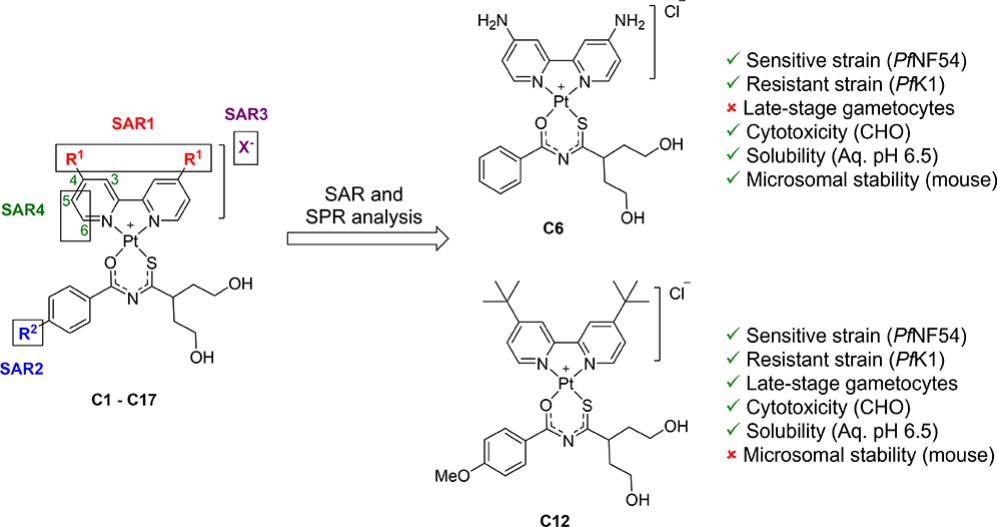

Mixed-ligand platinum(II) complexes incorporating bipyridine and acyl-thiourea ligands were synthesized and evaluated for their *in vitro* growth inhibitory activity against the human malaria parasite *Plasmodium falciparum* (*Pf*). The substituents at four distinct sites were varied to identify structure–activity relationships for this series. Most complexes displayed potent *Pf*NF54 activity with IC_50_ values in the nanomolar range and favorable cytotoxicity profiles. Five complexes (**C1**, **C11**, **C12**, **C15**, and **C17**) exhibited activity against both the asexual blood and sexual (gametocyte) stage parasites, with another complex (**C8**) exhibiting activity against late-stage gametocytes only. In addition, the complexes showed comparable ABS potency against the *Pf*K1 multidrug-resistant strain. The pharmacokinetic parameters of one analog (**C6**), which displayed good solubility and mouse microsomal metabolic stability, were measured. This work demonstrates the potential of acyl-thiourea platinum(II) complexes as selective, multistage-active antiplasmodium compounds as part of the search for new antimalarial agents.

The continuous emergence of drug-resistant strains of the most virulent human malaria parasite, *Plasmodium falciparum* (*Pf*), calls for the urgent development of new antimalarial chemotherapeutics.^1,2^ Within this context, it is vital to develop drugs that are active against resistant strains and across multiple stages of the parasite life cycle, particularly the asexual blood stage (ABS, symptomatic stage) and gametocyte (transmission stage) parasites.^3^ This is especially important since patients treated with drugs that target parasites in the ABS, or even those who are asymptomatic carriers, contain sufficient gametocytes to transmit the parasite from its human host to the mosquito vector, contributing to the spread of the disease.^4^ To accelerate drug discovery efforts, chemotypes successfully used in other disease areas can be exploited and optimized as novel compounds against malaria. In this regard, thioureas have gained recognition for their potential medicinal application. In the context of malaria, Verlinden et al.^5^ reported a series of alkylated (bis)urea and (bis)thiourea polyamine analogs exhibiting nanomolar *in vitro* antiplasmodium activity against both chloroquine-resistant (CQR) strains (*Pf*W2 and *Pf*HB3) and a chloroquine-sensitive (CQS) strain (*Pf*3D7). Additionally, Pingaew et al.^6^ reported a (bis)thiourea compound that exhibited antiplasmodium activity against the CQR *Pf*K1 strain with an IC_50_ value of 1.92 μM.

A less-explored approach in malaria drug discovery is the use of metal-containing drugs. Metallodrugs can be used to detect and treat several diseases and have important biological applications in cellular systems.^7^ Metal centers may also be added to organic drugs to improve their efficacy,^8^ as is seen with ferroquine (FQ), a ferrocenyl derivative of the 4-aminoquinoline drug chloroquine (CQ). In a study conducted by Dive and Biot, FQ showed a 1.7-fold increase in activity compared to CQ against the *Pf*3D7 strain and a 25-fold increase in activity against *Pf*K1.^9^ FQ acts via the same mechanism of action as CQ (inhibition of hemozoin formation) and causes oxidative stress due to the ferrocenyl moiety.^10^ FQ is currently in phase IIb clinical trials in combination with artefenomel.^11^ Additionally, FQ is also in phase II clinical trials in combination with ZY-19489, a triaminopyrimidine identified from a high-throughput screen against ABS *Pf* parasites.^12^

The success of FQ highlights the potential of using transition metals in the search for antimalarials with novel mechanisms of action. Metal complexes that have been studied for their antiplasmodium activities are mainly based on the 4-aminoquinoline scaffold of CQ. Sanchez-Delgado et al. were one of the first groups to synthesize transition metal (Rh and Ru) complexes with a CQ moiety that exhibited comparable antiplasmodium activity to CQ.^13^ Although CQ derivatization has dominated the metals-in-malaria field, one of the advantages of complexation chemistry is the relative ease of varying the ligand. Because of their stability toward air and moisture, 2,2′-bipyridine (bpy) ligands and derivatives thereof have been among the most frequently used ligands in complexation chemistry.^14^ The bpy ligands have also been used in several applications ranging from supramolecular and macromolecular chemistry to electrochemistry and photochemistry.^15^ Transition metal complexes using bpy as a ligand have been reported to show anticancer activity,^16^ antimicrobial activity,^17^ and antiplasmodium activity.^18^

Previous studies from our group showed that mixed-ligand platinum(II) complexes exhibited appreciable antiplasmodium activity.^19^ The current study expands on the previously used bpy and acyl-thiourea scaffolds and further explores the varying substituents on these moieties. A series of platinum(II) complexes were synthesized for evaluation against the CQS *Pf*NF54 and CQR *Pf*K1 strains. In this study, we analyze structure–activity relationships (SARs) for this series and, for the first time, evaluate the activity of the platinum(II) complexes against both ABS and sexual stages of *Pf* parasites as well as their cytotoxicity and the pharmacokinetic properties of a front-runner.

The synthetic procedures for complexes **C1**–**C17** and their precursors follow modifications of methods reported in the literature (Scheme 1).^19,20^ Compounds **1a** and **1b** were synthesized via the condensation of [2,2′-bipyridine]-4,4′-dicarboxylic acid in dimethylformamide (DMF) or methanol (MeOH) to form the methyl amide and ester, respectively. For the acyl-thiourea ligands **2a**–**2d**, the respective benzoyl isothiocyanates were reacted with diethanolamine under argon.

**Scheme 1:**
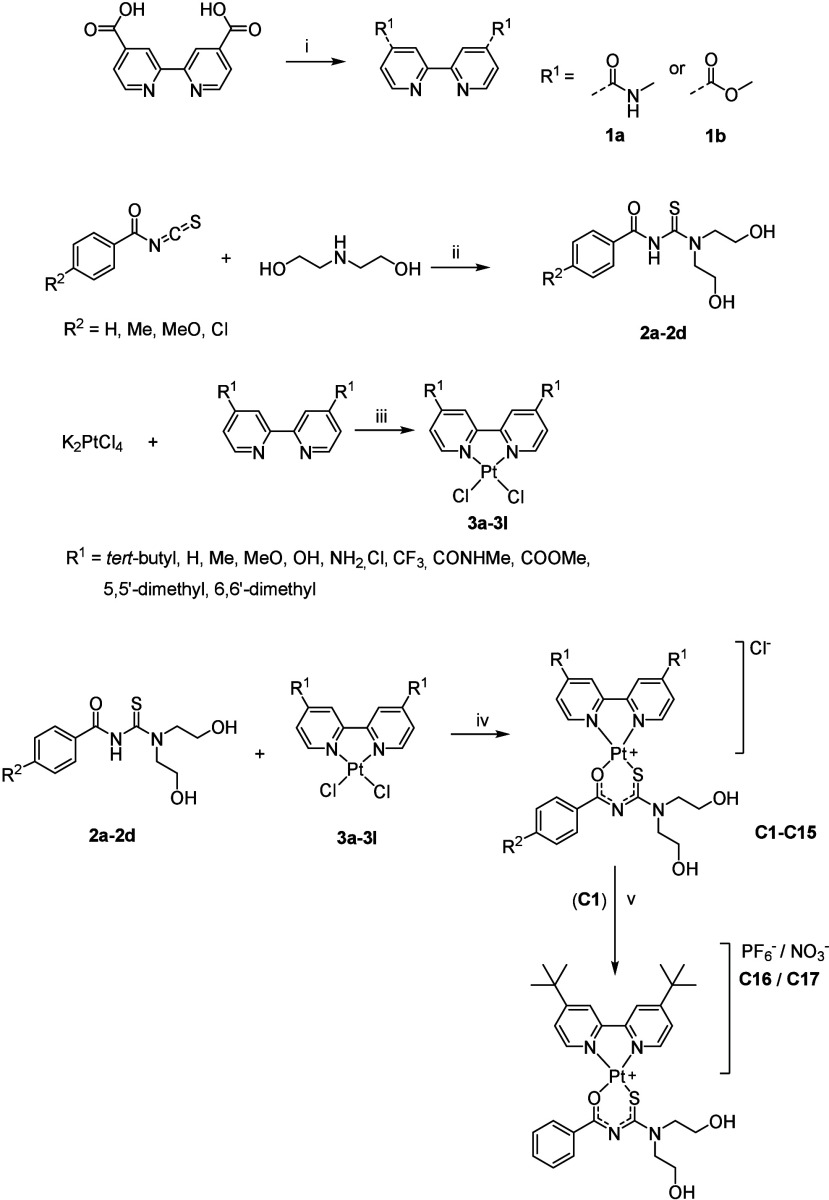
Synthesis of Mixed-Ligand Platinum(II) Complexes and Their Precursors Reagents and conditions: (i) Et_3_N, DMF, HATU, 2 h, 27 °C/Et_3_N, MeOH, HATU, 24 h, 50 °C; (ii) anhydrous DCM, 0 °C, argon, 4–20 h; (iii) H_2_O, 4 M HCl/H_2_O, 95 °C, 2–24 h; (iv) Et_3_N, acetone, DMF or MeCN, 60 °C/80 °C, 4–24 h; (v) NH_4_PF_6_, anhydrous DCM/EtOH (1:1), argon, 1 h/AgNO_3_, anhydrous MeOH, argon, 3 h.

The [PtCl_2_(diimine)] precursor complexes were synthesized according to the method described by Morgan and Burstall,^21^ wherein the relevant bpy was added to a solution of potassium tetrachloroplatinate in either acidified or neutral water at 95 °C. The bpy–acyl-thiourea ([Pt(diimine)(L-O,S)]^+^) complexes were synthesized via the dropwise addition of the relevant acyl-thiourea and triethylamine (Et_3_N) in DMF, acetone, or acetonitrile (MeCN) to a solution of the respective [PtCl_2_(diimine)] complex in either DMF, acetone, or MeCN. To determine the influence of the Cl^–^ counterion, salt metathesis was used for the counterion exchanges between the Cl^–^-containing *tert*-butyl analog (**C1**) and NH_4_PF_6_ or AgNO_3_ (Scheme 1). The complexes were characterized via ^1^H and ^13^C NMR spectroscopy (Figures S1–S37). The *in vitro* antiplasmodium activities of the synthesized compounds against the ABS parasites and early- and late-stage gametocytes were evaluated using previously reported methods^22−24^ (Table 1). The antimalarial compounds CQ, methylene blue (MB), and MMV390048 (a clinically validated *Plasmodium* phosphatidylinositol 4-kinase inhibitor^25^) served as positive controls for these assays. For the combined metal complexes, four sites for SAR analysis (SAR1–SAR4) were explored (Figure 1).

**Figure 1:**
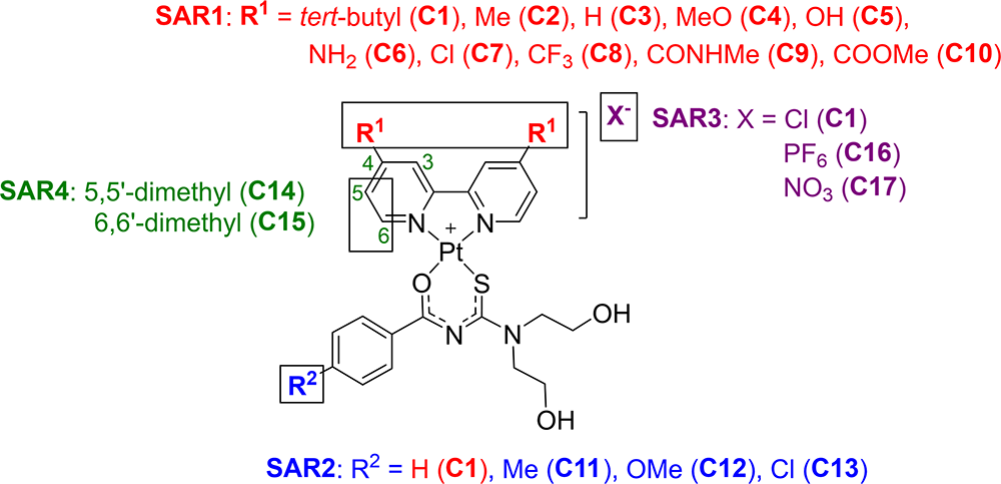
Mixed-ligand platinum(II) complexes showing the four SAR sites and their respective substituents.

**Table 1:**
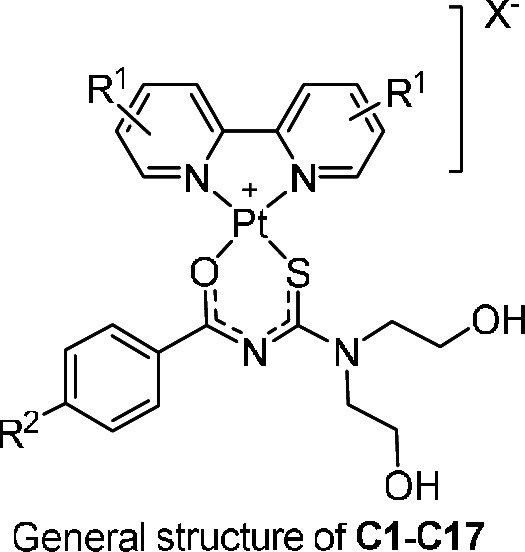
Biological and Pharmacological Data including IC_50_ Values for Whole-Cell Potency against *Pf*NF54 and *Pf*K1 ABS Parasites,^a^ Early (II/III) and Late (IV/V) Gametocyte Stage Parasites,^b^ and Cytotoxicity against the CHO Cell Line, as well as an Evaluation of Solubility and Microsomal Metabolic Stability

										Microsomal metabolic stability (% remaining after 30 min)	
Compound	R^1^	X^−^	R^2^	*Pf*NF54 IC_50_ (nM)	*Pf*K1 IC_50_ (nM)	RI	EG/LG IC_50_ (nM)	CHO IC_50_ (μM)	Aqueous solubility (μM) pH 6.5	Mouse	Human
2*				>5000	ND	NA	ND	>50	ND	ND	ND
**K**_**2**_**PtCl**_4_*				>5000	ND	NA	ND	>50	ND	ND	ND
3a*				>5000	ND	NA	ND	>50	ND	ND	ND
**C1**	*tert-*butyl	Cl	H	151 ± 17	433 ± 37	2.9	6362 / 3539 ± 1639	17	95	37	24
**C2**	Me	Cl	H	1415 ± 276	541 ± 44	0.4	>20 000/>20 000	>50	170	72	70
**C3**	H	Cl	H	142 ± 12	184 ± 22	1.3	>20 000/>20 000	>50	140	75	72
**C4**	MeO	Cl	H	266 ± 29	164 ± 7	0.6	>20 000/>20 000	>50	195	88	63
**C5**	OH	Cl	H	>5000	ND	NA	>20 000/>20 000	>50	ND	ND	ND
**C6**	NH_2_	Cl	H	36.3 ± 0.6	102 ± 13	2.8	ND/>20 000	>50	100	94	43
**C7**	Cl	Cl	H	>5000	ND	NA	>20 000/>20 000	>50	ND	ND	ND
**C8**	CF_3_	Cl	H	>5000	ND	NA	>20 000/380 ± 198	>50	ND	46	47
**C9**	CONHMe	Cl	H	>5000	ND	NA	>20 000/>20 000	>50	ND	ND	ND
**C10**	COOMe	Cl	H	>5000	ND	NA	>20 000/>20 000	>50	<5	ND	ND
**C11**	*tert-*butyl	Cl	Me	114 ± 8	29 ± 10	0.3	3030/216 ± 56	30	30	37	41
**C12**	*tert-*butyl	Cl	OMe	224 ± 13	13 ± 10	0.1	4610/405 ± 75	25	25	45	51
**C13**	*tert-*butyl	Cl	Cl	52 ± 4	252 ± 83	4.9	>20 000/>20 000	23	23	47	33
**C14**	5,5-dimethyl	Cl	H	241 ± 40	886 ± 59	3.7	ND	>50	>50	ND	ND
**C15**	6,6-dimethyl	Cl	H	2908	2764	0.9	9509/1413 ± 35	5	5	ND	ND
**C16**	*tert-*butyl	PF_6_	H	124 ± 37	252 ± 94	2.0	>20 000/8922	11	11	ND	ND
**C17**	*tert-*butyl	NO_3_	H	14 ± 8	135 ± 41	9.6	3184/3647	16	16	ND′	ND
**CQ**				11 ± 2	143 ± 3	12.7	ND	ND	ND	ND	ND
**MB**				NA	NA	NA	190/900	ND	ND	ND	ND
**MMV390048**				NA	NA	NA	215/134	ND	<5	>95	>95

a SYBR Green I-based proliferative assay over 96 h. Precursor compounds with an asterisk were tested by using a 72 h pLDH assay.

b Luciferase reporter assay over 48 h. Shown are mean IC_50_ ± S.E. values from three independent biological repeats (*N* = 3), each performed in technical triplicates (*n* = 3). Absence of S.E. indicates *N* = 1. RI is the resistance index (RI = *Pf*K1 IC_50_/*Pf*NF54 IC_50_). Cytotoxicity IC_50_ was determined using the activity of the mixed-ligand platinum(II) against the mammalian CHO cell line (*N*, *n* = 1, 3). The solubility and microsomal metabolic stability of selected compounds against humans and mice are also reported. Abbreviations: CQ, chloroquine; MB, methylene blue; *Pf*, *Plasmodium falciparum*; EG, early-stage gametocytes; LG, late-stage gametocytes; ND, not determined; NA, not applicable.

In SAR1 (complexes **C1**–**C10**), the substituent at the 4,4′-positions of the bpy was varied while the substituent on the acyl-thiourea moiety remained fixed as phenyl (R^2^ = H). Complexes **C1**–**C4** and **C6** displayed good to moderate antiplasmodium activity against the *Pf*NF54 and *Pf*K1 strains, with **C6** being the most potent against *Pf*NF54 (IC_50_ = 36.3 nM). These findings suggest that potency against *Pf* is positively influenced by the electron-donating ability of the substituent in the 4,4-positions (**C1**–**C4** and **C6**) of the bpy ligand, with *tert*-butyl (**C1**), hydrogen (**C3**), and the primary amine (**C6**) substituents resulting in the highest potencies. On the other hand, complexes containing an electron-withdrawing substituent, such as chloro (**C7**), trifluoromethyl (**C8**), methyl amide (**C9**), and methyl ester (**C10**) on the bpy did not show appreciable ABS whole-cell potency (IC_50_ > 5 μM).

For SAR2 (**C11**–**C13**), the substituent on the bpy was kept constant (*tert*-butyl), and the *para* position of the phenyl ring of the acyl-thiourea ligand was substituted with a methyl, methoxy, or chloro group. The addition of a methyl (**C11**) or methoxy (**C12**) functionality at the R^2^ position resulted in complexes that displayed equipotent activity against *Pf*NF54 compared to the parent complex (**C1**). However, with a chloro substituent in this position (**C13**), the whole-cell potency improved 3-fold against *Pf*NF54 compared to **C1**. This contrasts with what was observed for SAR1, in which analogs with an electron-withdrawing substituent were less potent against the ABS. Furthermore, complexes **C11** and **C12** were better tolerated in *Pf*K1 with resistance indices (RIs) of <1, demonstrating that they are not cross-resistant with CQ.

For SAR3, salt metathesis was performed to exchange the Cl^–^ counterion with a hexafluorophosphate (PF_6_^–^) or nitrate (NO_3_^–^) ion. While the PF_6_^–^ analog (**C16**) showed comparable activity to the Cl^–^ derivative (**C1**), interestingly the NO_3_^–^ analog (**C17**) showed an 11-fold increase in activity, which could potentially arise from increased nitric oxide (NO) produced from NO_3_^–^. NO_3_^–^ is an oxidant of NO^26^ and can be produced via the nitrate–nitrite–NO synthase pathway in mammalian cells.^27^ NO protects against severe malaria, highlighting its therapeutic potential.^28^ However, to the best of our knowledge, there are no reports on the effects of counterion exchanges from Cl^–^ to NO_3_^–^ in compounds tested *in vitro* for antiplasmodium activity.

Lastly, the pharmacological effect of substitution with a methyl group at different positions on the bpy ring was determined (SAR4). Changing from the 4,4′-positions (**C2**) to the 5,5′-positions (**C14**) improved the *Pf*NF54 activity by 5-fold, whereas changing to the 6,6′-positions (**C15**) decreased the activity by 2-fold. It is noteworthy that the metal salt (K_2_Cl_4_Pt) and precursor compounds **2a** and **3a** did not inhibit *Pf* proliferation at a maximum concentration of 5 μM against *Pf*NF54, highlighting the structural importance of combining the two moieties to observe appreciable antiplasmodium activity.

All the complexes were tested for their early- and late-stage gametocyte activity in a dual-point assay (1 μM and 5 μM). Complexes with inhibition of viability of >50% at 1 μM and >70% at 5 μM were further tested in full dose–response assays to determine their IC_50_ values (Table S2). Most of the compounds containing the bulky hydrophobic *tert*-butyl (**C1**, **C11**, **C12**, and **C17**) or trifluoromethyl (**C8**) substituent on the bpy rings exhibited gametocidal activity (IC_50_ < 5 μM). Additionally, complex **C15** with a 6,6-dimethyl substituent on the bpy was active against both early-stage gametocytes (EGs) and late-stage gametocytes (LGs), with a moderate potency of 1413 ± 35 nM against the LGs. Complexes **C11** (R^2^ = Me) and **C12** (R^2^ = MeO) showed a ≥1.4-fold improvement in activity against EGs compared to **C1**. Additionally, complexes **C11** and **C12** had a >10-fold increase in LG activity compared to EG activity, with their LG IC_50_ values below 1 μM. Complex **C8** did not exhibit ABS activity but showed potent activity against LGs with an IC_50_ of 380 ± 198 nM, an uncommon phenotype for compounds active against gametocytes. Therefore, within this series of compounds, three have potent ABS activity with moderate gametocidal activity (**C1**, **C16**, and **C17**), two have potent dual-stage activity (**C11** and **C12**), and one is selective toward late-stage gametocytes (**C8**). A difference in the potencies of the complexes against the ABS parasites compared to gametocytes is often observed for antimalarials and can arise due to several factors.^29,30^ These factors include differences in the stage-specific expression of the target of the compound and its capacity to diffuse across the cell membrane of the parasite in a particular life cycle stage.^31^ These results are promising for the potential future development of new dual-stage-acting antimalarials capable of disease treatment and blocking the transmission of mature gametocytes from the human host to the mosquito vector.

Additionally, the cytotoxicity of the metal complexes against the Chinese hamster ovary (CHO) cell line was determined (Table 1). These data indicate that all the complexes with submicromolar ABS activity are selective for *Pf* (selectivity indices > 100) and have a negligible effect on the mammalian CHO cells tested.

An HPLC-based method was used to determine the kinetic solubility of the selected synthesized metal complexes. All the compounds tested from SAR1 exhibited good solubility with values ranging between 95 and 195 μM (Table 1). The complexes belonging to SAR2 were poorly soluble, except for **C13**, which recorded a solubility of 150 μM. In a series of organic compounds, a decrease in lipophilicity generally increases solubility. However, in the case of these metal complexes, the solubility of the compound increased with higher lipophilicity of the bpy substituent (based on the Craig plot). This deviation from the trend seen in organic compounds could potentially be linked to the dissociation capacity of the anion (Cl^–^) and the cationic species, which likely varies between the different analogs. For this series, compounds containing the bulkier *tert*-butyl substituent were less soluble than those with the smaller substituents (Me, H, OH, and NH_2_), but no trend was seen within these smaller moieties. Complex **C4**, containing the water-solubilizing MeO substituent at the 4,4′-positions on the bpy, was the most soluble.

A subset of complexes with an aqueous solubility above 10 μM were profiled for microsomal metabolic stability. A range of metabolic stabilities were observed for the tested complexes, with <50% being considered poor and >85% being considered good (Table 1). The microsomal stability of the most active complex against *Pf*NF54, **C6**, was determined to be 94% in the presence of mouse liver microsomes (MLM) but only 43% in the presence of human liver microsomes (HLM), highlighting the species differences in the metabolism of this complex. Unsurprisingly, the analogs containing the *tert*-butyl substituent were moderately to poorly stable in the presence of both MLM and HLM, presumably via oxidative metabolism, as previously reported.^32^

Given the suitable microsomal metabolic stability profile of **C6** (MLM, 94%), good aqueous solubility (100 μM), and potent whole-cell activity (36.3 nM), an *in vivo* pharmacokinetic (PK) experiment was conducted with this analog. In this regard, three replicates of female Balb/C mice were dosed at either 3 mg/kg (intravenously, i.v.) or 10 mg/kg (orally, p.o.). Blood was sampled at specific time intervals over 48 h. The corresponding PK parameters were calculated using noncompartmental analysis (Table 2). PK analysis showed that **C6** exhibited a high steady-state volume of distribution (V_ssu_ = 4560 mL/kg (i.v.)) and high clearance (CL_u_ > 1000 mL min^–1^ kg^–1^ (p.o.)) of the unbound fraction, contributing to the low bioavailability (3.8% (p.o.)) which results in a lower amount of the test compound in systemic circulation.

**Table 2:**
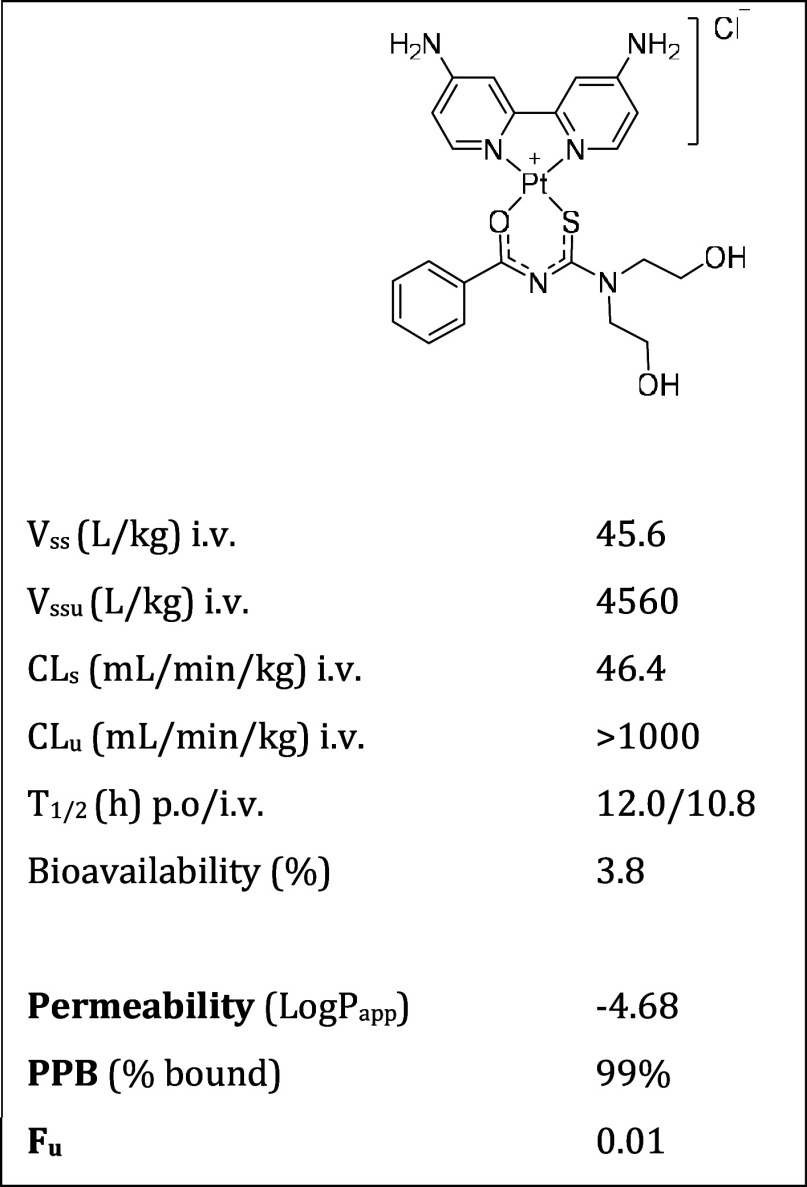
Pharmacokinetic, Permeability, and Protein Plasma Binding Parameters of Complex **C6**^a^

a Abbreviations: V_ss_, steady-state volume of distribution; V_ssu_, steady-state volume of distribution of unbound fraction (V_ssu_ = V_ss_/F_u_); CL_s_, clearance; CL_u_, clearance of unbound fraction (Cl_u_ = Cl_s_/F_u_); *T*_1/2_, half-life; P_app_, apparent permeability; PPB, plasma protein bound; F_u_: fraction unbound (F_u_ = (100 – PPB)/100).

It is known that Fe^2+^/Fe^3+^ redox chemistry has an important role in the bioactivity of ferrocene derivatives.^33^ The production of reactive oxygen species (ROS) is one of the modes of action of ferroquine and other ferrocene derivatives^34^ and is linked to the correlation between their bioactivity and electrochemical behavior. We determined whether a similar correlation could be made for the mixed-ligand platinum(II) complexes reported herein given that metal centers have the potential to be oxidized and reduced. The electrochemistry of the complexes was studied using cyclic voltammetry (CV), which uses a triangular potential waveform. The CV experiments were performed in either dry MeOH or DMF containing tetrabutylammonium chloride (TBACl) as the supporting electrolyte. The working electrode was glassy carbon with a platinum wire as the counter electrode and Ag/AgCl as the reference. The anodic (*E*_pa_) and cathodic (*E*_pc_) peak potentials at 0.1 V/s and half-wave potentials (*E*_1/2_) were measured (Table 3). All of the tested complexes undergo reversible one-electron Pt^2+/^Pt^+^ reductions (Figures S38–S46). Complexes **C2**, **C4**, and **C8** undergo a second nonreversible reduction (*E*_pc2_), which is most likely ligand-based and linked to reductions of the substituent on the bpy. Looking at SAR2, the more electron-withdrawing the substituent, the lower the *E*_1/2_ (V vs Fe/Fe^+^) value was. This trend was expected, as a more electron-withdrawing substituent reduces the electron density around the metal center, making it easier for the metal to be reduced from Pt^2+^ to Pt^+^. The same trend is seen for most of the complexes in SAR1, as the *E*_1/2_ increases in going from the more electron-withdrawing substituent to the less electron-withdrawing substituent (**C8** < **C3** < **C2** < **C1**). However, unlike ferrocenyl compounds, these complexes do not undergo oxidation, suggesting that the production of ROS is unlikely to be one of the mechanisms of action of these complexes. This is also the case for other bpy-containing platinum(II) complexes that only undergo reductions.^35,36^

**Table 3:** Electrochemical Data for Mixed-Ligand Platinum(II) Complexes^a^

Compound	*E*_pa1_ (V)	*E*_pc1_ (V)	*E*_pc2_ (V vs Ag/Ag^+^)	*E*_1/2_ (V vs Ag/Ag^+^)	*E*_1/2_ (V vs Fe/Fe^+^)
**C1**	–1.233	–1.127		–1.252	–1.670
**C2**	–1.074	–1.016	–1.084	–1.045	–1.585
**C3**	–1.210	–1.014		–1.131	–1.530
**C4**	–1.136	–1.064	–1.515	–1.100	–1.640
**C6**	–1.138	–1.068		–1.103	–1.643
**C8**	–0.710	–0.607	–1.272	–1.183	–1.076
**C11**	–1.239	–1.128		–1.190	–1.601
**C12**	–1.247	–1.133		–1.190	–1.608
**C13**	–1.233	–1.119		–1.176	–1.594

a *E*_1/2_ was corrected to ferrocene for comparison. Abbreviations: *E*_pc_, cathodic peak potential; *E*_pa_, anodic peak potential; *E*_1/2_, half-wave potential.

Furthermore, no correlation between the half-wave potentials and whole-cell potency of the complexes was observed, and their electrochemical properties are unlikely to contribute to the differences in their pharmacological response.

Although the data suggest that these complexes may not generate ROS via their own redox chemistry, they could potentially disrupt the redox equilibrium within the cell via an alternative mechanism. Platinum complexes are well-known for their capacity to bind to sulfur-containing molecules and proteins, including glutathione (GSH), although this often leads to reduced drug potency.^37^ Conversely, GSH can also act as an activator to reduce the oxidation state of metal complexes, subsequently forming a more active species.^38^ This is likely to occur via oxidation of GSH to glutathione disulfide (GSSG), which can simultaneously also produce ROS.^38^ In the ABS of *P. falciparum*, GSH is an important antioxidant that removes toxic free radicals, often produced during the mitochondrial electron transport chain or heme detoxification processes.^39^ Thus, GSH contributes to maintaining the redox equilibrium within the parasite. We therefore evaluated the capacity of selected complexes (**C1** and **C6**) to bind to GSH using a UV–vis absorption titration protocol as previously described.^40^ Despite an increasing GSH concentration, the UV–vis spectra show a minimal decrease in absorbance (Figures S47 and S48), indicative of weak binding. Thus, these GSH binding studies show that the tested platinum analogs only bind weakly to GSH with log *K* values of 2.45 ± 0.05 and 2.58 ± 0.19 for **C1** and **C6**, respectively (Table S1). Consequently, it is unlikely that these platinum complexes will elicit their biological efficacy via disruption of the GSH-mediated equilibrium of ROS within the mitochondrion.

Additionally, platinum complexes such as oxaliplatin and cisplatin can cause hemolysis and in turn hemolytic anemia.^41,42^ Therefore, to evaluate the *in vitro* hemolytic activity of a subset of the platinum(II) complexes reported herein (**C1**, **C6**, and **C17**), a hemolysis assay was conducted in a 96-well plate with a starting drug concentration of 10 μM. Triton X-100, a known hemolytic agent, and CQ, which does not induce hemolysis *in vitro*, were used as the controls. Triton X-100 causes a dose-dependent increase in the percentage of lysed red blood cells (RBCs) over a 72 h period (Figure S49). Contrastingly, complexes **C1**, **C6**, and **C17** do not induce hemolysis when tested up to 10 μM (Figure S49). Furthermore, the hemolytic activity of these compounds remained relatively constant when RBCs were incubated for both 1 and 72 h. Therefore, these complexes are unlikely to elicit their biological activity by causing morphological damage to RBCs.

In summary, a series of mixed-ligand platinum(II) complexes, most of which are novel compounds (**C4**–**C17**), were synthesized. Four SAR sites were explored, and it was found that, for SAR1, ABS activity was highly dependent on the presence of an electron-donating group at the 4,4′-positions of the bpy ligand. For SAR2, substituting hydrogen with chloride improved the potency 3-fold compared to the parent compound **C1**. Generally, the *Pf*K1 strain was more sensitive to these SAR2 analogs than the *Pf*NF54 strain. For SAR3, the counterion exchange of Cl^–^ with NO_3_^–^ improved the activity 11-fold, highlighting the importance of exploring this SAR in metal complexes. Changing the methyl substituent from the 4,4′-positions (**C2**) to the 5,5′-positions (**C14**) on the bpy improved the *Pf*NF54 activity by 6-fold, whereas changing it to the 6,6′-positions (**C15**) decreased the activity by 2-fold. This suggests that having a substituent at the 6,6′-positions on the bpy may result in steric hindrance. Furthermore, six analogs showed inhibition of late-stage gametocyte viability at <5 μM. Unlike FQ, these complexes do not undergo oxidation and are therefore unlikely to act via oxidative stress. Additionally, no correlation was observed between the half-wave potentials of the tested complexes and their whole-cell potencies, suggesting that the activity is not significantly affected by the redox properties of the different analogs. This was further supported by GSH UV–vis titrations, which showed negligible binding of the tested complexes to GSH. Furthermore, these complexes do not induce *in vitro* hemolysis over a 72 h incubation period. The aqueous solubility of all tested complexes was good (≥95 μM), and the microsomal metabolic stability ranged from 24–94% remaining after 30 min of incubation in the presence of both mouse and human liver microsomes. Most of these reported compounds exhibited no CQ cross-resistance, with five complexes showing greater activity in the *Pf*K1 strain, underscoring the importance of considering metal complexes in the fight to overcome antimalarial drug resistance. The ABS growth inhibition data along with the gametocyte activity suggest that the SAR2 complexes in particular can be further optimized as potent dual-stage antiplasmodium agents.

## Safety

No unexpected safety hazards were encountered during this work. For the synthesis of the platinum complexes, the standard precautions were taken, and protective clothing was worn when working with toxic and corrosive chemicals such as triethylamine and hydrochloric acid. The solvents used in this study, dimethylformamide, methanol, ethanol, dichloromethane, acetone, and acetonitrile, are flammable and hazardous. They should be kept away from heat, sparks, open flames, and other sources of ignition. All biological studies were performed under approved biosafety protocols at the BSL-2 level.

## Supporting Information Available

Full experimental details, compound characterization data, cyclic voltammograms, glutathione binding and hemolysis studies (PDF)

## Abbreviations

^1^H and ^13^C NMR: proton and carbon nuclear magnetic resonance
ABS: asexual blood stage
bpy: 2,2′-bipyridine
CHO: Chinese hamster ovary
Cl_u_: clearance
CQ: chloroquine
CQR: chloroquine-resistant
CV: cyclic voltammetry
DMF: dimethylformamide
Et_3_N: triethylamine
FQ: ferroquine
F_u_: fraction unbound
GSH: glutathione
GSSG: glutathione disulfide
HLM: human liver microsomes
i.v.: intravenously
MB: methylene blue
MeCN: acetonitrile
MeOH: methanol
MLM: mouse liver microsomes
p.o.: orally
P_app_: apparent permeability
*Pf*: *Plasmodium falciparum*
PK: pharmacokinetic
pLDH: plasmodium lactate dehydrogenase
PPB: plasma protein bound
RI: resistance index
ROS: reactive oxygen species
SAR: structure–activity relationship
*T*_1/2_: half-life
V_ss_: steady-state volume of distribution

## References

[ref1] Blasco B.; Leroy D.; Fidock D. A. Antimalarial Drug Resistance: Linking *Plasmodium falciparum* Parasite Biology to the Clinic. Nat. Med. 2017, 23 (8), 917–928. 10.1038/nm.4381. 28777791 PMC5747363

[ref2] Shibeshi M. A.; Kifle Z. D.; Atnafie S. A. Antimalarial Drug Resistance and Novel Targets for Antimalarial Drug Discovery. Infect. Drug Resist. 2020, 13, 4047–4060. 10.2147/IDR.S279433. 33204122 PMC7666977

[ref3] Birkholtz L.-M.; Alano P.; Leroy D. Transmission-Blocking Drugs for Malaria Elimination. Trends Parasitol. 2022, 38 (5), 390–403. 10.1016/j.pt.2022.01.011. 35190283

[ref4] Abebaw A.; Aschale Y.; Kebede T.; Hailu A. The Prevalence of Symptomatic and Asymptomatic Malaria and Its Associated Factors in Debre Elias District Communities, Northwest Ethiopia. Malar. J. 2022, 21 (1), 167 10.1186/s12936-022-04194-7. 35659661 PMC9166605

[ref5] Verlinden B. K.; Niemand J.; Snyman J.; Sharma S. K.; Beattie R. J.; Woster P. M.; Birkholtz L. M. Discovery of Novel Alkylated (Bis)Urea and (Bis)Thiourea Polyamine Analogues with Potent Antimalarial Activities. J. Med. Chem. 2011, 54 (19), 6624–6633. 10.1021/jm200463z. 21882831 PMC3191323

[ref6] Pingaew R.; Sinthupoom N.; Mandi P.; Prachayasittikul V.; Cherdtrakulkiat R.; Prachayasittikul S.; Ruchirawat S.; Prachayasittikul V. Synthesis, Biological Evaluation and in Silico Study of Bis-Thiourea Derivatives as Anticancer, Antimalarial and Antimicrobial Agents. Med. Chem. Res. 2017, 26 (12), 3136–3148. 10.1007/s00044-017-2008-5.

[ref7] Kaluđerović G. N.; Gómez-Ruiz S.; Maksimović-Ivanić D.; Paschke R.; Mijatović S. Metals in Medicine. Bioinorg. Chem. Appl. 2012, 2012, 705907 10.1155/2012/705907. 22956920 PMC3432323

[ref8] Biot C.; Castro W.; Botté C. Y.; Navarro M. The Therapeutic Potential of Metal-Based Antimalarial Agents: Implications for the Mechanism of Action. Dalton Trans. 2012, 41 (21), 6335–6349. 10.1039/c2dt12247b. 22362072

[ref9] Dive D.; Biot C. Ferrocene Conjugates of Chloroquine and Other Antimalarials: The Development of Ferroquine, a New Antimalarial. ChemMedChem 2008, 3 (3), 383–391. 10.1002/cmdc.200700127. 17806092 PMC7162372

[ref10] Chopin N.; Bosson J.; Iikawa S.; Picot S.; Bienvenu A. L.; Lavoignat A.; Bonnot G.; Riou M.; Beaugé C.; Guillory V.; Biot C.; Pilet G.; Chessé M.; Davioud-Charvet E.; Elhabiri M.; Bouillon J. P.; Médebielle M. Evaluation of Ferrocenyl-Containing γ-Hydroxy-γ-Lactam-Derived Tetramates as Potential Antiplasmodials. Eur. J. Med. Chem. 2022, 243, 114735 10.1016/j.ejmech.2022.114735. 36122550

[ref11] Adoke Y.; Zoleko-Manego R.; Ouoba S.; Tiono A. B.; Kaguthi G.; Bonzela J. E.; Duong T. T.; Nahum A.; Bouyou-Akotet M.; Ogutu B.; Ouedraogo A.; Macintyre F.; Jessel A.; Laurijssens B.; Cherkaoui-Rbati M. H.; Cantalloube C.; Marrast A. C.; Bejuit R.; White D.; Wells T. N. C.; Wartha F.; Leroy D.; Kibuuka A.; Mombo-Ngoma G.; Ouattara D.; Mugenya I.; Phuc B. Q.; Bohissou F.; Mawili-Mboumba D. P.; Olewe F.; Soulama I.; Tinto H.; Ramharter M.; Nahum D.; Zohou H.; Nzwili I.; Ongecha J. M.; Thompson R.; Kiwalabye J.; Diarra A.; Coulibaly A. S.; Bougouma E. C.; Kargougou D. G.; Tegneri M.; Castin Vuillerme C.; Djeriou E.; Ansary A. F. A Randomized, Double-Blind, Phase 2b Study to Investigate the Efficacy, Safety, Tolerability and Pharmacokinetics of a Single-Dose Regimen of Ferroquine with Artefenomel in Adults and Children with Uncomplicated *Plasmodium falciparum* Malaria. Malar. J. 2021, 20 (1), 222 10.1186/s12936-021-03749-4. 34011358 PMC8135182

[ref12] Ramachandran S.; Hameed P. S.; Srivastava A.; Shanbhag G.; Morayya S.; Rautela N.; Awasthy D.; Kavanagh S.; Bharath S.; Reddy J.; Panduga V.; Prabhakar K. R.; Saralaya R.; Nanduri R.; Raichurkar A.; Menasinakai S.; Achar V.; Jiménez-Díaz M. B.; Martínez M. S.; Angulo-Barturen I.; Ferrer S.; Sanz L. M.; Gamo F. J.; Duffy S.; Avery V. M.; Waterson D.; Lee M. C. S.; Coburn-Flynn O.; Fidock D. A.; Iyer P. S.; Narayanan S.; Hosagrahara V.; Sambandamurthy V. K. *N*-Aryl-2-aminobenzimidazoles: Novel, Efficacious, Antimalarial Lead Compounds. J. Med. Chem. 2014, 57 (15), 6642–6652. 10.1021/jm500715u. 25007124

[ref13] Sánchez-Delgado R. A.; Navarro M.; Pérez H.; Urbina J. A. Toward a Novel Metal-Based Chemotherapy against Tropical Diseases. 2. Synthesis and Antimalarial Activity in Vitro and in Vivo of New Ruthenium-and Rhodium-Chloroquine Complexes. J. Med. Chem. 1996, 39 (5), 1095–1099. 10.1021/jm950729w. 8676344

[ref14] Fletcher N. C. Chiral 2,2′-Bipyridines: Ligands for Asymmetric Induction. J. Chem. Soc., Perkin Trans. 1 2002, (16), 1831–1842. 10.1039/B204272J.

[ref15] Bednářová E.; Dračínský M.; Malatinec Š.; Císařová I.; Lamaty F.; Kotora M. Synthesis of a Bolm’s 2,2′-Bipyridine Ligand Analogue and Its Applications. Adv. Synth. Catal. 2018, 360 (15), 2869–2878. 10.1002/adsc.201800452.

[ref16] Chan H. L.; Ma D. L.; Yang M.; Che C. M. Synthesis and Biological Activity of a Platinum(II) 6-Phenyl-2,2′-bipyridine Complex and Its Dimeric Analogue. ChemBioChem 2003, 4 (1), 62–68. 10.1002/cbic.200390015. 12512077

[ref17] Alp M.; Yurdakul S.; Erdem B. Experimental and Theoretical Vibrational Spectroscopic Investigations, DFT Quantum Chemical Analysis, Biological Activities and Molecular Docking on 4,4′-Dimethoxy-2,2′-bipyridine. J. Mol. Struct. 2022, 1260, 132846 10.1016/j.molstruc.2022.132846.

[ref18] Chellan P.; Avery V. M.; Duffy S.; Land K. M.; Tam C. C.; Kim J. H.; Cheng L. W.; Romero-Canelón I.; Sadler P. J. Bioactive Half-Sandwich Rh and Ir Bipyridyl Complexes Containing Artemisinin. J. Inorg. Biochem. 2021, 219, 111408 10.1016/j.jinorgbio.2021.111408. 33826972

[ref19] Egan T. J.; Koch K. R.; Swan P. L.; Clarkson C.; Van Schalkwyk D. A.; Smith P. J. In Vitro Antimalarial Activity of a Series of Cationic 2,2′-Bipyridyl- and 1,10-Phenanthrolineplatinum(II) Benzoylthiourea Complexes. J. Med. Chem. 2004, 47 (11), 2926–2934. 10.1021/jm031132g. 15139771

[ref20] McInnes E. J. L.; Farley R. D.; Rowlands C. C.; Welch A. J.; Rovatti L.; Yellowlees L. J. On the Electronic Structure of [Pt(4,4′-X_2_-bipy)Cl_2_]^0/–/2–^: An Electrochemical and Spectroscopic (UV/Vis, EPR, ENDOR) Study. J. Chem. Soc., Dalton Trans. 1999, (23), 4203–4208. 10.1039/a904658e.

[ref21] Morgan G. T.; Burstall F. H. Researches on Residual Affinity and Co-ordination. Part XXXIV. 2:2′-Dipyridyl Platinum Salts. J. Chem. Soc. 1934, 965–971. 10.1039/JR9340000965.

[ref22] Reader J.; van der Watt M. E.; Birkholtz L.-M. Streamlined and Robust Stage-Specific Profiling of Gametocytocidal Compounds Against *Plasmodium falciparum*. Front. Cell Infect. Microbiol. 2022, 12, 926460 10.3389/fcimb.2022.926460. 35846744 PMC9282888

[ref23] Leshabane M.; Dziwornu G. A.; Coertzen D.; Reader J.; Moyo P.; Van Der Watt M.; Chisanga K.; Nsanzubuhoro C.; Ferger R.; Erlank E.; Venter N.; Koekemoer L.; Chibale K.; Birkholtz L. M. Benzimidazole Derivatives Are Potent against Multiple Life Cycle Stages of *Plasmodium falciparum* Malaria Parasites. ACS Infect. Dis. 2021, 7 (7), 1945–1955. 10.1021/acsinfecdis.0c00910. 33673735

[ref24] Reader J.; Botha M.; Theron A.; Lauterbach S. B.; Rossouw C.; Engelbrecht D.; Wepener M.; Smit A.; Leroy D.; Mancama D.; Coetzer T. L.; Birkholtz L. M. Nowhere to Hide: Interrogating Different Metabolic Parameters of *Plasmodium falciparum* Gametocytes in a Transmission Blocking Drug Discovery Pipeline towards Malaria Elimination. Malar. J. 2015, 14 (1), 213 10.1186/s12936-015-0718-z. 25994518 PMC4449569

[ref25] Paquet T.; Le Manach C.; Cabrera D. G.; Younis Y.; Henrich P. P.; Abraham T. S.; Lee M. C. S.; Basak R.; Ghidelli-Disse S.; Lafuente-Monasterio M. J.; Bantscheff M.; Ruecker A.; Blagborough A. M.; Zakutansky S. E.; Zeeman A.-M.; White K. L.; Shackleford D. M.; Mannila J.; Morizzi J.; Scheurer C.; Angulo-Barturen I.; Martínez M. S.; Ferrer S.; Sanz L. M.; Gamo F. J.; Reader J.; Botha M.; Dechering K. J.; Sauerwein R. W.; Tungtaeng A.; Vanachayangkul P.; Lim C. S.; Burrows J.; Witty M. J.; Marsh K. C.; Bodenreider C.; Rochford R.; Solapure S. M.; Jiménez-Díaz M. B.; Wittlin S.; Charman S. A.; Donini C.; Campo B.; Birkholtz L.-M.; Hanson K. K.; Drewes G.; Kocken C. H. M.; Delves M. J.; Leroy D.; Fidock D. A.; Waterson D.; Street L. J.; Chibale K. Antimalarial Efficacy of MMV390048, an Inhibitor of Plasmodium Phosphatidylinositol 4-Kinase. Sci. Transl. Med. 2017, 9 (387), eaad9735 10.1126/scitranslmed.aad9735. 28446690 PMC5731459

[ref26] Hidaka M.; Gotoh A.; Shimizu T.; Minamisawa K.; Imamura H.; Uchida T. Visualization of NO3-/NO2- Dynamics in Living Cells by Fluorescence Resonance Energy Transfer (FRET) Imaging Employing a Rhizobial Two-Component Regulatory System. J. Biol. Chem. 2016, 291 (5), 2260–2269. 10.1074/jbc.M115.687632. 26631727 PMC4732209

[ref27] Milton-Laskibar I.; Martínez J. A.; Portillo M. P. Current Knowledge on Beetroot Bioactive Compounds: Role of Nitrate and Betalains in Health and Disease. Foods 2021, 10 (6), 1314 10.3390/foods10061314. 34200431 PMC8229785

[ref28] Corbett Y.; D’Alessandro S.; Parapini S.; Scaccabarozzi D.; Kalantari P.; Zava S.; Giavarini F.; Caruso D.; Colombo I.; Egan T. J.; Basilico N. Interplay between *Plasmodium falciparum* Haemozoin and L-Arginine: Implication for Nitric Oxide Production. Malar J. 2018, 17 (1), 456 10.1186/s12936-018-2602-0. 30522493 PMC6282336

[ref29] Duffy S.; Avery V. M. Identification of Inhibitors of *Plasmodium falciparum* Gametocyte Development. Malar. J. 2013, 12 (1), 408 10.1186/1475-2875-12-408. 24206914 PMC3842684

[ref30] Klonis N.; Xie S. C.; McCaw J. M.; Crespo-Ortiz M. P.; Zaloumis S. G.; Simpson J. A.; Tilley L. Altered Temporal Response of Malaria Parasites Determines Differential Sensitivity to Artemisinin. Proc. Natl. Acad. Sci. U. S. A. 2013, 110 (13), 5157–5162. 10.1073/pnas.1217452110. 23431146 PMC3612604

[ref31] Naude M.; van Heerden A.; Reader J.; van der Watt M.; Niemand J.; Joubert D.; Siciliano G.; Alano P.; Njoroge M.; Chibale K.; Herreros E.; Leroy D.; Birkholtz L.-M. Eliminating Malaria Transmission Requires Targeting Immature and Mature Gametocytes through Lipoidal Uptake of Antimalarials. Nat. Commun. 2024, 15 (1), 9896 10.1038/s41467-024-54144-x. 39548094 PMC11568134

[ref32] Barnes-Seeman D.; Jain M.; Bell L.; Ferreira S.; Cohen S.; Chen X. H.; Amin J.; Snodgrass B.; Hatsis P. Metabolically Stable Tert-Butyl Replacement. ACS Med. Chem. Lett. 2013, 4 (6), 514–516. 10.1021/ml400045j. 24900702 PMC4027455

[ref33] Minić A.; van de Walle T.; van Hecke K.; Combrinck J.; Smith P. J.; Chibale K.; D’hooghe M. Design and Synthesis of Novel Ferrocene-Quinoline Conjugates and Evaluation of Their Electrochemical and Antiplasmodium Properties. Eur. J. Med. Chem. 2020, 187 (2020), 111963 10.1016/j.ejmech.2019.111963. 31865015

[ref34] Wani W. A.; Jameel E.; Baig U.; Mumtazuddin S.; Hun L. T. Ferroquine and Its Derivatives: New Generation of Antimalarial Agents. Eur. J. Med. Chem. 2015, 101, 534–551. 10.1016/j.ejmech.2015.07.009. 26188909 PMC7115395

[ref35] Coban B.; Tekin I. O.; Sengul A.; Yildiz U.; Kocak I.; Sevinc N. DNA Studies of Newly Synthesized Heteroleptic Platinum(II) Complexes [Pt(Bpy)(Iip)]^2+^ and [Pt(Bpy)(Miip)]^2+^. J. Biol. Inorg. Chem. 2016, 21 (2), 163–175. 10.1007/s00775-015-1317-8. 26626200

[ref36] Brown A. R.; Guo Z.; Mosselmans F. W. J.; Parsons S.; Schröder M.; Yellowlees L. J. Structural and Voltammetric Studies on the Reduction of the Bis(2,2′-bipyridyl)platinum(II) Cation in Aprotic Media. J. Am. Chem. Soc. 1998, 120 (34), 8805–8811. 10.1021/ja981670w.

[ref37] Jansen B. A. J.; Brouwer J.; Reedijk J. Glutathione Induces Cellular Resistance against Cationic Dinuclear Platinum Anticancer Drugs. J. Inorg. Biochem. 2002, 89 (3–4), 197–202. 10.1016/S0162-0134(02)00381-1. 12062123

[ref38] Nguyen H. D.; Do L. H. Taming Glutathione Potentiates Metallodrug Action. Curr. Opin. Chem. Biol. 2022, 71, 102213 10.1016/j.cbpa.2022.102213. 36206677 PMC9759795

[ref39] Patzewitz E.-M.; Salcedo-Sora J. E.; Wong E. H.; Sethia S.; Stocks P. A.; Maughan S. C.; Murray J. A. H.; Krishna S.; Bray P. G.; Ward S. A.; Müller S. Glutathione Transport: A New Role for PfCRT in Chloroquine Resistance. Antioxid. Redox Signaling 2013, 19 (7), 683–695. 10.1089/ars.2012.4625. PMC3739961 23256874

[ref40] Kanyora A. K.; Omondi R. O.; Ongoma P.; Omolo J. O.; Welsh A.; Prince S.; Gichumbi J.; Mambanda A.; Smith G. S. Mononuclear H6-Arene Ruthenium(II) Complexes with Pyrazolyl–Pyridazine Ligands: Synthesis, CT-DNA Binding, Reactivity towards Glutathione, and Cytotoxicity. J Biol Inorg Chem 2024, 29 (2), 251–264. 10.1007/s00775-024-02043-3. 38494554

[ref41] Noronha V.; Burtness B.; Murren J.; Duffy T. P. Oxaliplatin Induces a Delayed Immune-Mediated Hemolytic Anemia: A Case Report and Review of the Literature. Clin. Colorectal Cancer 2005, 5 (4), 283–286. 10.3816/CCC.2005.n.041. 16356307

[ref42] Martinho N.; Santos T. C. B.; Florindo H. F.; Silva L. C. Cisplatin-Membrane Interactions and Their Influence on Platinum Complexes Activity and Toxicity. Front. Physiol. 2019, 9, 1898 10.3389/fphys.2018.01898. 30687116 PMC6336831

