# Supplementary material for: Synthesis and SAR Studies of Acyl-Thiourea Platinum(II) Complexes Yield Analogs with Dual-Stage Antiplasmodium Activity
Source: ACS Med Chem Lett. 2025 Feb 17;16(3):428–35. doi: 10.1021/acsmedchemlett.4c00545 (PMC11912270; doi:10.1021/acsmedchemlett.4c00545)
Supplement: Supplementary file 1 — ml4c00545_si_001.pdf [file ml4c00545_si_001.pdf]

Synthesis and SAR studies of acyl-thiourea platinum(II) complexes yield analogs with dual-stage antiplasmodium activity

Fatima-Zahra Ishmail[#], Dina Coertzen[‡], Sizwe Tshabalala[‡], Meta Leshabane[‡], Shante da Rocha[‡], Mathew Njoroge[||], Liezl Gibhard[||], Lyn-Marie Birkholtz[‡,¬,⸗], John G. Woodland[#,||,†], Timothy J. Egan[#,†], Kathryn J. Wicht[#,||,†,*] and Kelly Chibale[#,||,†,*]

[#]Department of Chemistry, University of Cape Town, Rondebosch 7701, South Africa
[‡]Department of Biochemistry, Genetics and Microbiology, Institute for Sustainable Malaria Control, University of Pretoria, Hatfield 0028, South Africa
[¬] Institute for Sustainable Malaria Control, School of Public Health and Health Systems, University of Pretoria, Hatfield 0028, South Africa
[⸗]Department of Biochemistry, Stellenbosch University, Stellenbosch, Matieland, 7602, South Africa
||Holistic Drug Discovery and Development (H3D) Centre, University of Cape Town, Rondebosch 7701, South Africa
†South African Medical Research Council Drug Discovery and Development Research Unit, Institute of Infectious Disease and Molecular Medicine, University of Cape Town, Observatory 7925, South Africa

* Corresponding authors: kathryn.wicht@uct.ac.za; kelly.chibale@uct.ac.za

Table of Contents

1. Experimental 6
1.1. Spectroscopic and analytical methods 6
1.2. Synthesis and characterization 6
1.2.1. *N*4,*N*4'-dimethyl-[2,2'-bipyridine]-4,4'-dicarboxamide (1a) 6
1.2.2. Dimethyl [2,2'-bipyridine]-4,4'-dicarboxylate (1b) 7
1.2.3. General procedure for synthesis of acyl thiourea ligands 7
1.2.3.1. *N*-(bis(2-hydroxyethyl)carbamothioyl)benzamide (2a) 7
1.2.3.2. *N*-(bis(2-hydroxyethyl)carbamothioyl)-4-methylbenzamide (2b) 7
1.2.3.3. *N*-(bis(2-hydroxyethyl)carbamothioyl)-4-methoxybenzamide (2c) 8
1.2.3.4. *N*-(bis(2-hydroxyethyl)carbamothioyl)-4-chlorobenzamide (2d) 8
1.2.4. General synthesis of Pt(diimine)Cl_2 complexes (3a-3l) 8
1.2.4.1. 4,4'-Di-*tert*-butyl-2,2'-bipyridyldichloroplatinum(II) (3a) 8
1.2.4.2. 4,4'-Dimethyl-2,2'-bipyridyldichloroplatinum(II) (3b) 9
1.2.4.3. 2,2'-Bipyridyldichloroplatinum(II) (3c) 9
1.2.4.4. 4,4'-Dimethoxy-2,2'-bipyridyldichloroplatinum(II) (3d) 9
1.2.4.5. 4,4'-Dihydroxy-2,2'-bipyridyldichloroplatinum(II) (3e) 9

1.2.4.6. 4,4’-Diamino-2,2’-bipyridyldichloroplatinum(II) (3f)....9
1.2.4.7. 4,4’-Dichloro-2,2’-bipyridyldichloroplatinum(II) (3g)....9
1.2.4.8. 4,4’-Bis(trifluoromethyl) -2,2’-bipyridyldichloroplatinum(II) (3h) 10
1.2.4.9. *N*4,*N*4'-dimethyl-[2,2'-bipyridine]-4,4'-dicarboxamide dichloroplatinum(II) (3i) 10
1.2.4.10. *N*4,*N*4’-dimethyl-[2,2'-bipyridine]-4,4'-dicarboxylate dichloroplatinum(II) (3j) 10
1.2.4.11. 5,5’-Dimethyl-2,2’-bipyridyldichloroplatinum(II) (3k)....10
1.2.4.12. 6,6’-Dimethyl-2,2’-bipyridyldichloroplatinum(II) (3l)....10
1.2.5. Synthesis of [Pt(diimine)(L-O,S)]$^+$ complexes 11
General procedure 11
1.2.5.1. (*N*-Benzoyl-*N*’,*N*’-di(2-hydroxyethylthioureato)-*S*,*O*)(4,4’-di-*tert*-butyl-2,2’-bipyridyl)platinum(II) Chloride (C1) 11
1.2.5.2. (*N*-Benzoyl-*N*’,*N*’-di(2-hydroxyethylthioureato)-*S*,*O*)(4,4’-dimethyl-2,2’-bipyridyl)platinum(II) Chloride (C2)....11
1.2.5.3. (*N*-Benzoyl-*N*’,*N*’-di(2-hydroxyethylthioureato)-*S*,*O*)(2,2’-bipyridyl)platinum(II) Chloride (C3)....12
1.2.5.4. (*N*-Benzoyl-*N*’,*N*’-di(2-hydroxyethylthioureato)-*S*,*O*)(4,4’-dimethoxyl-2,2’-bipyridyl)platinum(II) Chloride (C4)....12
1.2.5.5. (*N*-Benzoyl-*N*’,*N*’-di(2-hydroxyethylthioureato)-*S*,*O*)(4,4’-dihydroxy-2,2’-bipyridyl)platinum(II) Chloride (C5)....13
1.2.5.6. (*N*-Benzoyl-*N*’,*N*’-di(2-hydroxyethylthioureato)-*S*,*O*)(4,4’-diamino-2,2’-bipyridyl)platinum(II) Chloride (C6)....13
1.2.5.7. (*N*-Benzoyl-*N*’,*N*’-di(2-hydroxyethylthioureato)-*S*,*O*)(4,4’-dichloro-2,2’-bipyridyl)platinum(II) Chloride (C7)....13
1.2.5.8. (*N*-Benzoyl-*N*’,*N*’-di(2-hydroxyethylthioureato)-*S*,*O*)(bis(trifluoromethyl)-2,2’-bipyridyl)platinum(II) Chloride (C8) 14
1.2.5.9. (*N*-Benzoyl-*N*’,*N*’-di(2-hydroxyethylthioureato)-*S*,*O*)(*N*4,*N*4’-dimethyl-2,2’-bipyridyl)-4,4’-dicarboxamideplatinum(II) Chloride (C9) 14
1.2.5.10. (*N*-Benzoyl-*N*’,*N*’-di(2-hydroxyethylthioureato)-*S*,*O*)(*N*4,*N*4’-dimethyl-2,2’-bipyridyl)-4,4’-dicarboxylateplatinum(II) Chloride (C10) 14
1.2.5.11. *N*-(4-methyl-Benzoyl-*N*’,*N*’-di(2-hydroxyethylthioureato)-*S*,*O*)(4,4’-di-*tert*-butyl-2,2’-bipyridyl)platinum(II) chloride (C11)....15
1.2.5.12. *N*-(4-methoxy-benzoyl-*N*’,*N*’-di(2-hydroxyethylthioureato)-*S*,*O*)(4,4’-di-*tert*-butyl-2,2’-bipyridyl)platinum(II) Chloride (C12)....15
1.2.5.13. *N*-(4-chloro-Benzoyl-*N*’,*N*’-di(2-hydroxyethylthioureato)-*S*,*O*)(4,4’-di-*tert*-butyl-2,2’-bipyridyl)platinum(II) Chloride (C13)....15
1.2.5.14. (*N*-Benzoyl-*N*’,*N*’-di(2-hydroxyethylthioureato)-*S*,*O*)(5,5’-dimethyl-2,2’-bipyridyl)platinum(II) Chloride (C14)....16
1.2.5.15. (*N*-Benzoyl-*N*’,*N*’-di(2-hydroxyethylthioureato)-*S*,*O*)(6,6’-dimethyl-2,2’-bipyridyl)platinum(II) Chloride (C15)....16

1.2.5.16. (*N*-Benzoyl-*N*’,*N*’-di(2-hydroxyethylthioureato)-*S*,*O*)(4,4’-di-*tert*-butyl-2,2’-bipyridyl)platinum(II) hexafluorophosphate (C16) 16

1.2.5.17. (*N*-Benzoyl-*N*’,*N*’-di(2-hydroxyethylthioureato)-*S*,*O*)(4,4’-di-*tert*-butyl-2,2’-bipyridyl)platinum(II) nitrate (C17) 17

1.3. Cultivation of *Plasmodium falciparum* parasites 17

1.4. *In vitro* activity against ABS parasites 17

1.5. *In vitro* activity against gametocyte stages 18

1.6. *In vitro* cytotoxicity assay 18

1.7. Kinetic solubility by HPLC (pH 6.5) 19

1.8. Microsomal metabolic stability studies 19

1.9. Determination of pharmacokinetic parameters 19

1.10. *In vitro* parallel artificial membrane permeability assay (PAMPA) for permeability determination 20

1.11. Plasma protein binding (PPB) 20

1.12. Cyclic voltammetry 21

1.13. Glutathione binding UV-Vis titrations 21

1.14. Hemolysis assay 22

2. ^{1}H- and ^{13}-C NMR spectra 23

Figure S1. ^{1}H-NMR spectrum of compound **1a** in DMSO-d_6. 23

Figure S2. ^{1}H-NMR spectrum of compound **1b** in DMSO-d_6. 23

Figure S3. ^{1}H-NMR spectrum of compound **2a** in MeOD-d_4. 24

Figure S4. ^{1}H-NMR spectrum of compound **2b** in MeOD-d_4. 24

Figure S5. ^{1}H-NMR spectrum of compound **2c** in DMSO-d_6. 25

Figure S6. ^{1}H-NMR spectrum of compound **3a** in CD_2Cl_2-d_2. 25

Figure S7. ^{1}H-NMR spectrum of compound **3b** in DMSO-d_6. 26

Figure S8. ^{1}H-NMR spectrum of compound **3d** in DMSO-d_6. 26

Figure S9. ^{1}H-NMR spectrum of compound **3e** in DMSO-d_6. 27

Figure S10. ^{1}H-NMR spectrum of compound **3g** in DMSO-d_6. 27

Figure S11. ^{1}H-NMR spectrum of compound **3h** in DMSO-d_6. 28

Figure S12. ^{1}H-NMR spectrum of compound **3i** in DMSO-d_6. 28

Figure S13. ^{1}H-NMR spectrum of compound **3j** in DMSO-d_6. 29

Figure S14. ^{1}H-NMR spectrum of compound **3k** in DMSO-d_6. 29

Figure S15. ^{1}H-NMR spectrum of compound **3l** in DMSO-d_6. 30

Figure S16. ^{1}H-NMR spectrum of compound **C1** in MeOD-d_4. 30

Figure S17. ^{13}C-NMR spectrum of compound **C1** MeOD-d_4. 31

Figure S18. ^{1}H-NMR spectrum of compound **C2** in D_2O/CD_3CN-d_3. 31

Figure S19. ^{13}C-NMR spectrum of compound **C2** in D_2O. 32

Figure S20. ^{1}H-NMR spectrum of compound **C3** in DMSO-d_6. 32
Figure S21. ^{13}C-NMR spectrum of compound **C3** in DMSO-d_6. 33
Figure S22. ^{1}H-NMR spectrum of compound **C4** in DMSO-d_6. 33
Figure S23. ^{13}C-NMR spectrum of compound **C4** in DMSO-d_6. 34
Figure S24. ^{1}H-NMR spectrum of compound **C5** in DMSO-d_6. 34
Figure S25. ^{1}H-NMR spectrum of compound **C6** in CD_3CN-d_3/D2O. 35
Figure S26. ^{1}H-NMR spectrum of compound **C7** in MeOD-d_4. 35
Figure S27. ^{13}C-NMR spectrum of compound **C7** in DMSO-d_6. 36
Figure S28. ^{1}H-NMR spectrum of compound **C8** in MeOD-d_4. 36
Figure S29. ^{1}H-NMR spectrum of compound **C9** in DMSO-d_6. 37
Figure S30. ^{1}H-NMR spectrum of compound **C11** in MeOD-d_4. 37
Figure S31. ^{1}H-NMR spectrum of compound **C12** in DMSO-d_6. 38
Figure S32. ^{1}H-NMR spectrum of compound **C13** in DMSO-d_6. 38
Figure S33. ^{1}H-NMR spectrum of compound **C14** in D_2O/CD_3CN-d_3. 39
Figure S34. ^{1}H NMR spectrum of compound **C15** in DMSO-d_6. 39
Figure S35. ^{1}H NMR spectrum of compound **C16** in DMSO-d_6. 40
Figure S36. ^{19}F-NMR of compound **C16** in DMSO-d_6. 40
Figure S37. ^{1}H-NMR of compound **C17** in DMSO-d_6. 40
3. Cyclic voltammograms 41
Figure S38. Cyclic voltammogram of compound **C1** 41
Figure S39. Cyclic voltammogram of compound **C2** 41
Figure S40. Cyclic voltammogram of compound **C3** 42
Figure S41. Cyclic voltammogram of compound **C4** 42
Figure S42. Cyclic voltammogram of compound **C6** 43
Figure S43. Cyclic voltammogram of compound **C8** 43
Figure S44. Cyclic voltammogram of compound **C11** 44
Figure S45. Cyclic voltammogram of compound **C12** 44
Figure S46. Cyclic voltammogram of compound **C13** 44
4. Glutathione binding and hemolysis studies 44
Figure S47. Representative spectrum showing changes in the UV-visible absorption spectrum of **C1** with increasing amounts of GSH (0.4 M) in PBS (pH 7.2) at 25 °C. Right side panel shows the decrease in absorbance at 366 nm with increasing concentrations of GSH. The UV-Vis spectra were corrected for by dilution. 45
Figure S48. Representative spectrum showing changes in the UV-visible absorption spectrum of **C6** with increasing amounts of GSH (0.4 M) in PBS (pH 7.2) at 25 °C. Right side panel shows the decrease in absorbance at 366 nm with increasing concentrations of GSH. The UV-Vis spectra were corrected for dilution. 45

Table S1. Log*K* values for the binding of GSH to platinum(II) complexes. Experiments were conducted in triplicate (n = 3) with the mean and standard error of the mean (SEM) reported. 45

Figure S49. Graphs showing the percentage hemolysis induced in RBCs by compounds **C1**, **C6** and **C17** after 1 hour and 72 hours of incubation. The percentage hemolysis induced by chloroquine (negative control) and Triton X-100 (positive control) are also shown. 46

Table S2: Dual point activity of platinum(II) complexes against ABS parasites, early- and late-stage gametocytes. Values are averaged from technical triplicates performed for a single biological repeat. 46

5. References 47

1. Experimental

All reagents were purchased from Merck and Combi-Blocks and used without further purification. All solvents were of analytical grade. In the case of reactions done under argon, anhydrous solvents were purchased from Merck, or the solvent was dried over molecular sieves. All reactions were monitored via thin-layer chromatography (TLC) using aluminium-backed, precoated silica-gel 60 F254 plates (Merck). TLC plates were observed under ultraviolet light at 254 nm. All compounds synthesized were dried under a high vacuum. All deuterated solvents used for NMR spectroscopy were purchased from Sigma-Aldrich. Where purification was carried out via reverse-phase column chromatography, it was performed using a C-18 (10/25 g cartridge) column. All compounds reported and tested for their biological activity are >95% pure as indicated by HPLC analysis.

1.1. Spectroscopic and analytical methods

Nuclear magnetic resonance (NMR) spectra were recorded on Bruker TopSpin GmbH 600 MHz (1H at 600.22 MHz; ^{13}C at 100.65 MHz) or Varian Mercury 300 (^{1}H at 300.08 MHz) spectrometers, equipped with a Bruker BioSpin GmbH casing and sample injector at 30 °C. Chemical shifts and *J*-couplings are reported in ppm and Hz, respectively. NMR spectra were recorded using either deuterated dimethyl sulfoxide (DMSO-d_6), deuterated dichloromethane (CD_2Cl_2), deuterated methanol (MeOD-d_4), deuterium oxide (D_2O) or deuterated acetonitrile (CD_3CN-d_3). Where ^{1}H- and ^{13}C-NMR data are not provided, compounds were not soluble enough to obtain a spectrum of representable resolution.

1.2. Synthesis and characterization

1.2.1. *N*4,*N*4'-dimethyl-[2,2'-bipyridine]-4,4'-dicarboxamide (1a)

Triethylamine (51.7 µL, 3.71 mmol, 4 eq.) was added to a solution of [2,2'-bipyridine]-4,4'-dicarboxylic acid (226 mg, 0.928 mmol, 1 eq.) in DMF (5 mL). The solution was stirred for 15 minutes before adding HATU (1.41 g, 3.71 mmol, 4 eq.). The solution was allowed to stir for a further 30 minutes. Methylamine in THF (2 M, 1.86 mL, 3.71 mmol, 4 eq.) was added and the reaction mixture was allowed to stir at room temperature for 2 hours. The DMF was reduced using rotary evaporation and MeOH (~5 mL) added. Compound **2a** was collected using suction filtration and washed with MeOH. Compound **2a** was isolated as an off-white solid (225 mg, 90%); ^{1}H NMR (400 MHz, DMSO-d_6) δ 8.90 (d, *J* = 4.8 Hz, 2H), 8.87 (d, *J* = 5.0 Hz, 2H),

8.79 (br s, 2H), 7.85 (dd, J = 1.8, 5.0 Hz, 2H), 2.84 (d, J = 4.5 Hz, 6H). HPLC-MS (ESI): Purity = 99%, t_R = 0.598 min, m/z $[M+H]^+$ = 271.1, calculated mass $[M+H]^+$ = 271.3.

1.2.2. Dimethyl [2,2'-bipyridine]-4,4'-dicarboxylate (1b)

Triethylamine (34.7 µL, 2.49 mmol, 4 eq.) was added to a solution of [2,2'-bipyridine]-4,4'-dicarboxylic acid (304 mg, 1.24 mmol, 1 eq.) in MeOH (5 mL). The solution was stirred for 15 minutes before adding HATU (1.89 g, 2.49 mmol, 4 eq.). The reaction mixture was allowed to stir at 50 °C for 24 hours. The resulting precipitate was collected using suction filtration. Compound **2b** was isolated as a white solid (283 mg, 84%); ^{1}H NMR (300 MHz, DMSO-d_6) δ 8.92 (d, J = 4.9 Hz, 2H), 8.85 (d, J = 1.6 Hz, 2H), 7.91 (dd, J = 1.6, 4.9 Hz, 2H), 3.30 (s, 6H). HPLC-MS (ESI): Purity = 97%, t_R = 1.003 min, m/z $[M+H]^+$ = 273.1, calculated mass $[M+H]^+$ = 273.3.

1.2.3. *General procedure for synthesis of acyl thiourea ligands*

To a 50-mL round bottom flask (RBF) equipped with a magnetic stirrer and septum with a needle connected to a balloon containing nitrogen, the relevant benzoyl isothiocyanate (1 eq.) followed by anhydrous DCM (2 mL) was added. The mixture was cooled in an ice-water bath at a temperature <5° C. Diethanolamine (1 eq.) in anhydrous DCM (2 mL) was added slowly over 15 min. The ice-water bath was removed, and the reaction mixture was stirred under nitrogen for 4-20 h. The resulting precipitate was collected using suction filtration and washed with cold DCM.

1.2.3.1. *N*-(bis(2-hydroxyethyl)carbamothioyl)benzamide (2a)

Benzoyl isothiocyanate (1 mL, 7.44 mmol, 1 eq.) and diethanolamine (1.10 mL, 7.44 mmol, 1 eq.) were stirred at 24 °C for 20 h. Compound **1a** was isolated as a beige solid (1.66 g, 83%); ^{1}H-NMR (600 MHz, MeOD-d_4) δ 7.88 (d, J = 8.5 Hz, 2H, 7.57 (t, J = 8.1 Hz, 1H), 7.47 (t, J = 7.9 Hz, 2H) 4.06 (br s, 2H), 3.96 (br s, 2H), 3.84 (br s, 4H); HPLC-MS (ESI): Purity = 98%, t_R = 0.387 min, m/z $[M-H]^+$ = 267.0, calculated mass $[M-H]^+$ = 267.3.

1.2.3.2. *N*-(bis(2-hydroxyethyl)carbamothioyl)-4-methylbenzamide (2b)

4-Methylbenzoyl isothiocyanate (0.1 mL, 0.654 mmol, 1 eq.) and diethanolamine (1.10 mL, 0.654 mmol, 1 eq.) were stirred at 21 °C for 20 h. Compound **1b** was isolated as a beige solid (246 mg, 75%); ^{1}H NMR (300 MHz, MeOD-d_4) δ 7.70 (d, J = 8.3 Hz, 2H), 7.21 (d, J = 7.9 Hz,

2H), 3.97 (t, *J* = 6.1 Hz, 2H), 3.87 (t, *J* = 5.8 Hz, 2H), 3.76 (s, 4H), 2.31 (s, 3H). HPLC-MS (ESI): Purity = 98%, t_R = 0.702 min, *m/z* $[M+H]^+$ = 283.1, calculated mass $[M+H]^+$ = 283.4.

1.2.3.3. *N*-(bis(2-hydroxyethyl)carbamothioyl)-4-methoxybenzamide (2c)

4-Methoxybenzoyl isothiocyanate (0.1 mL, 0.600 mmol, 1 eq.) and diethanolamine (0.06 mL, 0.600 mmol, 1 eq.) were stirred at 20 °C for 20 h. Compound **1c** was isolated as an off-white solid (136 mg, 76%); ^{1}H NMR (300 MHz, DMSO-d_6) δ 7.86 (d, *J* = 9.0 Hz, 2H), 7.04 (d, *J* = 9.0 Hz, 2H), 5.67 (br s, 1H), 4.89 (t, *J* = 5.2, 5.2 Hz, 1H), 3.96 (t, *J* = 6.2, 6.2 Hz, 2H), 3.98 (s, 3H), 3.76 – 3.69 (m, 6H). HPLC-MS (ESI): Purity = 97%, t_R = 0.654 min, *m/z* $[M+H]^+$ = 299.1, calculated mass $[M+H]^+$ = 299.4.

1.2.3.4. *N*-(bis(2-hydroxyethyl)carbamothioyl)-4-chlorobenzamide (2d)

4-Chlorobenzoyl isothiocyanate (204 mg, 1.03 mmol, 1 eq.) and diethanolamine (0.10 mL, 1.03 mmol, 1 eq.) were stirred at 20 °C for 4 h. Compound 4 was isolated as an off-white solid (289 mg, 92%). Crude yield: 279 mg, 89%.

1.2.4. General synthesis of Pt(diimine)Cl_2 complexes (3a-3l)

The appropriate diimine (1 eq.) was added to a stirring solution of potassium tetrachloroplatinate (1 eq.) dissolved in H_2O (2 mL) and then either acidified with HCl (1 mL, 4 M) or left neutral. The mixture was allowed to reflux for 2 h or 24 h and the resulting powder was collected via suction filtration. The product was washed with water and dried under a vacuum. Where the crude product was isolated, no spectral analysis was done, and the crude product was used in the next step. Where the product was insoluble in the available deuterated solvents, the compound was also taken forward to the next synthetic step without further characterization.

1.2.4.1. 4,4'-Di-*tert*-butyl-2,2'-bipyridyldichloroplatinum(II) (3a)

4,4'-Di-*tert*-butyl-2,2'-dipyridyl (70.2 mg, 0.259 mmol, 1 eq.) was added to a stirring solution of potassium tetrachloroplatinate (108 mg, 0.259 mmol, 1 eq). Yield: 65 mg, 47%; ^{1}H-NMR (600 MHz, CD_2Cl_2) δ 9.47 (d, *J* = 6.3 Hz, 1H), 7.92 (d, *J* = 2.2 Hz, 1H), 7.55 (dd, *J* = 6.3, 2.1 Hz, 1H), 1.45 (s, 9H); HR-ESI MS: (*m/z*) $[M+Cl]^+$ = 569.06, calculated mass $[M+Cl]^+$ = 569.84.

1.2.4.2. 4,4’-Dimethyl-2,2’-bipyridyldichloroplatinum(II) (3b)

4,4'-Dimethyl-2,2'-bipyridine (2) (166 mg, 0.900 mmol, 1 eq.) was added to a stirring solution of potassium tetrachloroplatinate (374 mg, 0.900 mmol, 1 eq.). Crude yield: 256 mg, 63%. ^{1}H NMR (300 MHz, DMSO-d_6) δ 9.24 (d, J = 6.2 Hz, 2H), 8.45 (d, J = 6.1 Hz, 2H), 7.67 (d, J = 5.9 Hz, 2H), 2.53 (s, 6H).

1.2.4.3. 2,2’-Bipyridyldichloroplatinum(II) (3c)

2,2’-Bipyridine (339 mg, 2.17 mmol, 1 eq.) was added to a stirring solution of potassium tetrachloroplatinate (905 mg, 2.17 mmol, 1 eq). Crude yield: 846 mg, 92%.

1.2.4.4. 4,4’-Dimethoxy-2,2’-bipyridyldichloroplatinum(II) (3d)

4,4’-Dimethoxy-2,2’-bipyridine (81.2 mg, 0.371 mmol, 1 eq.) was added to a stirring solution of potassium tetrachloroplatinate (154 mg, 0.371 mmol, 1 eq). Crude yield: 150 mg, 84%; ^{1}H NMR (400 MHz, DMSO-d_6) δ 9.15 (d, J = 6.8 Hz, 2H), 8.20 (d, J = 2.9 Hz, 2H), 7.42 (dd, J = 2.8, 6.9 Hz, 2H), 4.06 (s, 6H).

1.2.4.5. 4,4’-Dihydroxy-2,2’-bipyridyldichloroplatinum(II) (3e)

4,4’-Dihydroxy-2,2’-bipyridine (114 mg, 0.605 mmol, 1 eq.) was added to a stirring solution of potassium tetrachloroplatinate (251 mg, 0. 605 mmol, 1 eq). Crude yield: 123 mg, 45%; ^{1}H NMR (400 MHz, DMSO-d_6) δ 9.04 (d, J = 7.1 Hz, 2H), 7.75 (d, J = 3.2 Hz, 2H), 7.15 (dd, J = 2.9, 7.2 Hz, 1H).

1.2.4.6. 4,4’-Diamino-2,2’-bipyridyldichloroplatinum(II) (3f)

4,4’-Diamino-2,2’-bipyridine (96 mg, 0.515 mmol, 1 eq.) was added to a stirring solution of potassium tetrachloroplatinate (214 mg, 0. 515 mmol, 1 eq). Crude yield: 123 mg, 53%.

1.2.4.7. 4,4’-Dichloro-2,2’-bipyridyldichloroplatinum(II) (3g)

4,4’-Dichloro-2,2’-bipyridine (105 mg, 0.466 mmol, 1 eq.) was added to a stirring solution of potassium tetrachloroplatinate (194 mg, 0.466 mmol, 1 eq). Crude yield: 201 mg, 88%; ^{1}H

NMR (600 MHz, DMSO-d_6) δ 9.39 (d, *J* = 6.4 Hz, 2H), 8.89 (d, *J* = 2.5 Hz, 2H), 7.99 (dd, *J* = 2.4, 6.4 Hz, 2H).

1.2.4.8. 4,4'-Bis(trifluoromethyl) -2,2'-bipyridyldichloroplatinum(II) (3h)

4,4'-bis(trifluoromethyl) -2,2'-bipyridine (115 mg, 0.395 mmol, 1 eq.) was added to a stirring solution of potassium tetrachloroplatinate (164 mg, 0.395 mmol, 1 eq). Crude yield: 124 mg, 56%; ^{1}H NMR (300 MHz, DMSO-d_6) δ 9.8 (d, *J* = 6.0 Hz, 2H), 9.31 (d, *J* = 1.92 Hz, 2H), 8.29 (dd, *J* = 1.9, 6.3 Hz, 2H).

1.2.4.9. *N*4,*N*4'-dimethyl-[2,2'-bipyridine]-4,4'-dicarboxamide dichloroplatinum(II) (3i)

*N*4,*N*4'-dimethyl-[2,2'-bipyridine]-4,4'-dicarboxamide (96 mg, 0.516 mmol, 1 eq.) was added to a stirring solution of potassium tetrachloroplatinate (214 mg, 0.516 mmol, 1 eq). Crude yield: 124 mg, 56%; ^{1}H NMR (400 MHz, DMSO-d_6) δ 9.63 (d, *J* = 6.1 Hz, 2H), 9.14 (d, *J* = 4.6 Hz, 2H), 8.90 (d, *J* = 2.1 Hz, 2H), 8.18 (dd, *J* = 6.1, 2.0 Hz, 1H), 2.90 (d, *J* = 4.5 Hz, 6H).

1.2.4.10.*N*4,*N*4'-dimethyl-[2,2'-bipyridine]-4,4'-dicarboxylate dichloroplatinum(II) (3j)

dimethyl-[2,2'-bipyridine]-4,4'-dicarboxylate (101 mg, 00.371 mmol, 1 eq.) was added to a stirring solution of potassium tetrachloroplatinate (154 mg, 0.371 mmol, 1 eq). Crude yield: 124 mg, 56%; ^{1}H NMR (300 MHz, DMSO-d_6) δ 9.74 (d, *J* = 6.1 Hz, 2H), 9.10 (d, *J* = 1.8 Hz, 2H), 8.96 (d, *J* = 5.0 Hz, 1H), 8.86 (d, *J* = 2.6 Hz, 1H), 8.27 (dd, *J* = 1.8, 6.1 Hz, 2H), 7.96 (dd, *J*= 5.0 Hz, 1.7 Hz, 1H) 4.01 (s, 3H), 3.97 (s, 3H)

1.2.4.11.5,5'-Dimethyl-2,2'-bipyridyldichloroplatinum(II) (3k)

5,5'-Dimethyl-2,2'-bipyridine (79.7 mg, 0.433 mmol, 1 eq.) was added to a stirring solution of potassium tetrachloroplatinate (180 mg, 0.433 mmol, 1 eq.). Crude yield: 105 mg, 54%. ^{1}H NMR (300 MHz, DMSO-d_6) δ 9.22 (d, *J* = 2.1 Hz, 2H), 8.4 (d, *J* = 8.3 Hz, 2H), 8.21 (dd, *J* = 2.0, 8.3 Hz, 2H), 2.48 (s, 6H).

1.2.4.12.6,6'-Dimethyl-2,2'-bipyridyldichloroplatinum(II) (3l)

6,6'-Dimethyl-2,2'-bipyridine (84.1 mg, 0.456 mmol, 1 eq.) was added to a stirring solution of potassium tetrachloroplatinate (189 mg, 0.456 mmol, 1 eq.). Crude yield: 143 mg.^{1}H NMR

(300 MHz, DMSO-d_6) δ 8.17 (d, *J* = 7.9 Hz, 2H), 7.80 (t, *J* = 7.7 Hz, 2H), 7.29 (d, *J* = 7.6 Hz, 2H), 2.55 (s, 6H).

1.2.5. Synthesis of [Pt(diimine)(L-O,S)]⁺ complexes

General procedure

Compound **2a-d** (1.1 eq.) and triethylamine (1.1 eq.) in acetone (1 mL)/dry DMF (4 mL)/MeCN (1 mL) was added dropwise to a stirring solution of the respective precursor complexes **3a-3l (**1 eq.) in acetone (1 mL)/dry DMF (14 mL)/MeCN (1 mL). The mixture was refluxed at 60 °C or 80 °C for 4 h (**C1**-**C4**) or 24 h (**C5- C16**) and then cooled. The precipitate was collected via suction filtration while being washed with acetone. Where the complex was collected as a crude product, it was purified using reverse phase column chromatography using either MeOH: H_2O or MeCN: H_2O as the mobile phase.

1.2.5.1. (*N*-Benzoyl-*N'*,*N'*-di(2-hydroxyethylthioureato)-*S*,*O*)(4,4'-di-*tert*-butyl-2,2'-bipyridyl)platinum(II) Chloride (C1)

Compound **2a** and triethylamine (0.033 mL, 0.259 mmol, 1.1 eq.) in acetone were added to a solution of compound **3a** (0.126 g, 0.236 mmol 1 eq.) in acetone. Compound **C1** was isolated as a yellow solid (88.8 mg, 47 %); ^{1}H-NMR (600 MHz, MeOD-d_4 δ 8.92 (d, *J* = 6.0 Hz, 1H), 8.55 (d, *J* = 2.1 Hz, 1H), 8.52 (d, *J* = 6.2 Hz, 1H), 8.45 (d, *J* = 2.2 Hz, 1H), 8.05 (d, *J* = 8.4 Hz, 2H), 7.99 (dd, *J* = 6.0, 2.0 Hz, 1H), 7.64 (dd, *J* = 6.2, 2.2 Hz, 1H), 7.60 (t, *J* = 7.5Hz, 1H), 7.46 (t, *J* = 7.7 Hz, 2H), 4.11 (t, *J* = 5.8 Hz, 2H), 4.04 (q, *J* = 6.0 Hz, 4H), 3.84 (t, *J* = 5.9 Hz, 2H), 1.59 (s, 9H), 1.53 (s, 9H); ^{13}C-NMR (151 MHz, MeOD-d_4) δ 161.65, 159.11, 158.64, 158.55, 149.52, 146.60, 139.96, 135.70, 126.95, 124.39, 121.02, 120.39, 116.62, 116.59, 113.75, 113.23, 51.31, 50.61, 48.47, 46.98, 27.91, 27.72, 21.04, 20.89; HPLC-MS (ESI): Purity = 97%, t_R = 17.370 min HR-ESI MS: (*m/z*) $[M-Cl]^+$ = 730.2451, calculated mass $[M-Cl]^+$ = 730.8; IR (ATR, cm^{-1}): 3325 (broad, weak, OH), 2957 (sharp, weak, N=C=S), 2866 (sharp, weak, N=C=S), 1614 (sharp, weak, C=N), 1500 (sharp, strong, C=C), 1411 (sharp, strong, C-H).

1.2.5.2. (*N*-Benzoyl-*N'*,*N'*-di(2-hydroxyethylthioureato)-*S*,*O*)(4,4'-dimethyl-2,2'-bipyridyl)platinum(II) Chloride (C2)

Compound **2a** (78.9 mg, 0.294 mmol, 1.1 eq.) and triethylamine (40 µL, 0.294 mmol, 1.1 eq.) in dry DMF was added to compound **3b** (119 mg, 0.267 mmol, 1 eq.) in dry DMF. Compound **C2** was isolated as a yellow solid (148 mg, 77%); ^{1}H NMR (600 MHz, D_2O/CD_3CN-d_3) δ 8.47 (d, *J* = 5.5 Hz, 1H), 8.27 (d, *J* = 5.8 Hz, 1H), 8.07 (t, *J* = 7.2 Hz, 1H), 8.00 (br s, 1H), 7.99 (d,

J = 8.0,2H), 7.90 (br s, 1H), 7.82 (t, J = 7.6 Hz, 2H), 7.70 (d, J = 5.6 Hz, 1H), 7.49 (d, J = 5.8 Hz, 1H), 4.28 (t, J = 6.1 Hz, 2H), 4.17 (t, J = 6.0 Hz, 2H), 4.13 (s, 4H), 2.67 (s, 3H), 2.60 (s, 3H). ^{13}C NMR (151 MHz, D_2O) δ 168.62, 166.07, 156.68, 154.74, 153.96, 147.37, 143.30, 134.38, 133.58, 129.61 (2C), 129.30 (2C), 128.71, 125.28, 124.74, 59.67 (2C), 58.67 (2C), 56.53, 55.04, 21.45, 21.13.; HPLC-MS (ESI): Purity = 98%, t_R = 2.31 min, (*m/z*) $[M\text{-}Cl]^+$ = 646.1, calculated mass $[M\text{-}Cl]^+$ = 646.7.

1.2.5.3. (*N*-Benzoyl-*N'*,*N'*-di(2-hydroxyethylthioureato)-*S*,*O*)(2,2'-bipyridyl)platinum(II) Chloride (C3)

Compound **2a** (166 mg, 0.518 mmol, 1.1 eq.) and triethylamine (86 µL, 0.518 mmol, 1.1 eq.) in DMF were added to compound **3c** (237 mg, 0.561 mmol, 1 eq.) Compound **C3** was isolated as a yellow solid (333 mg, 86%); ^{1}H NMR (600 MHz, DMSO-d_6) δ 9.01 (t, J = 6.0 Hz, 1H), 8.69 (d, J = 8.0 Hz, 1H), 8.65 (d, J = 6.0 Hz, 1H), 8.62 (d, J = 7.84 Hz, 1H), 8.48 (t, J = 8.6 Hz, 1H), 8.39 (t, J = 8.6 Hz, 1H), 8.12 (d, J = 6.0 Hz, 2H), 8.08 (t, J = 6.0 Hz, 1H), 7.75 (t, J = 6.6 Hz, 1H), 7.65 (d, J = 6.0 Hz, 1H), 7.56 (t, J = 6.0 Hz, 2H), 5.15 (t, J = 5.4 Hz, 1H), 5.02 (t, J = 5.4 Hz, 1H), 4.03 (t, J = 5.8 Hz, 2H), 4.00 (t, J = 5.9 Hz, 2H), 3.86 (q, J = 5.8 Hz, 6.0 Hz, 2H), 3.71 (q, J = 5.8 Hz, 6.0 Hz, 2H). ^{13}C NMR (151 MHz, DMSO-d_6) δ 169.43, 166.47, 157.87, 154.84, 148.98, 144.74, 142.18, 142.09, 135.00, 133.23, 129.78 (2C), 129.33(2C), 128.90, 128.75, 125.51, 124.95, 59.26, 58.49, 56.92, 55.44.; HPLC-MS (ESI): Purity = 99%, t_R = 2.40 min, (*m/z*) $[M\text{-}Cl]^+$ = 618.1, calculated mass $[M\text{-}Cl]^+$ = 618.6.

1.2.5.4. (*N*-Benzoyl-*N'*,*N'*-di(2-hydroxyethylthioureato)-*S*,*O*)(4,4'-dimethoxyl-2,2'-bipyridyl)platinum(II) Chloride (C4)

Compound **2a** (86.2 mg, 0.321 mmol, 1.1 eq.) and triethylamine (44 µL, 0.321 mmol, 1.1 eq.) in acetone were added to compound **3d** (140 mg, 0.292 mmol, 1 eq.) Compound **C4** was isolated as a yellow solid (333 mg, 86%); ^{1}H NMR (600 MHz, DMSO-d_6) δ 8.52 (d, J = 6.2 Hz, 1H), 8.15 (d, J = 6.0 Hz, 1H), 8.08 (d, J = 8.3 Hz, 2H), 7.95 (d, J = 6.2 Hz, 2H), 7.63 (t, J = 6.7 Hz, 1H), 7.51 (dd, J = 2.8, 6.6 Hz, 1H), 7.47 (t, J = 7.8 Hz, 2H), 7.15 (dd, J = 2.9, 6.8 Hz, 1H), 5.20 (t, J = 5.3 Hz, 1H), 5.07 (t, J = 5.2 Hz, 1H), 3.98 (s, 3H), 3.90 (s, 7H), 3.81 (q, J = 5.8 Hz, 2H), 3.69 (q, J = 5.8 Hz, 2H). ^{13}C NMR (151 MHz, DMSO-d_6) δ 168.85 (d, J = 11.8 Hz), 166.20, 158.81, 156.08, 149.26, 145.41, 135.07, 133.05, 129.56 (2C), 129.21 (2C), 113.99, 113.77, 111.94, 111.39, 59.18 (2C), 58.33 (2C), 57.87 (2C), 56.77, 55.33.; HPLC-MS (ESI): Purity = 98%, t_R = 2.41 min, (*m/z*) $[M\text{-}Cl]^+$ = 678.1, calculated mass $[M\text{-}Cl]^+$ = 678.6.

1.2.5.5. (*N*-Benzoyl-*N*',*N*'-di(2-hydroxyethylthioureato)-*S*,*O*)(4,4'-dihydroxy-2,2'-bipyridyl)platinum(II) Chloride (C5)

Compound **2a** (67.2 mg, 0.50 mmol, 1.1 eq.) and triethylamine (34.9 µL, 0.250 mmol, 1.1 eq.) in acetone were added to compound **3e** (102 mg, 0.228 mmol, 1 eq.) Compound **C4** was isolated as a brown solid (118 mg, 75%); ^{1}H NMR (600 MHz, DMSO-d_6) δ 8.65 (d, J = 6.5 Hz, 1H), 8.21 (d, J = 6.9 Hz, 1H), 8.12 (d, J = 8.3 Hz, 2H), 7.87 (br s, 1H), 7.81 (br s, 1H), 7.71 (br s, 1H), 7.62 (t, J = 7.3 Hz, 1H), 7.51 (t, J = 7.7 Hz, 2H), 7.40 (dd, J = 2.6, 6.5 Hz, 1H), 7.12 (br s, 1H) 7.08 (dd, J = 2.7, 6.8 Hz, 1H, H^b), 5.05 (s, 1H), 4.95 (s, 1H), 4.00 (q, J = 5.9 Hz, 4H), 3.84 (t, J = 6.0 Hz, 2H), 3.70 (t, J = 6.0 Hz, 2H).; HPLC-MS (ESI): Purity = 95%, t_R = 2.48 min, (*m/z*) $[M+H]^+$ = 687.0, calculated mass $[M+H]^+$ = 687.0.

1.2.5.6. (*N*-Benzoyl-*N*',*N*'-di(2-hydroxyethylthioureato)-*S*,*O*)(4,4'-diamino-2,2'-bipyridyl)platinum(II) Chloride (C6)

Compound **2a** (67.9 mg, 0.253 mmol, 1.1 eq.) and triethylamine (35 µL, 253 mmol, 1.1 eq.) in acetone were added to compound **3f** (103 mg, 0.230 mmol, 1 eq.) Compound **C6** was purified using reverse phase column chromatography and isolated as a brown solid (28.8 mg, 14%); ^{1}H NMR (300 MHz, CD_3CN-d_3/D_2O) δ 7.66 (d, J = 6.1 Hz, 1H), 7.60 (d, J = 7.3 Hz, 2H), 7.50 (t, J = 7.1 Hz, 1H), 7.27 (t, J = 7.5 Hz, 2H), 7.11 (d, J = 6.7 Hz, 1H), 6.50 (br s, 1H), 7.45 (br s, 1H), 6.33 (d, J = 6.4 Hz, 1H), 6.14 (d, J = 6.8 Hz, 1H), 3.76 (t, J = 6.3 Hz, 4H), 3.64 (br s, 4H).; HPLC-MS (ESI): Purity = 97%, t_R = 0.87 min, (*m/z*) $[M-Cl]^+$ = 648.1, calculated mass $[M-Cl]^+$ = 648.6.

1.2.5.7. (*N*-Benzoyl-*N*',*N*'-di(2-hydroxyethylthioureato)-*S*,*O*)(4,4'-dichloro-2,2'-bipyridyl)platinum(II) Chloride (C7)

Compound **2a** (61.7 mg, 0.229 mmol, 1.1 eq.) and triethylamine (32 µL, 0.229 mmol, 1.1 eq.) in acetone were added to compound **3g** (102 mg, 0.209 mmol, 1 eq.). Compound **C7** was purified using reverse phase column chromatography and isolated as a yellow solid (133 mg, 88%); ^{1}H NMR (600 MHz, MeOD-d_4) δ 9.09 (d, J = 6.2 Hz, 1H), 8.79 (d, J = 2.1 Hz, 1H), 8.75 – 8.68 (m, 2H), 8.17 (d, J = 6.3 Hz, 1H), 8.15 (d, J = 7.7 Hz, 2H), 7.82 (d, J = 6.3 Hz, 1H), 7.65 (t, J = 7.3 Hz, 1H), 7.53 (t, J = 7.6 Hz, 2H), 4.18 (t, J = 5.9 Hz, 2H), 4.14 (t, J =5.8 Hz, 2H), 4.06 (t, J = 5.7 Hz, 2H), 3.92 (t, J = 5.8 Hz, 2H). ^{13}C NMR (151 MHz, DMSO-d_6) δ 169.28, 168.57, 168.30, 166.85, 158.96, 156.48, 149.35, 145.52, 135.32, 132.95, 129.64 (2C), 129.25(2C), 115.29, 115.03, 112.99, 112.01, 59.20, 58.43, 56.78, 55.36.; HPLC-MS (ESI): Purity = 99%, t_R = 2.39 min, (*m/z*) $[M-Cl]^+$ = 687.1, calculated mass $[M-Cl]^+$ = 687.5.

1.2.5.8. (*N*-Benzoyl-*N*',*N*'-di(2-hydroxyethylthioureato)-*S*,*O*)(bis(trifluoromethyl)-2,2'-bipyridyl)platinum(II) Chloride (C8)

Compound **2a** (98.4 mg, 0.334 mmol, 1.1 eq.) and triethylamine (51 µL, 0.367 mmol, 1.1 eq.) in acetone were added to compound **3h** (186 mg, 0.334 mmol, 1 eq.). Compound **C8** was purified using reverse phase column chromatography and isolated as a yellow solid (112 mg, 56%); ^{1}H NMR (600 MHz, MeOD-d_4) δ 9.49 (d, *J* = 5.9 Hz, 1H), 9.27 (d, *J* = 6.0 Hz, 1H), 9.18 (d, *J* = 6.0 Hz, 1H), 9.14 (d, *J* = 5.9 Hz, 1H), 8.47 (dd, *J* = 2.1, 6.1 Hz, 1H), 8.22 (d, *J* = 6.1 Hz, 1H), 8.21 (d, *J*= 6.0 Hz, 1H), 8.09 (dd, *J* = 2.0, 6.1 Hz, 1H), 7.63 (t, *J* = 7.2 Hz, 1H), 7.53 (t, *J* = 7.6 Hz, 2H), 4.22 (t, *J* = 5.8 Hz, 2H), 4.17 (t, *J* = 5.8 Hz, 2H), 4.06 (t, *J* = 5.7 Hz, 2H), 3.91 (t, *J* = 5.8 Hz, 2H).; HPLC-MS (ESI): Purity = 96%, t_R = 0.95 min, (*m/z*) $[M-Cl]^+$ = 754.1, calculated mass $[M-Cl]^+$ = 754.6.

1.2.5.9. (*N*-Benzoyl-*N*',*N*'-di(2-hydroxyethylthioureato)-*S*,*O*)(*N*4,*N*4'-dimethyl-2,2'-bipyridyl)-4,4'-dicarboxamideplatinum(II) Chloride (C9)

Compound **1a** (32.6 mg, 0.122 mmol, 1.1 eq.) and triethylamine (17 µL, 0.122 mmol, 1.1 eq.) in MeCN were added to compound **3i** (59.3 mg, 0.111 mmol, 1 eq.). Compound **C9** was washed with copious amounts of acetone and isolated as a yellow solid (82.6 mg, 77%); ^{1}H NMR (400 MHz, DMSO-d_6) δ 9.27 – 9.21 (m, 3H), 9.15 – 9.11 (m, 2H), 8.81 (d, *J* = 6.1 Hz, 1H), 8.48 (d, *J* = 5.9 Hz, 1H), 8.15 (d, *J* = 7.7 Hz, 1H), 8.10 (d, *J* = 5.9 Hz, 1H), 7.69 (t, *J* = 7.3 Hz, 1H), 7.54 (t, *J* = 7.6 Hz, 1H), 5.18 (t, *J* = 5.4 Hz, 1H), 5.04 (t, *J* = 5.3 Hz, 1H), 4.11 – 4.01 (m, 4H), 3.92 (q, *J* = 5.7 Hz, 2H), 3.76 (q, *J* = 5.8 Hz, 2H), 2.92 (d, *J* = 4.4 Hz, 3H), 2.88 (d, *J* = 4.4 Hz, 3H).; HPLC-MS (ESI): Purity = 98%, t_R = 0.81 min, (*m/z*) $[M-Cl]^+$ = 732.1, calculated mass $[M-Cl]^+$ = 732.7.

1.2.5.10. (*N*-Benzoyl-*N*',*N*'-di(2-hydroxyethylthioureato)-*S*,*O*)(*N*4,*N*4'-dimethyl-2,2'-bipyridyl)-4,4'-dicarboxylateplatinum(II) Chloride (C10)

Compound **1b** (98.9 mg, 0.369 mmol, 1.1 eq.) and triethylamine (51.3 µL, 0. 369 mmol, 1.1 eq.) in acetone was added to compound **3g** (180 mg, 0.335 mmol, 1 eq.). Compound 4j was purified using reverse column chromatography and preparative HPLC. Yield: 2.2 mg (3 %); HPLC-MS (ESI): Purity = 95%, t_R = 0.9 min, (*m/z*) $[M-Cl]^+$ = 734.1, calculated mass $[M-Cl]^+$ = 734.7.

1.2.5.11. *N*-(4-methyl-Benzoyl-*N*',*N*'-di(2-hydroxyethylthioureato)-*S*,*O*)(4,4'-di-*tert*-butyl-2,2'-bipyridyl)platinum(II) chloride (C11)

Compound **2b** (70.6 mg, 0.250 mmol, 1.1 eq.) and triethylamine (35 µL, 0.250 mmol, 1.1 eq.) in acetone were added to a solution of compound **3a** (0.121 g, 0.227 mmol 1 eq.) in acetone. Compound **C11** was isolated as a yellow solid (146 mg, 96 %); ^{1}H NMR (300 MHz, CD_3OD-*d_3*) δ 8.83 (d, *J* = 6.1 Hz, 1H), 8.50 (d, *J* = 2.0 Hz, 1H), 8.44 (d, *J* = 6.3 Hz, 1H), 8.40 (d, *J* = 2.1 Hz, 1H), 7.92 (dd, *J* = 2.0, 6.1 Hz, 1H), 7.87 (d, *J* = 8.2 Hz, 2H), 7.57 (dd, *J* = 2.2, 6.3 Hz, 1H), 7.20 (d, *J* = 8.0 Hz, 2H), 4.02 (t, *J* = 4.7 Hz, 2H), 3.95 (q, *J* =, 5.7 Hz, 4H), 3.76 (t, *J* = 5.7 Hz, 2H), 2.33 (s, 3H), 1.44 (s, 9H), 1.37 (s, 9H).; HPLC-MS (ESI): Purity = 97%, t_R = 1.12 min, (*m/z*) $[M-Cl]^+$ = 744.2, calculated mass $[M-Cl]^+$ = 744.8.

1.2.5.12. *N*-(4-methoxy-benzoyl-*N*',*N*'-di(2-hydroxyethylthioureato)-*S*,*O*)(4,4'-di-*tert*-butyl-2,2'-bipyridyl)platinum(II) Chloride (C12)

Compound **2c** (63.6 mg, 0.213 mmol, 1.1 eq.) and triethylamine (30 µL, 0.213 mmol, 1.1 eq.) in acetone were added to a solution of compound **3a** (103 mg, 0.193 mmol 1 eq.) in acetone. Compound **C12** was isolated as a yellow solid (142 mg, 92 %); ^{1}H NMR (300 MHz, DMSO-*d_6*) δ 8.85 (d, *J* = 6.1 Hz, 1H), 8.78 (d, *J* = 2.0 Hz, 1H), 8.67 (d, *J* = 2.1 Hz, 1H), 8.48 (d, *J* = 6.3 Hz, 1H), 8.09 – 8.03 (m, 3H), 7.72 (dd, *J* = 2.1, 6.3 Hz, 1H), 7.04 (d, *J* = 8.9 Hz, 2H), 5.25 (t, *J* = 5.2 Hz, 1H), 5.07 (t, *J* = 5.3 Hz, 1H), 4.01 (t, *J* = 5.8 Hz, 2H), 3.95 (t, *J* = 6.0 Hz, 2H), 3.91 – 3.85 (m, 5H), 3.69 (q, *J* = 5.6 Hz, 2H), 1.48 (s, 9H), 1.41 (s, 9H).; HPLC-MS (ESI): Purity = 98%, t_R = 1.12 min, (*m/z*) $[M-Cl]^+$ = 760.2, calculated mass $[M-Cl]^+$ = 760.8.

1.2.5.13. *N*-(4-chloro-Benzoyl-*N*',*N*'-di(2-hydroxyethylthioureato)-*S*,*O*)(4,4'-di-*tert*-butyl-2,2'-bipyridyl)platinum(II) Chloride (C13)

Compound **2d** (79.9 mg, 0.264 mmol, 1.1 eq.) and triethylamine (37 µL, 0.264 mmol, 1.1 eq.) in acetone were added to a solution of compound **3a** (0.128 g, 0.239 mmol, 1 eq.) in acetone. Compound **C13** was isolated as a yellow solid (139 mg, 72 %); ^{1}H NMR (300 MHz, DMSO-*d_6*) δ 8.83 (d, *J* = 6.1 Hz, 1H), 8.80 (d, *J* = 2.0 Hz, 1H), 8.68 (d, *J* = 2.1 Hz, 1H), 8.50 (d, *J* = 6.3 Hz, 1H), 8.10 – 8.05 (m, 3H), 7.73 (dd, *J* = 2.1, 6.3 Hz, 1H), 7.54 (d, *J* = 8.7 Hz, 2H), 5.28 (t, *J* = 5.2 Hz, 1H), 5.10 (t, *J* = 5.3 Hz, 1H), 4.04 – 3.89 (m, 6H), 3.70 (d, *J* = 5.5 Hz, 2H), 1.49 (s, 9H), 1.41 (s, 9H).; HPLC-MS (ESI): Purity = 99%, t_R = 1.13 min, (*m/z*) $[M-Cl]^+$ = 765.2, calculated $[M-Cl]^+$ = 765.2.

1.2.5.14.(*N*-Benzoyl-*N*',*N*'-di(2-hydroxyethylthioureato)-*S*,*O*)(5,5'-dimethyl-2,2'-bipyridyl)platinum(II) Chloride (C14)

Compound **2a** (78.4 mg, 0.292 mmol, 1.1 eq.) and triethylamine (41 µL, 0.292 mmol, 1.1 eq.) in dry DMF was added to compound **3k** (119 mg, 0.266 mmol, 1 eq.) in dry DMF. Compound **C14** was isolated as a yellow solid (148 mg, 77%); ^{1}H NMR (300 MHz, D_2O/CD_3CN-*d_3*) δ 8.25 – 8.14 (m, 4H), 8.13 – 8.03 (m, 5H), 7.86 (t, *J* = 7.7 Hz, 2H), 4.32-4.37 (m, 4H), 4.19 (s, 4H), 2.59 (s, 3H), 2.56 (s, 3H).; HPLC-MS (ESI): Purity = 98%, t_R = 2.33 min, (*m/z*) $[M-Cl]^+$ = 646.1, calculated mass $[M-Cl]^+$ = 646.1.

1.2.5.15.(*N*-Benzoyl-*N*',*N*'-di(2-hydroxyethylthioureato)-*S*,*O*)(6,6'-dimethyl-2,2'-bipyridyl)platinum(II) Chloride (C15)

Compound **2a** (78.4 mg, 0.292 mmol, 1.1 eq.) and triethylamine (41 µL, 0.292 mmol, 1.1 eq.) in dry DMF were added to compound **3l** (119 mg, 0.266 mmol, 1 eq.) in dry DMF. Compound **C14** was isolated as a yellow solid (148 mg, 77%); ^{1}H NMR (300 MHz, DMSO-*d_6*) δ 8.01-8.09 (m, 5H), 7.63 – 7.43 (m, 5H), 6.92 (d, *J* = 8.5 Hz, 1H), 5.05 (s, 1H), 4.95 (s, 1H), 4.15 – 3.92 (m, 4H), (t, *J* = 5.0 Hz, 2H), 3.73 (t, *J* = 5.4 Hz, 2H), 3.02 (s, 3H), 2.40 (s, 1H). ^{13}C-NMR (151 MHz, DMSO-*d_6*) δ 168.31, 167.53, 162.71, 162.37, 160.70, 152.41, 139.14, 138.12, 131.53, (2C) 128.93, 128.46 (2C), 126.57, 125.48, 122.89, 118.10, 58.93 (2C), 58.20 (2C), 55.38, 54.47, 23.37. HPLC-MS: Purity = 98%, t_R = 2.69 min, (*m/z*) $[M-Cl]^+$ = 646.1, calculated mass $[M-Cl]^+$ = 646.1.

1.2.5.16.(*N*-Benzoyl-*N*',*N*'-di(2-hydroxyethylthioureato)-*S*,*O*)(4,4'-di-*tert*-butyl-2,2'-bipyridyl)platinum(II) hexafluorophosphate (C16)

NH_4PF_6 (6.78 mg, 0.042 mmol, 1.1 eq.) was added to Compound **C1** (29.1 mg, 0.038 mmol, 1 eq.) in dry DCM:EtOH (1:1). The reaction mixture was allowed to stir under argon at room temperature (27 °C) for 1 hour. The resulting precipitate was collected *via* suction filtration. Compound **C16** was isolated as a yellow solid (29.4 mg, 89%);^{1}H NMR (300 MHz, DMSO-*d_6*) δ 8.88 (d, *J* = 6.1 Hz, 1H), 8.77 (d, *J* = 2.1 Hz, 1H), 8.66 (d, *J* = 2.4 Hz, 1H), 8.51 (d, *J* = 6.3 Hz, 1H), 8.12 (d, *J* = 7.1 Hz, 2H), 8.07 (dd, *J* = 6.0, 2.1 Hz, 1H), 7.68-7.75 (m, 3H), 7.55 (t, *J* = 7.7 Hz, 2H), 5.19 (t, *J* = 6.0 Hz, 1H), 5.05 (t, *J*= 6.0 Hz, 1H), 4.09 – 3.97 (m, 4H), 3.91 (t, *J* = 6.0 Hz, 2H), 3.72 (t, *J* = 5.7 Hz, 2H). HPLC-MS (ESI): Purity = 95%, t_R = 1.074 min, (*m/z*) $[M-PF_6]^+$ = 730.1, calculated mass $[M-PF_6]^+$ = 730.8. IR (ATR, cm^{-1}): 3601 (broad, weak,

OH), 2960 (sharp, weak, N=C=S), 2874 (sharp, weak, N=C=S), 1619 (sharp, weak, C=N), 1496 (sharp, strong, C=C), 1411 (sharp, strong, C-H), 833 (sharp, strong, PF_6).

1.2.5.17. (*N*-Benzoyl-*N*',*N*'-di(2-hydroxyethylthioureato)-*S*,*O*)(4,4'-di-*tert*-butyl-2,2'-bipyridyl)platinum(II) nitrate (C17)

$AgNO_3$ (7.80 mg, 0.046 mmol, 1.1 eq.) was added to Compound **C1** (32.1 mg, 0.042 mmol, 1 eq.) in dry MeOH (2 mL). The reaction mixture was stirred in the dark under argon at room temperature (25 °C) for 3 hours. The resulting precipitate was collected *via* suction filtration. A crude mixture of **C17** was collected as a yellow solid (Yield: 5.00 mg, 15%); ^{1}H NMR (300 MHz, DMSO-d_6) δ 8.92 (d, J = 5.9 Hz, 1H), 8.79 (br s, 1H), 8.69 (br s, 1H), 8.55 (d, J = 6.2 Hz, 1H), 8.14 (d, J = 8.4 Hz, 3H), 7.81 – 7.65 (m, 2H), 7.60 – 7.50 (d, J = 6.2 Hz, 2H), 5.17 (t, J = 5.7 Hz, 1H), 5.03 (t, J = 5.6 Hz, 1H), 4.11 – 3.96 (m, 4H), 3.91 (t, J = 6.0 Hz, 2H), 3.73 (d, J = 5.9 Hz, 2H). ^{13}C-NMR (151 MHz, DMSO-d_6) δ 168.85, 166.10, 165.78, 157.02, 154.10, 148.09, 143.89, 134.61, 132.73, 129.23 (2C), 128.82 (2C), 124.93, 122.49, 121.93, 58.77 (2C), 58.01 (2C), 56.43, 55.02, 36.19, 36.00, 29.86 (6 C). HPLC-MS (ESI): Purity = 97%, t_R = 1.074 min, (*m/z*) $[M-NO_3]^+$ = 730.1, calculated mass $[M-NO_3]^+$ = 730.1; IR (ATR, cm^{-1}): 3392 (broad, weak, OH), 2965 (sharp, weak, N=C=S), 2872 (sharp, weak, N=C=S), 1621 (sharp, weak, C=N), 1494 (sharp, strong, C=C), 1808 (sharp, strong, C-H), 1396 (broad, strong, NO_3).

1.3. Cultivation of *Plasmodium falciparum* parasites

All work on *Plasmodium* parasites was approved by the University of Pretoria Research Ethics Committee (gee asb codes – SARChI en CoP). Asexual stages of the chloroquine-sensitive wild type (NF54) and chloroquine-resistant (K1) strains of *Plasmodium falciparum (Pf)* were maintained in complete culture media [RPMI 1640 (Sigma-Aldrich) supplemented with 25 mM HEPES, 20 mM D-glucose, 200 μM hypoxanthine, 0.2% (w/v) sodium bicarbonate, 24 μg/mL gentamicin, and 0.5% (w/v) AlbuMAX II)] at 2-5% hematocrit. Cultures were kept under gas conditions containing 90% N_2, 5% O_2, and 5% CO_2 and incubated at 37 °C for the duration of the culturing period. Synchronous cultures were obtained with 5% D-sorbitol (w/v). The morphology and development of the parasites were monitored using Giemsa-stained smears microscopy at 1000x magnification.

1.4. *In vitro* activity against ABS parasites

Inhibition of ABS parasite proliferation was determined with SYBR Green I fluorescence[1] on synchronous *Pf*-NF54 and *Pf*-K1 (1% parasitemia, 1% hematocrit, >90% ring-stage parasites) were exposed to the respective drugs at a maximum starting concentration of 5 μM.

Chloroquine (CQ) was used as the positive control. The parasites were incubated under drug pressure for 96 h at 37 °C and the same hypoxic conditions described above. After 96 h incubation, parasite survival was determined by adding equal volumes of parasite suspension (100 µL) and SYBR Green I lysis buffer (0.2% µL/mL of 10 000x SYBR Green I, 20 mM Tris-HCl, pH 7.5, 5 mM EDTA, 0.008% saponin (w/v) and 0.08% Triton x-100). This was incubated for 1 hour at room temperature in the dark. Fluorescence was determined using a GloMax®-Multi+ Detection System at 485/538 nm wavelengths. Three independent biological replicates each containing three technical repeats were performed to determine the dose-response curves from which the IC_{50} (minimum concentration required to inhibit 50% cell proliferation) values are reported.

1.5. *In vitro* activity against gametocyte stages

*Pf*NF54 gametocytes were prepared and isolated according to the method reported by Reader *et al.*[2,3] Methylene Blue (MB) was used as a positive control. The *in vitro* gametocyte activity of the synthesized complexes was determined using a luciferase reporter line-based assay. NF54-*Pf*S16-GFP-Luc parasites were used for the stage-specific assessment of gametocytocidal activity. Early-stage gametocyte (stage I-III) experiments were performed on day 5 (representing >90% of early-stage and late-stage gametocyte (stages IV/V) experiments were performed on day 10. In each experiment, assays were set up with the use of 2-3% gametocytemia, 1.5% hematocrit was used for each assay, and the gametocytes were incubated at 37 °C for 48 h under drug pressure in a gas chamber (90% N_2, 5% O_2, and 5% CO_2). Luciferase activity was determined in 30 µL parasite lysates by adding luciferin substrate (30 µL) (Promega Luciferase Assay System) at room temperature. The resulting bioluminescence at an integration constant of 10 s was detected using the GloMax®-Multi+ Detection System with Instinct® Software.

1.6. *In vitro* cytotoxicity assay

A 3-(4,5-dimethylthiazol-2-yl)-2,5-diphenyltetrazoliumbromide (MTT) assay was used to screen the metal complexes against the mammalian Chinese hamster ovarian (CHO) cell line. A stock solution of each compound (2 mg/mL) in DMSO was prepared and kept at -20 °C until needed. Emetine was used as a reference drug. A starting concentration of 100 µg/mL was used, and serially diluted ten-fold using a complete medium to give the lowest concentration of 0.001 µg/mL (6 dilutions). The plates containing the CHO cells under drug pressure were incubated for 48 h hours. Plates containing the drug and cell suspension were developed by adding 25 µL of sterile MTT (Thermo Fisher Scientific) (25 µL) to each well, followed by 4 h

of incubation at room temperature in the dark. The plates were then centrifuged, and the supernatant was removed via aspiration. Sample absorbance was read at a wavelength of 540 nm. The full dose-response curves were plotted using a non-linear dose-response curve fitting analysis via GraphPad Prism v.4.0 software. The IC_{50} values were determined for each compound were determined from the dose-response curves.

1.7. Kinetic solubility by HPLC (pH 6.5)

Solubility was measured at pH 6.5 using an adapted miniaturized shake-flask method, in 96-well plate format.[4,5] Briefly, 4 µL of a 10 mM stock in DMSO was added to a 96-well plate and evaporated using a GeneVac system. Phosphate buffer pH 6.5 was then added to the wells and the plate was incubated for 24 h at 25 °C with shaking. At the end of this incubation, the samples were centrifuged at 3500 *g* for 15 min and then transferred to an analysis plate. A calibration curve in DMSO for each sample between 10 – 220 µM was prepared and included in the analysis plate. Analysis was then performed by HPLC-DAD and the solubility of each sample was determined from the corresponding calibration curve. Reserpine (solubility <5 µM) and hydrocortisone (solubility >180 µM) were used as controls and gave data that compared well to historical data in our laboratory.

1.8. Microsomal metabolic stability studies

The metabolic stability assay was performed using a single-point metabolic stability assay.[6] Briefly, the compounds were incubated at 1 µM in human (mixed gender, Xenotech), Rat (male rat IGS, Xenotech), and mouse (male mouse CD1, Xenotech) liver microsomes (0.4 mg/ml) for 30 min at 37 °C. Reactions were quenched by adding ice-cold acetonitrile containing internal standard. The samples were then centrifuged and analyzed by LC-MS/MS for the disappearance of the parent compound. Half-life, clearance, and hepatic excretion ratios were determined using standard equations.[6,7] Propranolol. Midazolam and MMV390048 were used as controls and gave data that compared well to historical data in our laboratory.

1.9. Determination of pharmacokinetic parameters

Triplicates of female Balb/C mice were used for the determination of *in vivo* pharmacokinetic parameters. Compounds were administered either intravenously as a bolus solution (DMA/PEG/PPG/H_2O:5/15/30/50) or orally as a suspension containing 0.5% (w/v) HPMC with 0.2% Tween 80 to female Balb/C mice. Mice were allowed to eat and access water freely.

To determine the PK parameters, blood samples were collected from the tail vein of the mice into heparinized microcentrifugation tubes at distinct sampling times (0.17, 0.5, 1, 3, 5, 7, 9, 24, and 48 hours for intravenous dosing; 0.5, 1, 3, 5, 7, 9, 24 and 48 hours for oral dosing). All samples were stored at -80 °C until extraction.

For the bioanalysis, frozen whole blood was thawed at room temperature and 1-0 µL of each sample was extracted by protein precipitation (using 100 µL of acetonitrile containing 10 ng/ml of the internal standard, MMV394902). The sample was centrifuged. Supernatants of each sample were injected into the LC-MS/MS column for analysis. The analytical limit of quantitation (LOQ) was 2 ng/mL. The pharmacokinetic parameters were calculated using noncompartmental analysis using PK Solutions 2.0 (Summit Research Services, Montrose, CO, USA) and a curve stripping-based method.

1.10. *In vitro* parallel artificial membrane permeability assay (PAMPA) for permeability determination

In preparation for the assay, the required membrane filters were precoated with hexadecane (5%) in hexane and were allowed to dry. The assay was performed with three technical repeats in 96-well MultiScreen filter plates (Millipore, 0.4 µm PCTE membrane). Lucifer yellow was added to the apical wells of the precoated MultiScreen donor plate and served as an integrity marker for the membrane. Phosphate buffer (pH 7.4) was added to the 96-well acceptor plate followed by relevant amounts of the 10 mM stock of the test compound. The donor plate was slotted into an acceptor plate and incubated at room temperature for 4 hours with gentle shaking. Post incubation, aliquots of the samples from the acceptor plates were transferred to the analysis plate, and the matrix was matched with a blank donor buffer. Carbamazepine (0.0236 ng/mL) in acetonitrile was added to all samples as an internal standard. The samples were analyzed using LCMS/MS (Agilent rapid resolution HPLC, AB SCIEX 4500 MS). The permeability (Papp) was calculated by using the normalized (analyte/internal standard) peak areas. Membrane integrity was calculated from the Papp of Lucifer yellow (acceptable values <50nm/s) using a Modulus microplate reader.

1.11. Plasma protein binding (PPB)

To determine the percentage of test compound bound to plasma, ultracentrifugation was used. The test compound (5 µM of a 10 mM DMSO stock) was used to spike pooled human plasma. An aliquot of the spiked solution was removed immediately and quenched with carbamazepine,

0.0236 μg/mL in ice-cold acetonitrile. The aliquot was frozen and served as the total concentration sample to be later referenced against. Samples were pre-incubated for 1 hour at 37 °C. Following pre-incubation, aliquots of the spiked plasma were taken in duplicate and were transferred to ultra-centrifugation tubes. The samples were ultracentrifuged for 4 hours at 37 °C (42000 rpm, Beckman Optima L-80XP). This was followed by analysis using LCMS/MS (Agilent Rapid Resolution HPLC, AB SCIEX 4500 QTRAP MS). The percentage of the test compound bound to plasma protein was calculated by comparison of the analyte: peak ratios of the ultracentrifuged sample to that of the total concentration sample.

1.12. Cyclic voltammetry

For the cyclic voltammetry experiments a solution of the test compound (1 x 10-3) in DMSO (3 mL) is added to the electrolyte solution (0.1 M tetrabutylammonium chloride (TBACl) in DMSO). The electrochemical measurements were obtained using a Basi Epsilon Eclipse Electrochemical Analyzer Potentiostat and the CV graphs were generated using Excel. The working electrode was glassy carbon (GC) with platinum wire as the counter electrode (CE) and Ag/AgCl as the reference.

1.13. Glutathione binding UV-Vis titrations

The binding of glutathione (GSH) to the platinum(II) complexes was monitored by observing the decrease in the metal-to-ligand-charge transfer (MLCT) band via a UV-Vis spectrophotometer.[8] For this, 1–10 μL aliquots of GSH (0.4 M) in a PBS and DMSO (0.01% DMSO, v/v) mixture was added to a cuvette containing the relevant metal complex (50 μM) in the same solvent mixture. After each addition, the solution was stirred and allowed to incubate at room temperature for 3–5 minutes before recording the absorption spectrum between the wavelength range of 250–700 nm. These titrations were performed in triplicate and the average binding constants (log*K*) were reported with the standard error of the mean (SEM). The log*K* values were calculated from the best-fit model (describing a 1:1 association) using **Equation 1**.[9] A_0 is the initial absorbance in the absence of GSH, A_∞ is the final limiting absorbance and [L] is the concentration of GSH.

$$A = \frac{A_0 + A_\infty K[\mathrm{L}]}{1 + K[\mathrm{L}]} \qquad \boldsymbol{Eq. 1}$$

1.14. Hemolysis assay

The hemolysis assay was conducted in a 96-well round-bottom plate. A 10 mM stock of the test compound in DMSO was diluted to 10 µM using complete growth medium. Complete growth medium (100 µL) was added to all wells in columns 1 and columns 3–12. The test compound in a complete growth medium (200 µL) were added in duplicate to well 2 and serially diluted two-fold down the plate (from wells 2–11). Triton X-100 (4%, *v*/*v*) was used as a positive control with chloroquine as a negative control. RBCs in complete growth medium (2% hematocrit) were lysed via three freeze/thaw cycles (-80 °C/37 °C) and were added to all wells in column 1 (100 µL). Intact RBCs in complete growth medium were added to wells 2–12 and the plates were incubated at 37 °C for either 1 hour or 72 hours. After removing the plates from the incubator, each well was mixed thoroughly with a multichannel pipette and spun in a centrifuge at 750 *rcf* for 5 minutes. The supernatant was transferred to a 96-well flat-bottom plate and the absorbance of each well read on a microplate reader (Thermo Scientific Multiskan GO) at a wavelength of 405 nm. Percentage hemolysis was calculated using **Equation 2** and the graphs generated using GraphPad Prism 10.

$$\%\, hemolysis = \frac{(A_s - A_0)}{(A_\infty - A_0)} \times 100 \qquad \boldsymbol{Eq. 2}$$

2. ^{1}H- and ^{13}-C NMR spectra

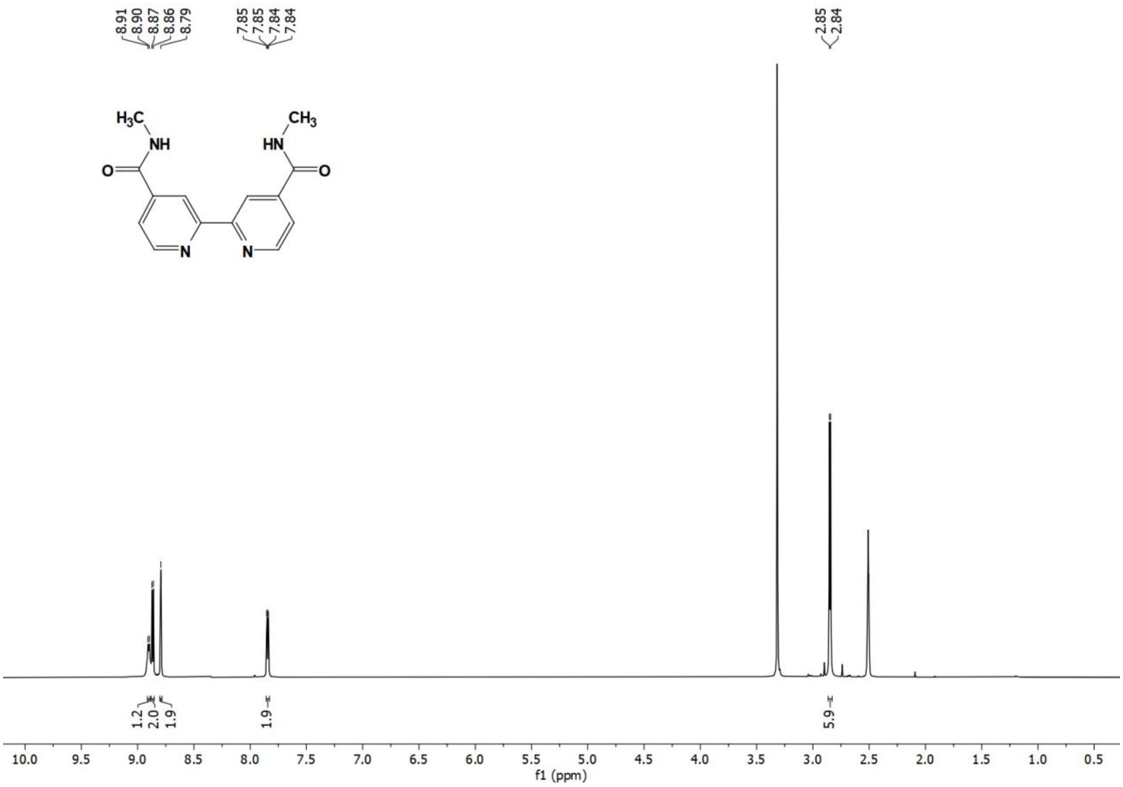

Figure S1. ^{1}H-NMR spectrum of compound **1a** in DMSO-d_6.

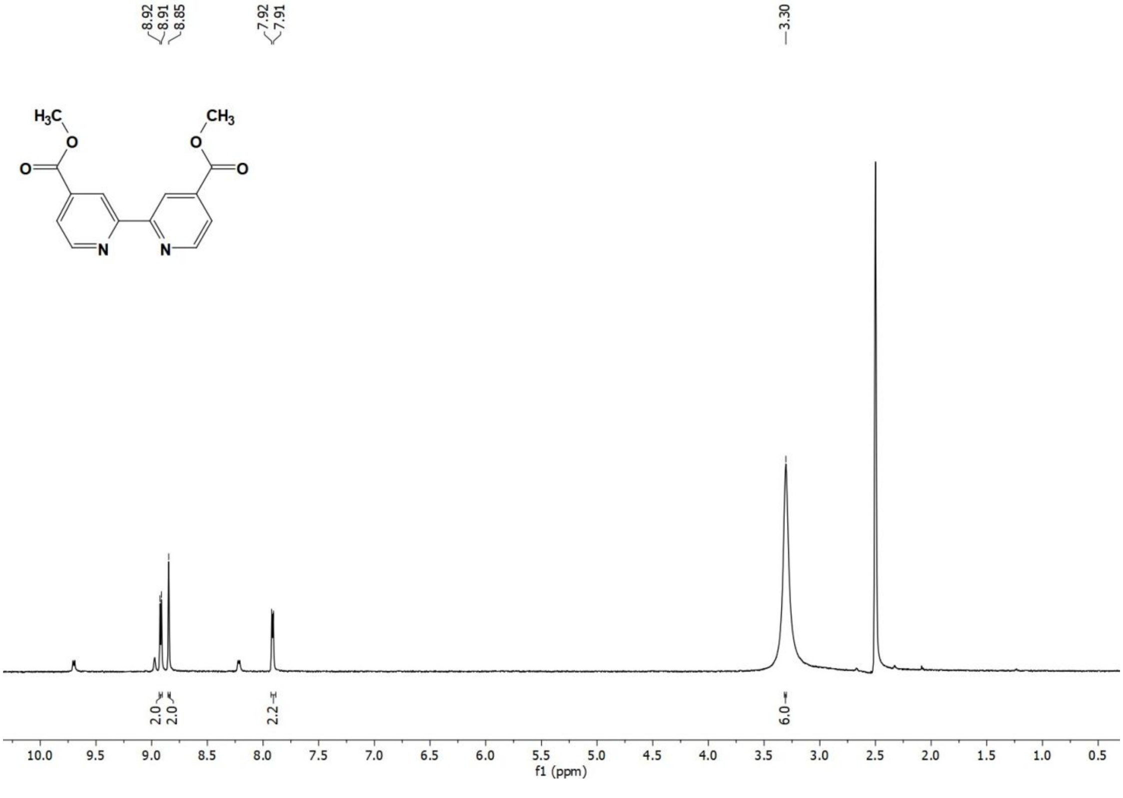

Figure S2. ^{1}H-NMR spectrum of compound **1b** in DMSO-d_6.

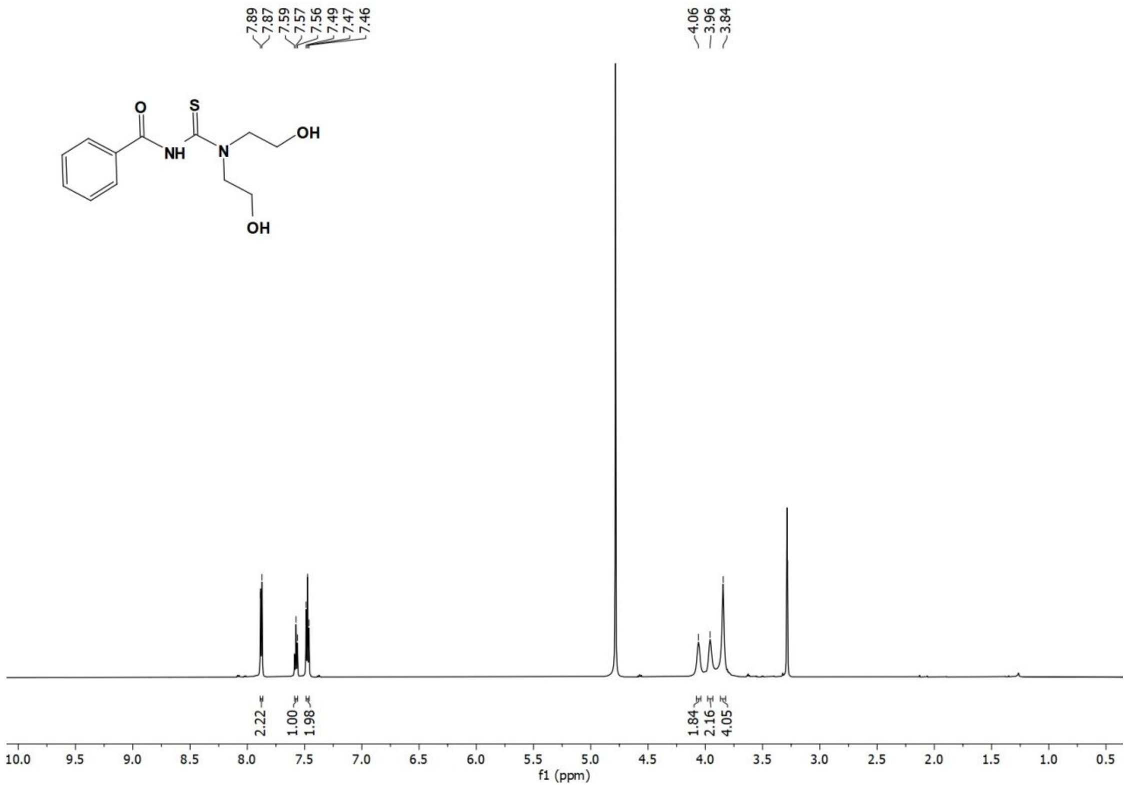

Figure S3. ^{1}H-NMR spectrum of compound **2a** in MeOD-d_4.

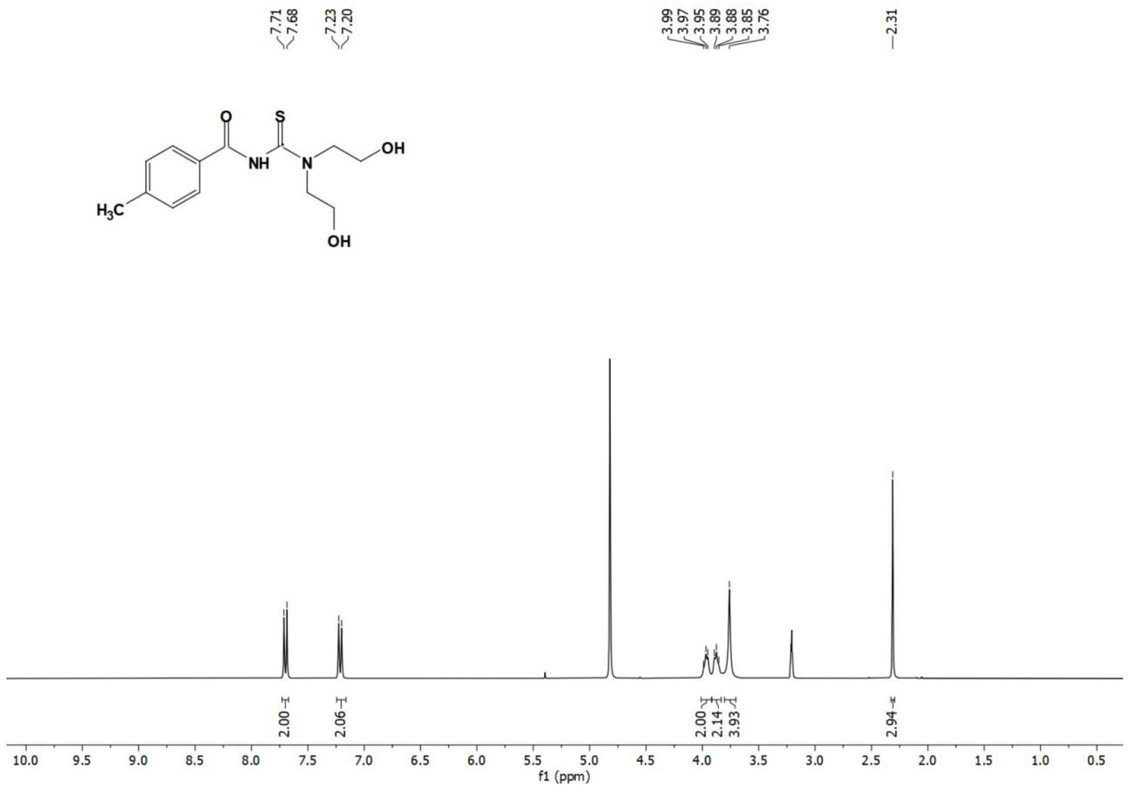

Figure S4. ^{1}H-NMR spectrum of compound **2b** in MeOD-d_4.

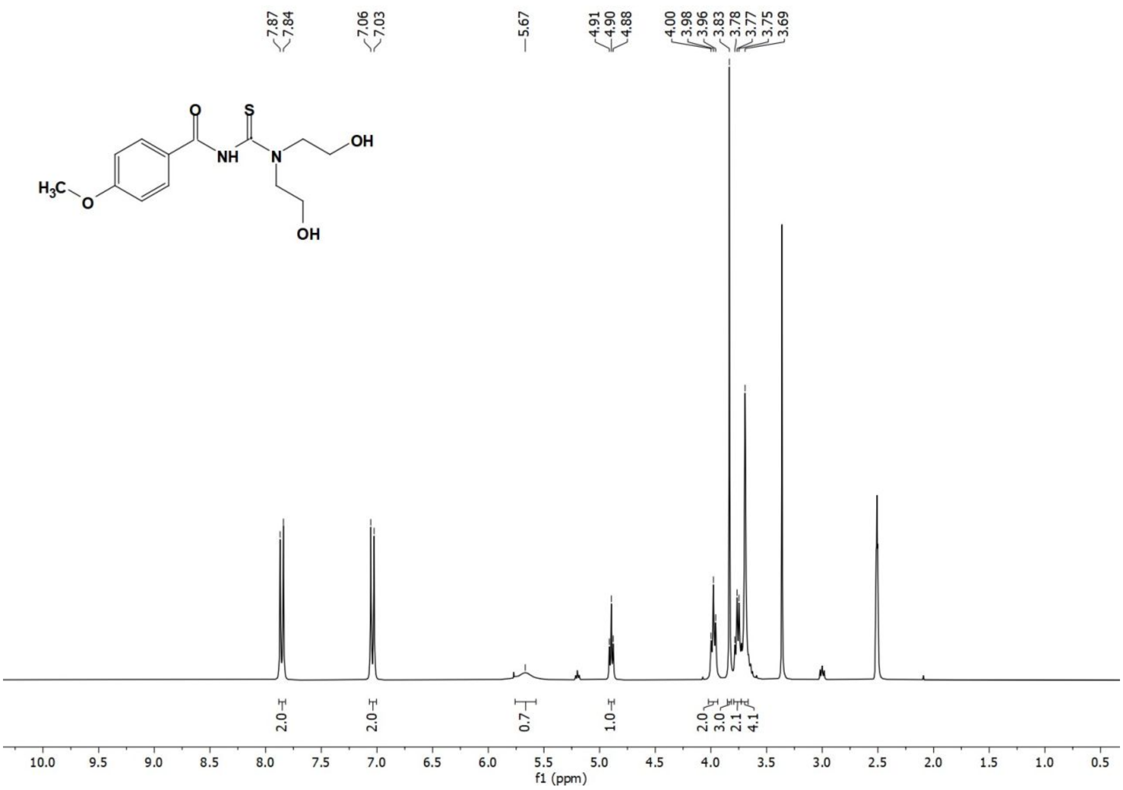

Figure S5. ^{1}H-NMR spectrum of compound **2c** in DMSO-d_6.

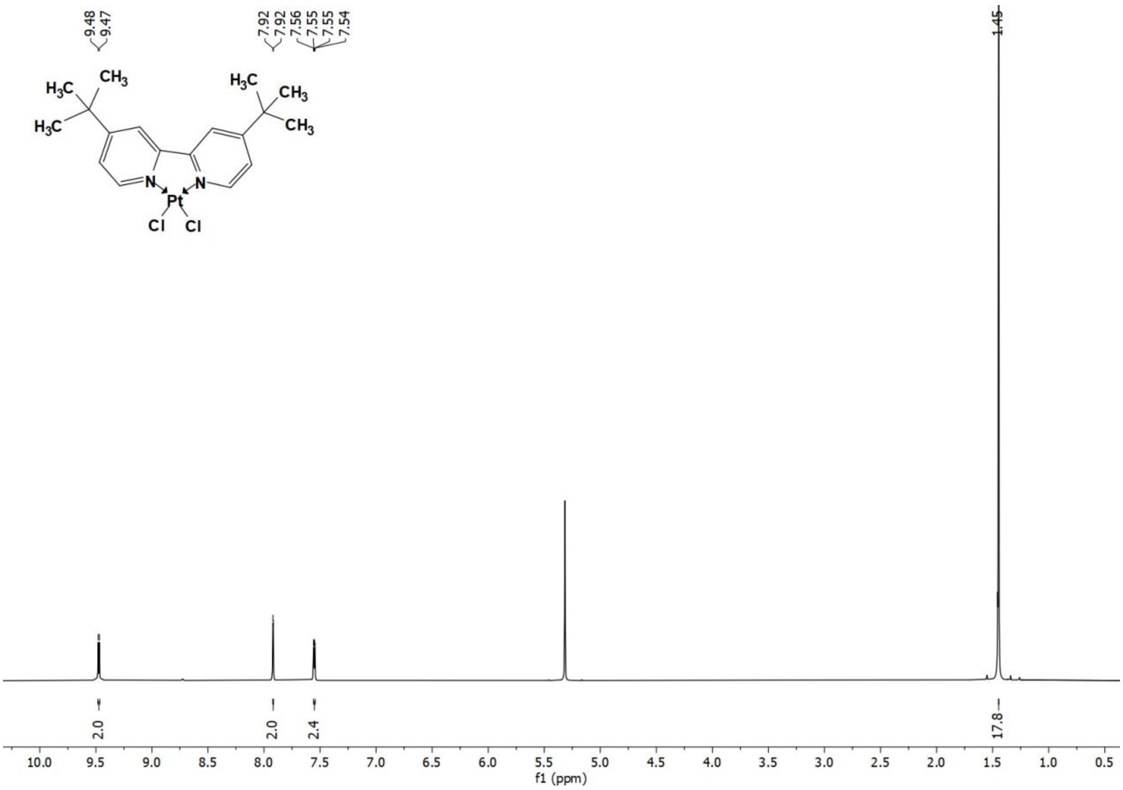

Figure S6. ^{1}H-NMR spectrum of compound **3a** in CD_2Cl_2-d_2.

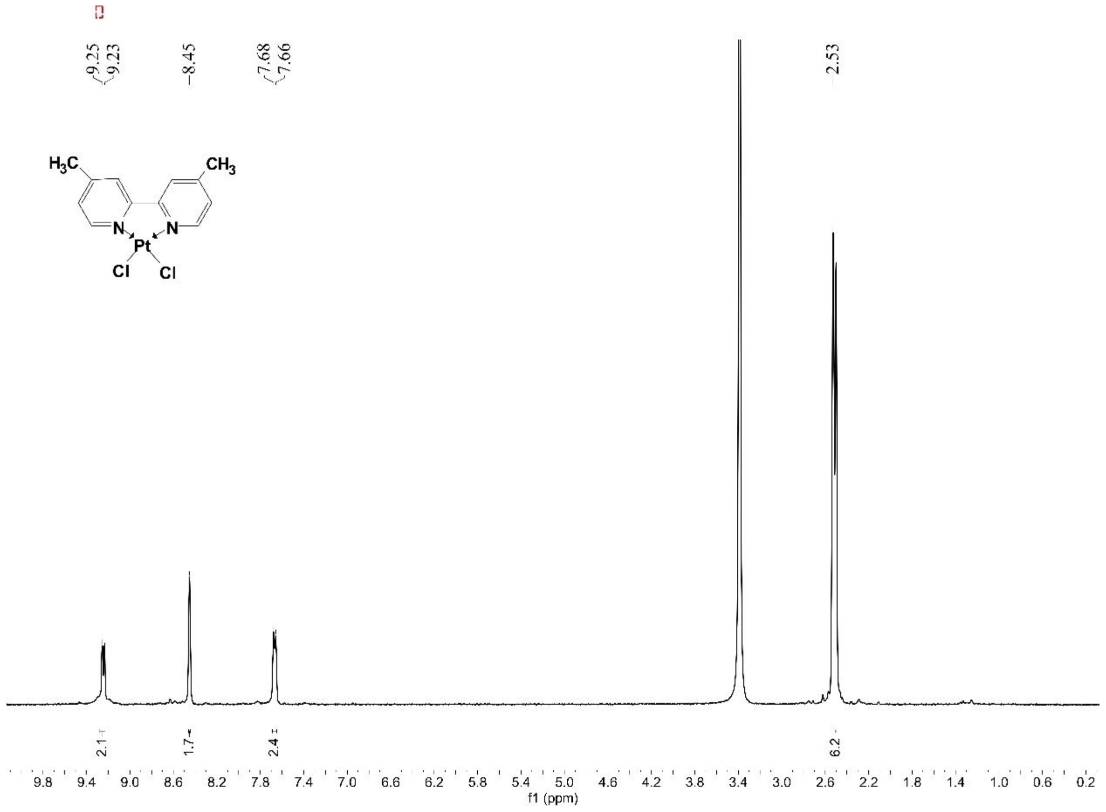

Figure S7. ^{1}H-NMR spectrum of compound **3b** in DMSO-d_6.

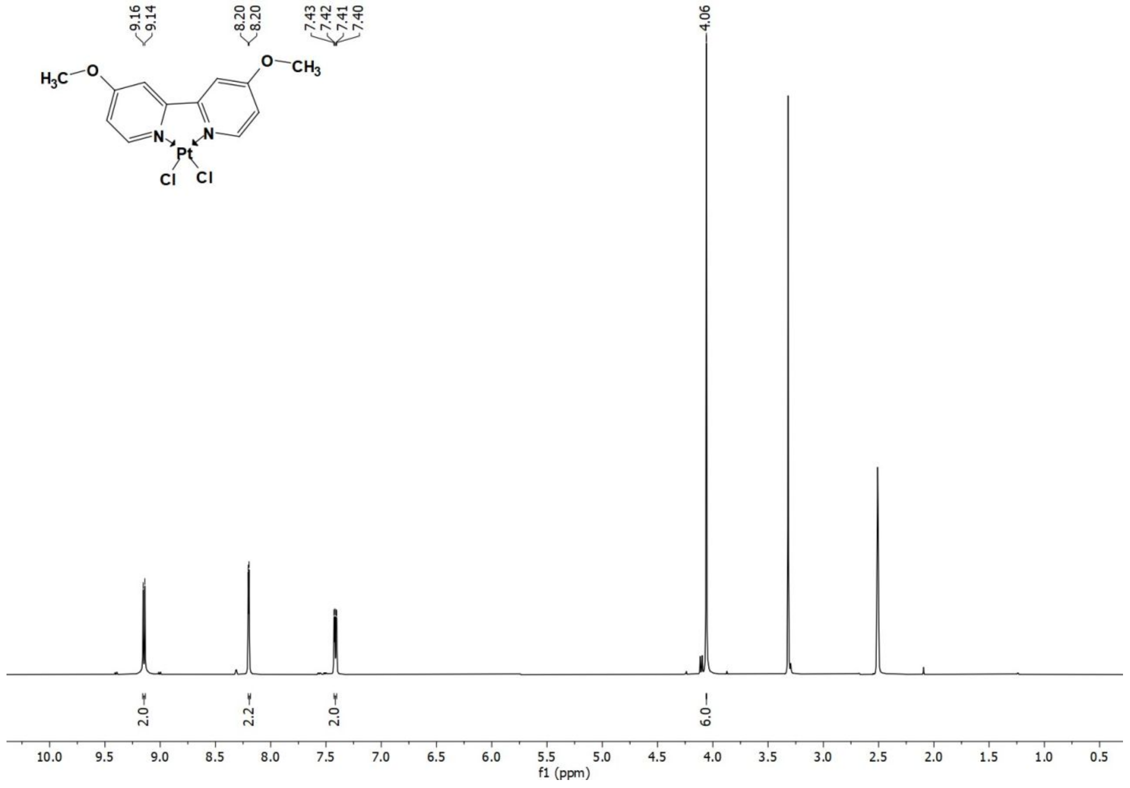

Figure S8. ^{1}H-NMR spectrum of compound **3d** in DMSO-d_6.

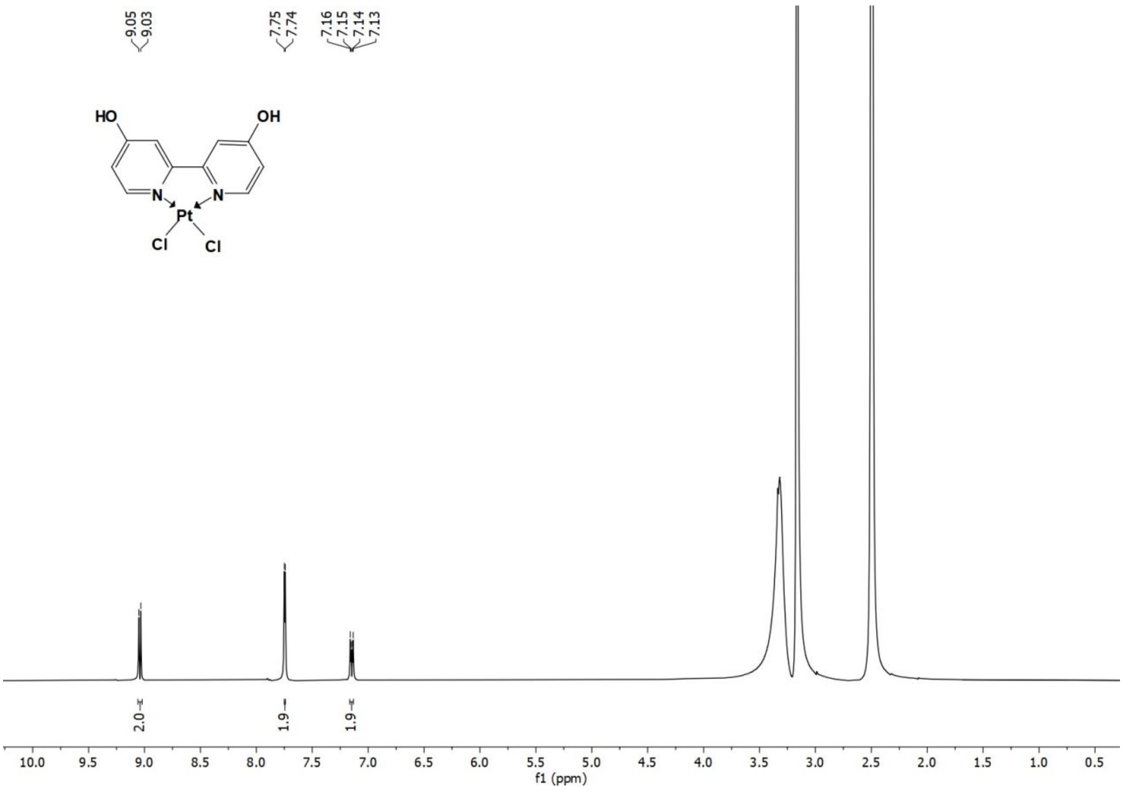

Figure S9. ^{1}H-NMR spectrum of compound **3e** in DMSO-d_6.

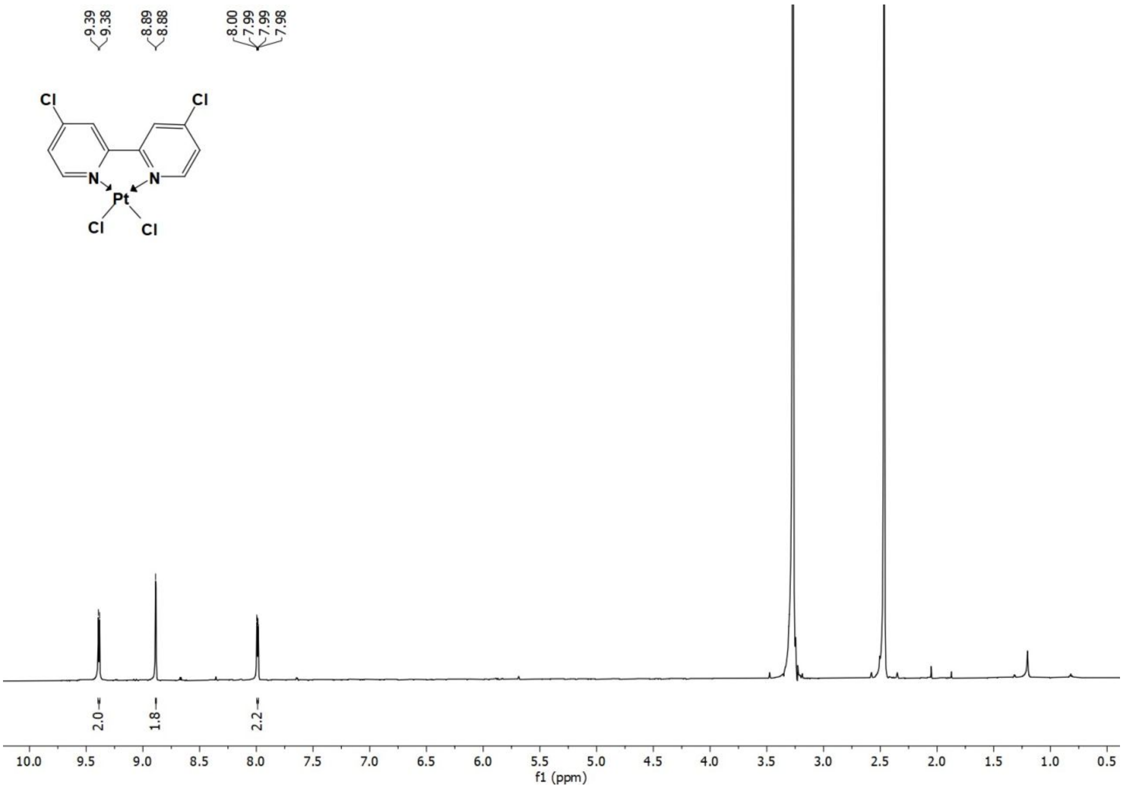

Figure S10. ^{1}H-NMR spectrum of compound **3g** in DMSO-d_6.

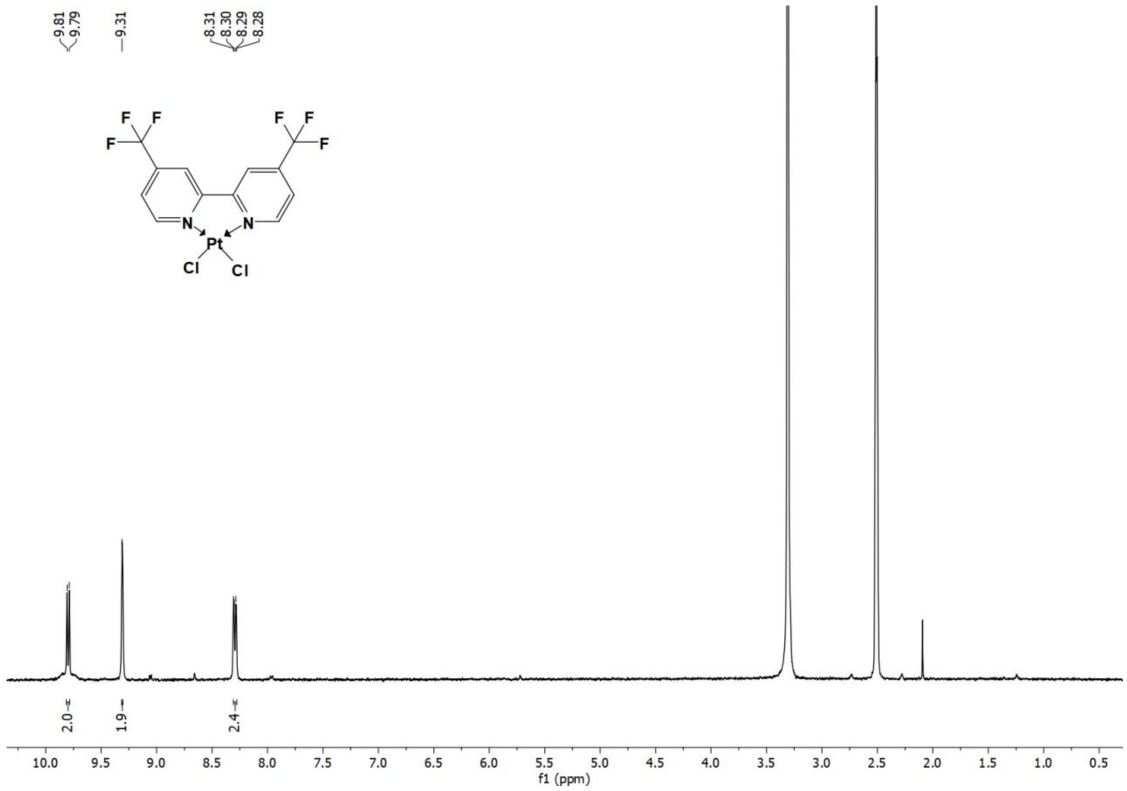

Figure S11. ^{1}H-NMR spectrum of compound **3h** in DMSO-d_6.

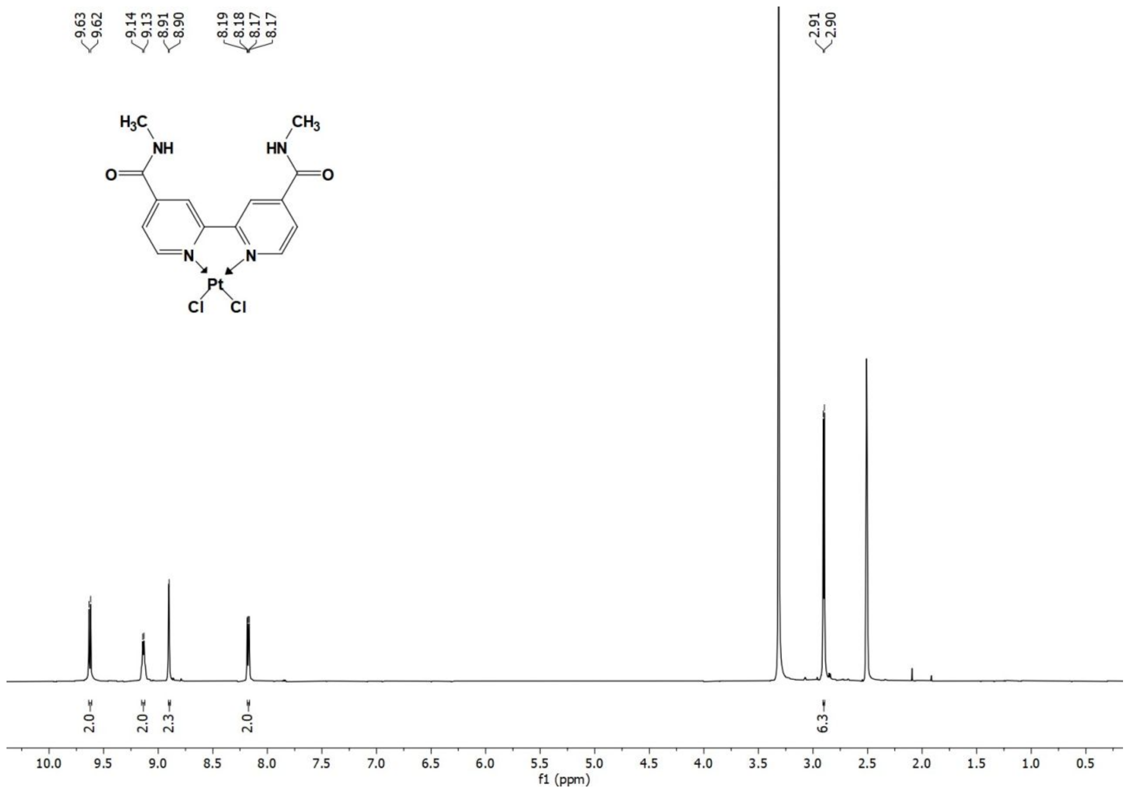

Figure S12. ^{1}H-NMR spectrum of compound **3i** in DMSO-d_6.

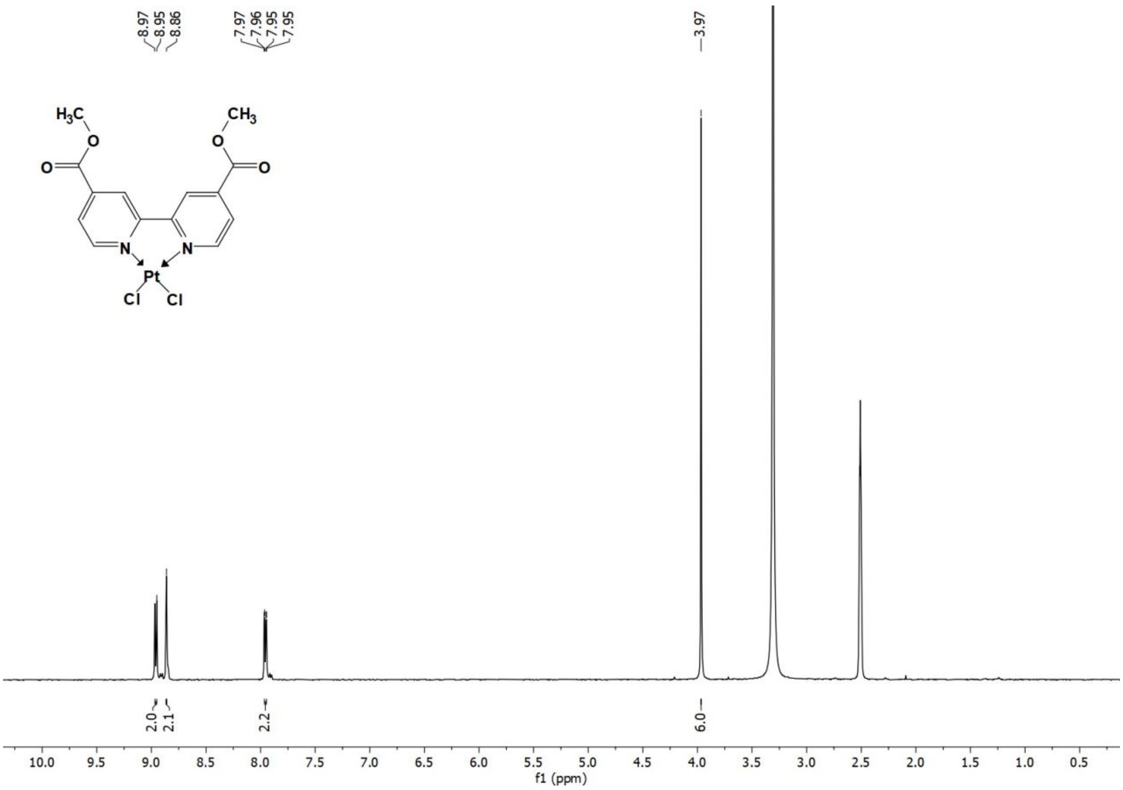

Figure S13. ^{1}H-NMR spectrum of compound **3j** in DMSO-d_6.

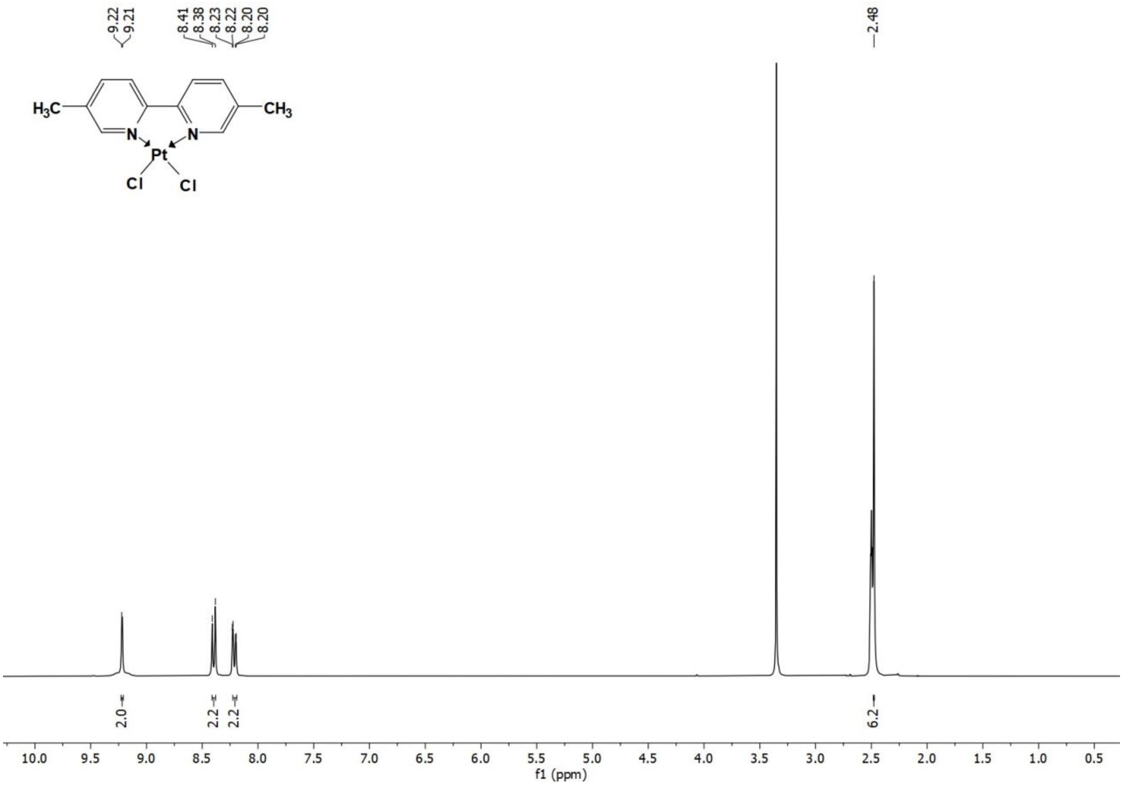

Figure S14. ^{1}H-NMR spectrum of compound **3k** in DMSO-d_6.

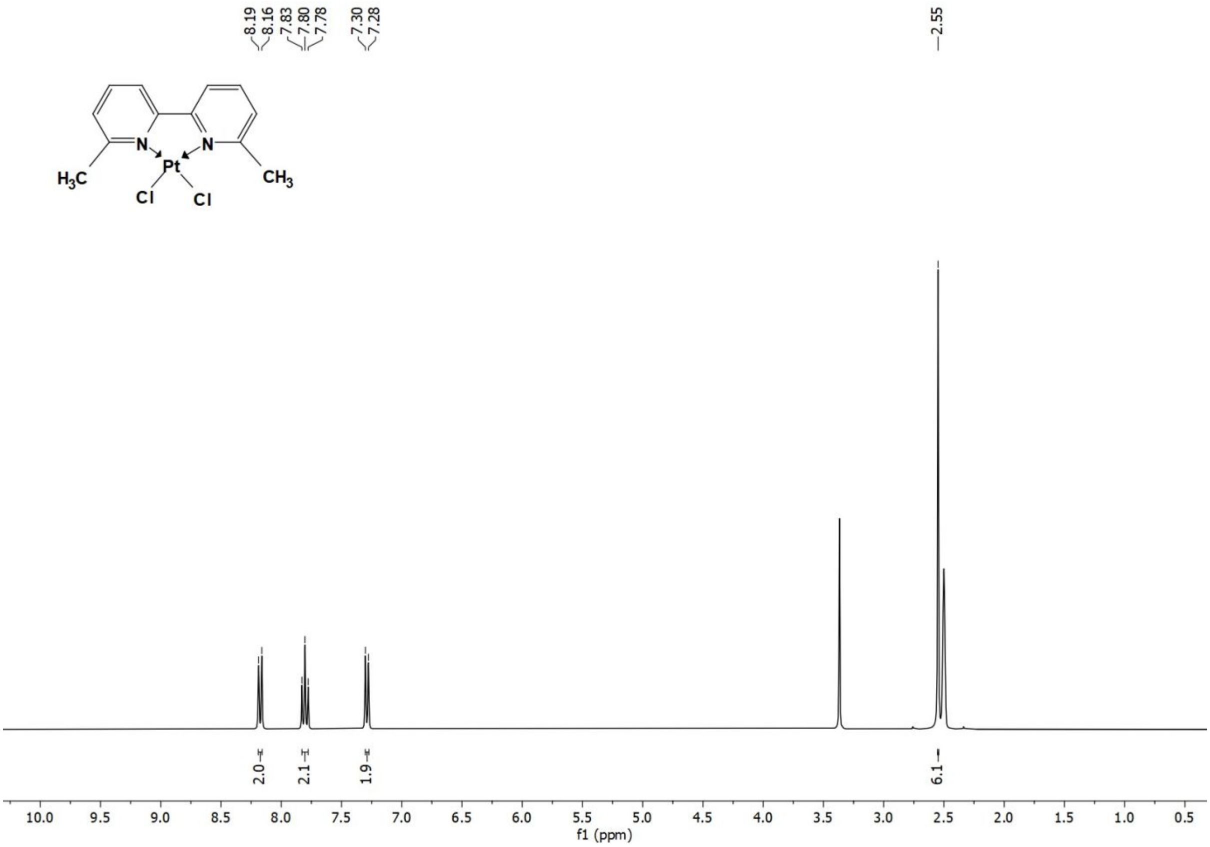

Figure S15. ^{1}H-NMR spectrum of compound **3l** in DMSO-d_6.

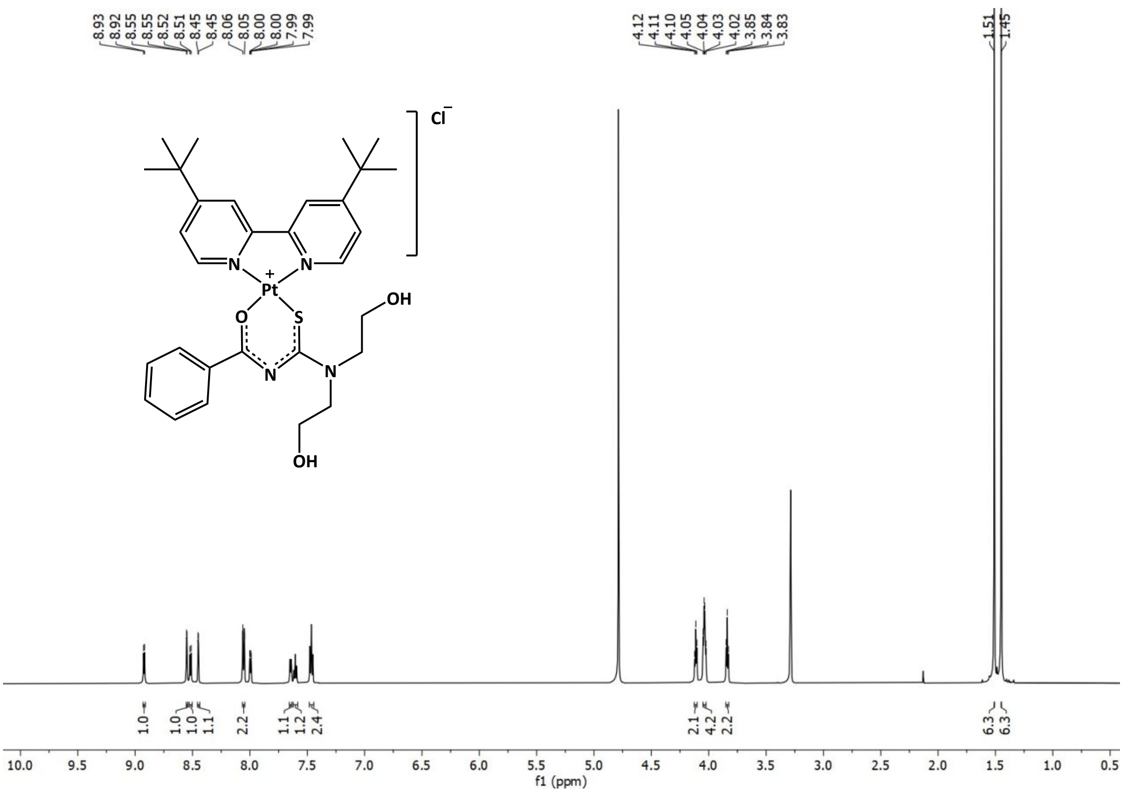

Figure S16. ^{1}H-NMR spectrum of compound **C1** in MeOD-d_4.

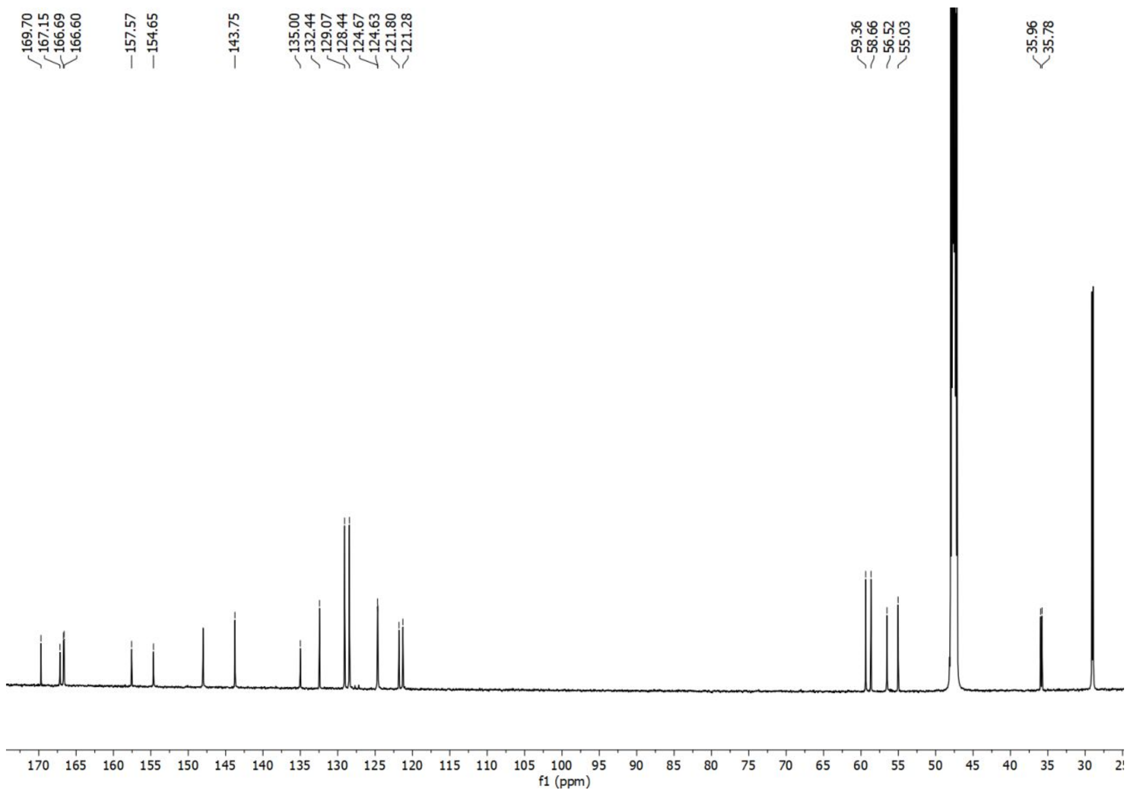

Figure S17.^{13}C-NMR spectrum of compound **C1** MeOD-d_4.

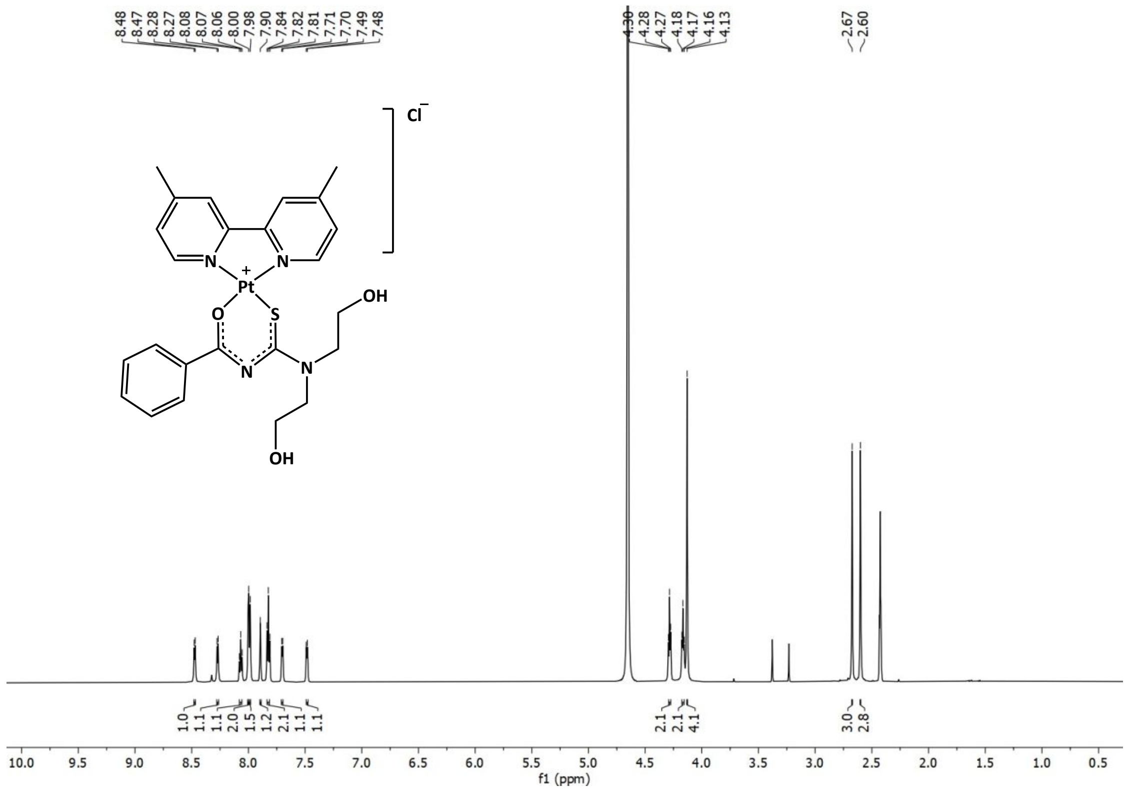

Figure S18. ^{1}H-NMR spectrum of compound **C2** in D_2O/CD_3CN-d_3.

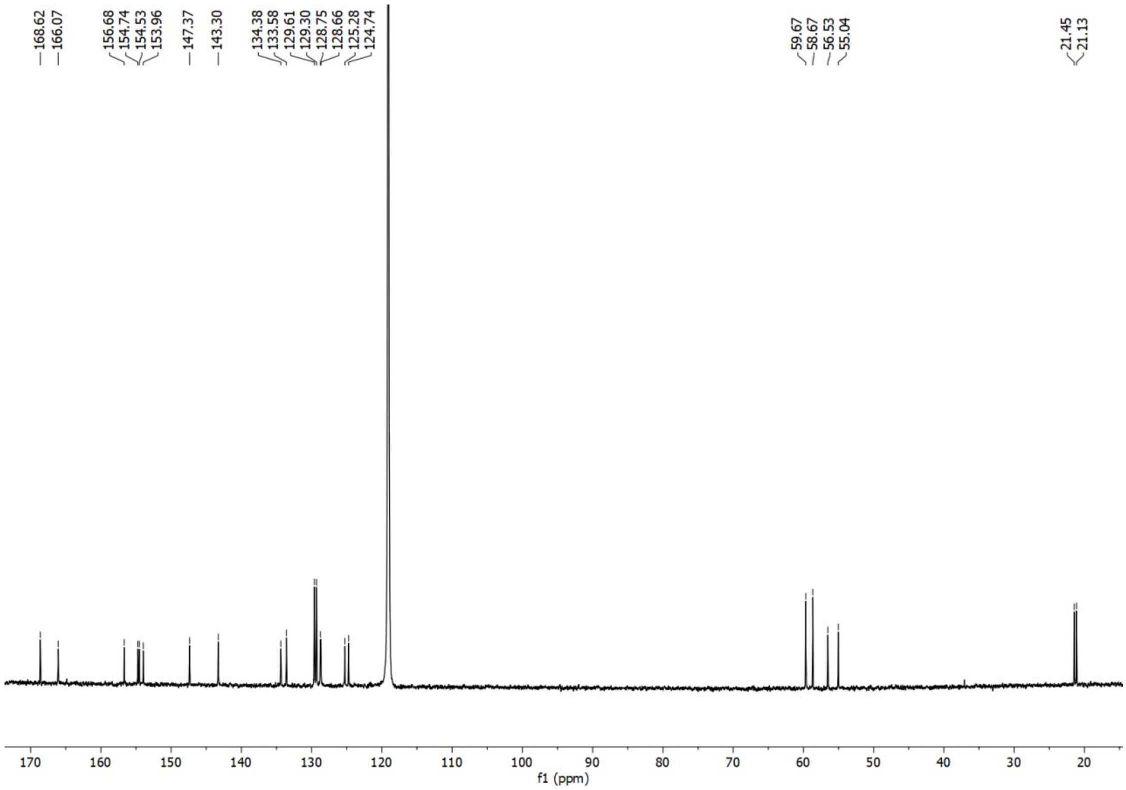

Figure S19.^{13}C-NMR spectrum of compound **C2** in D_2O.

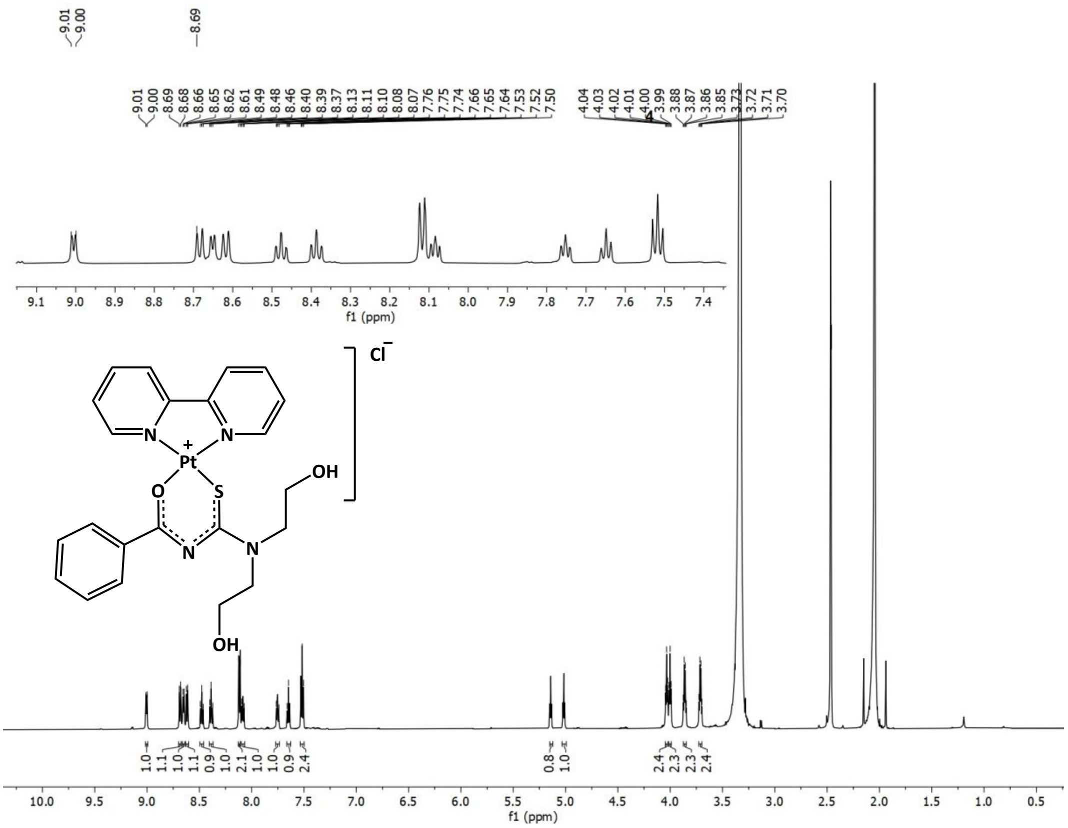

Figure S20. ^{1}H-NMR spectrum of compound **C3** in DMSO-d_6.

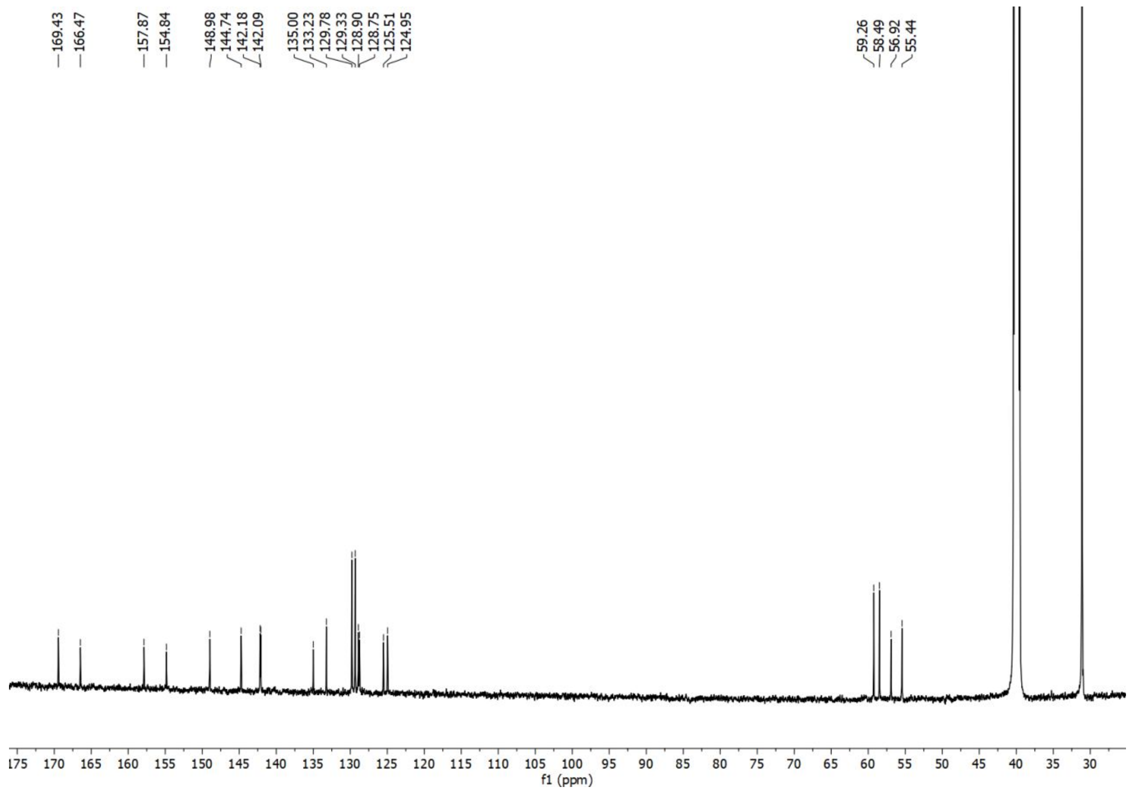

Figure S21. ^{13}C-NMR spectrum of compound **C3** in DMSO-d_6.

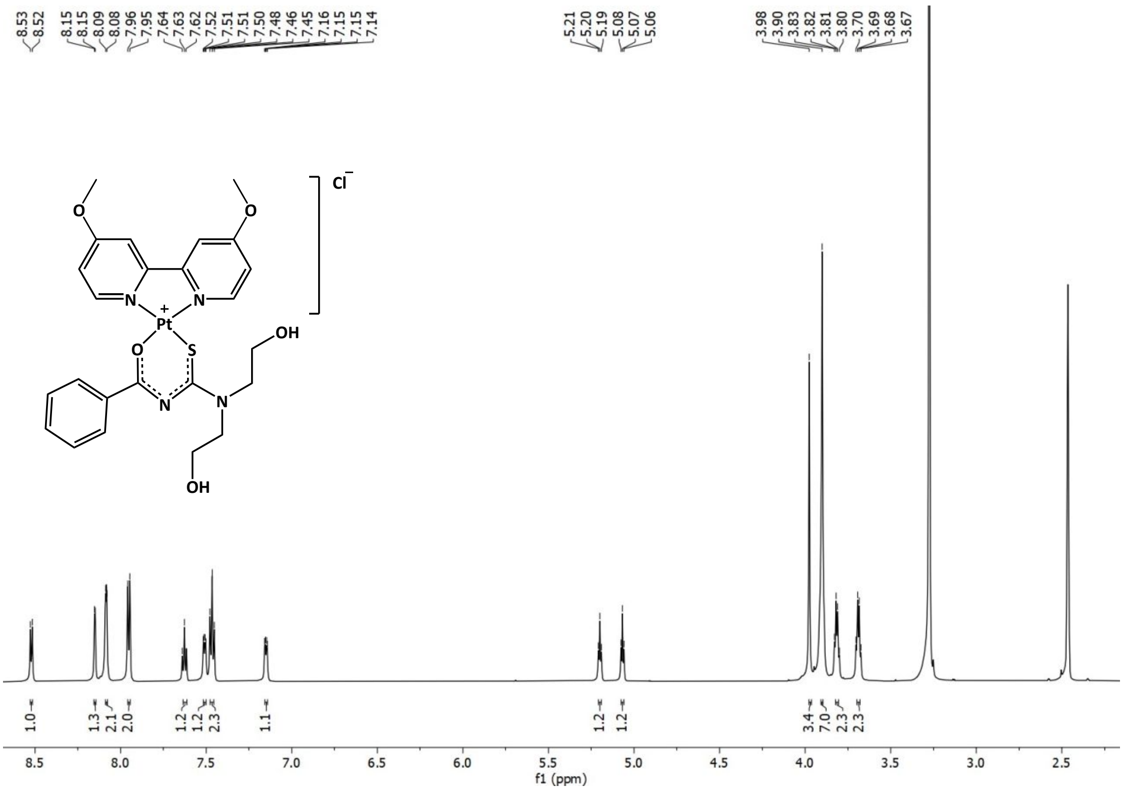

Figure S22. ^{1}H-NMR spectrum of compound **C4** in DMSO-d_6.

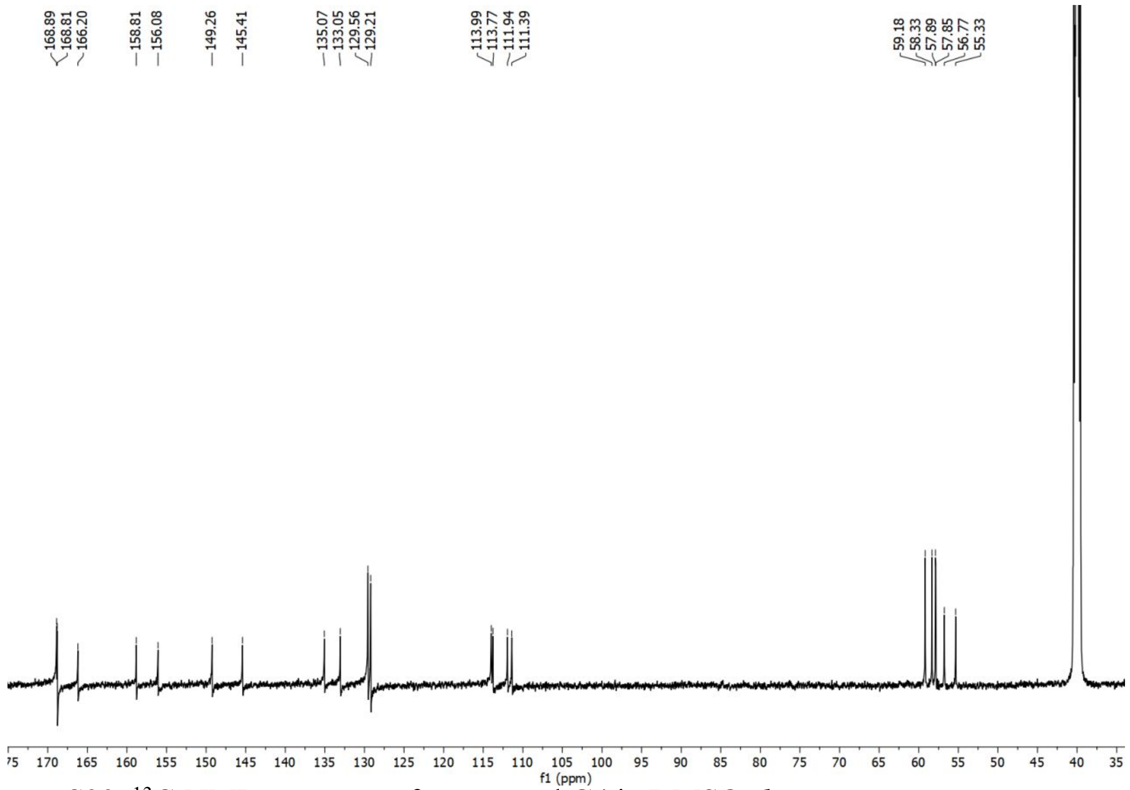

Figure S23. ^{13}C-NMR spectrum of compound **C4** in DMSO-d_6.

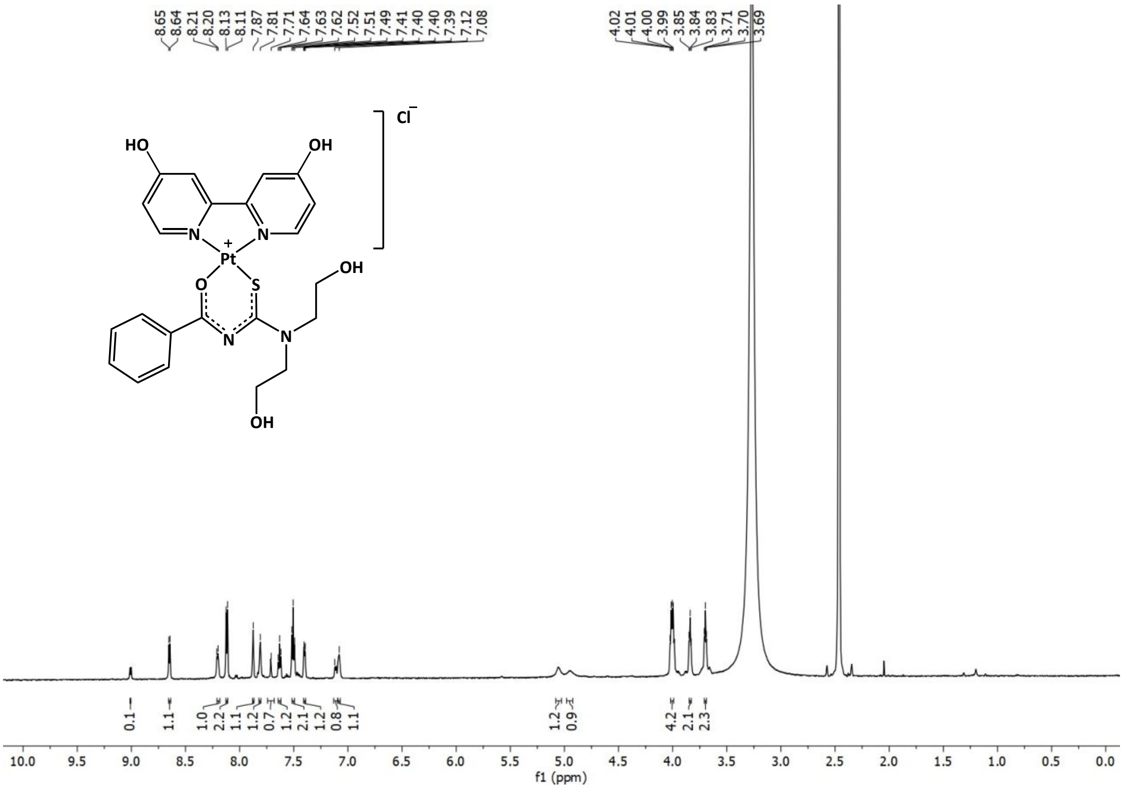

Figure S24. ^{1}H-NMR spectrum of compound **C5** in DMSO-d_6.

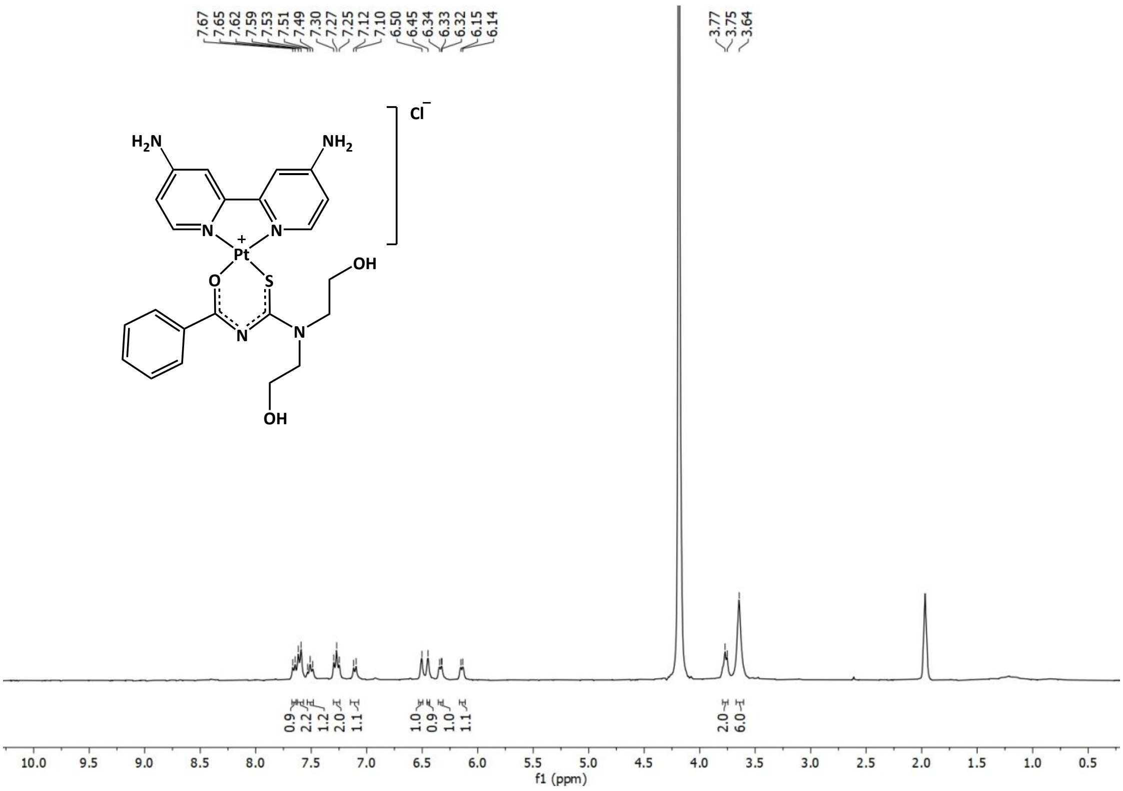

Figure S25. ^{1}H-NMR spectrum of compound **C6** in CD_3CN-*d_3*/D2O.

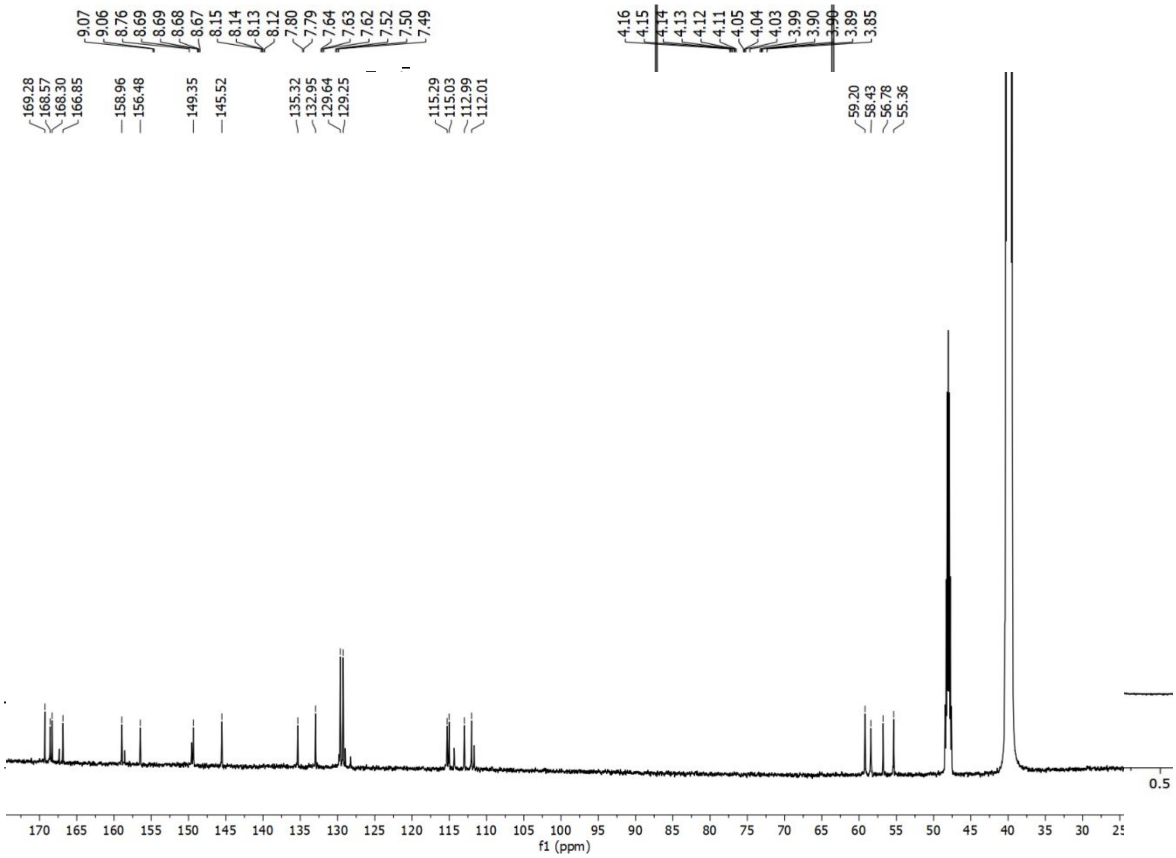

Figure S26. ^{1}H-NMR spectrum of compound **C7** in MeOD-*d_4*.

Figure S27. ^{13}C-NMR spectrum of compound **C7** in DMSO-d_6.

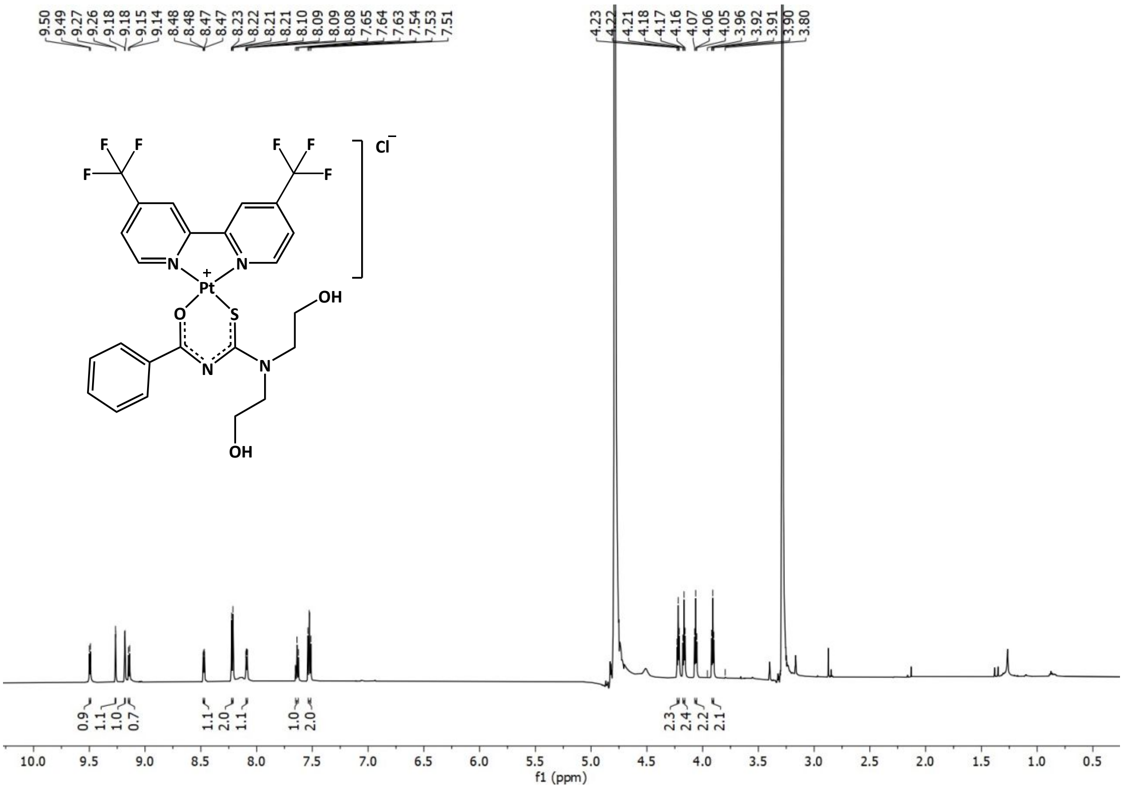

Figure S28. ^{1}H-NMR spectrum of compound **C8** in MeOD-d_4.

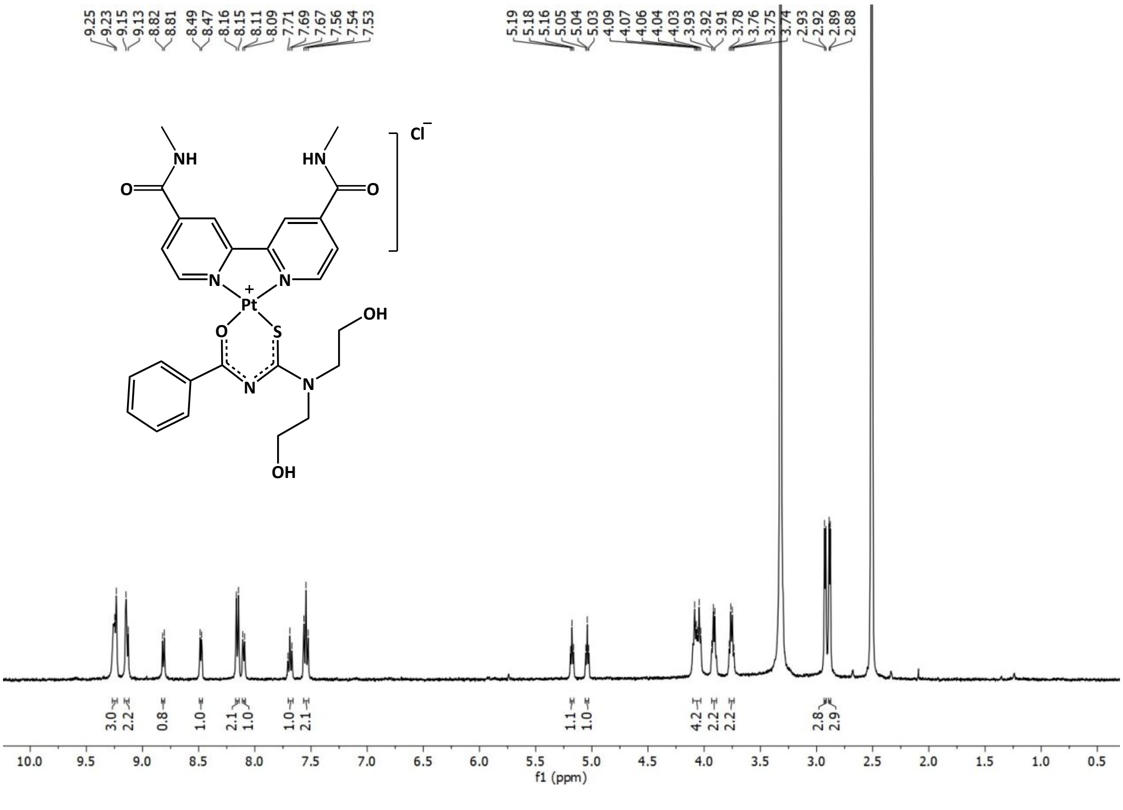

Figure S29. ^{1}H-NMR spectrum of compound **C9** in DMSO-d_6.

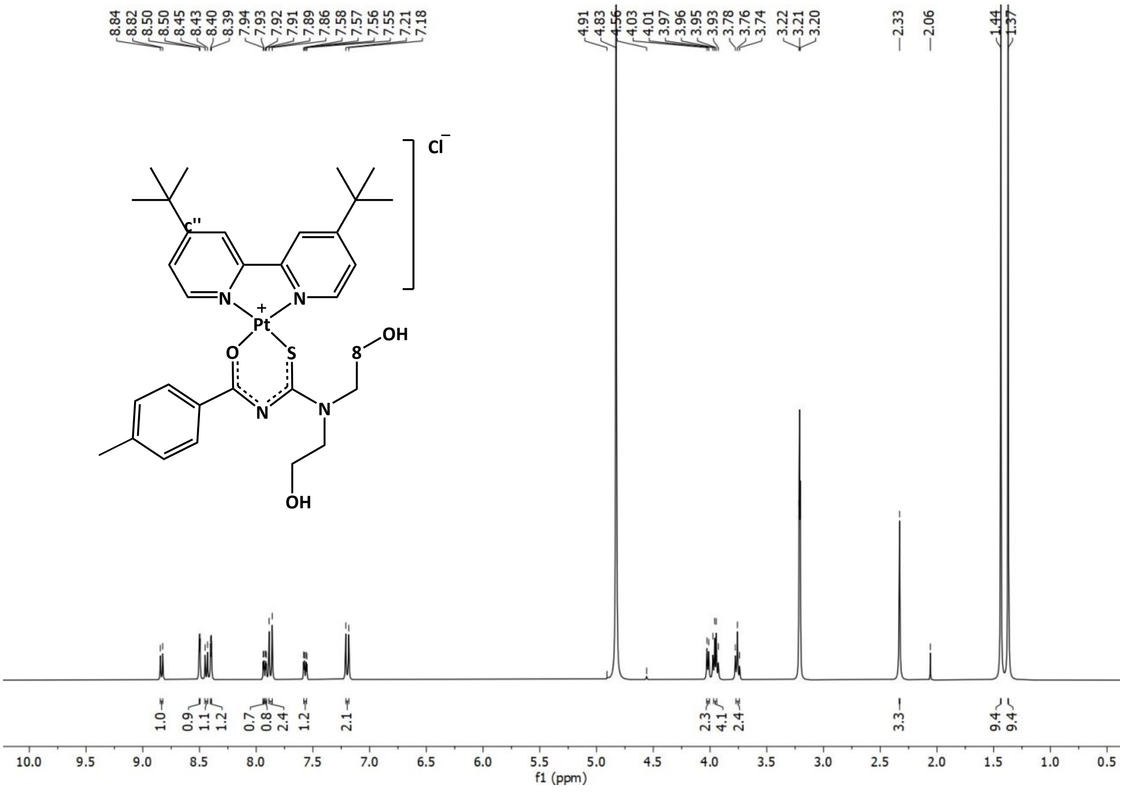

Figure S30. ^{1}H-NMR spectrum of compound **C11** in MeOD-d_4.

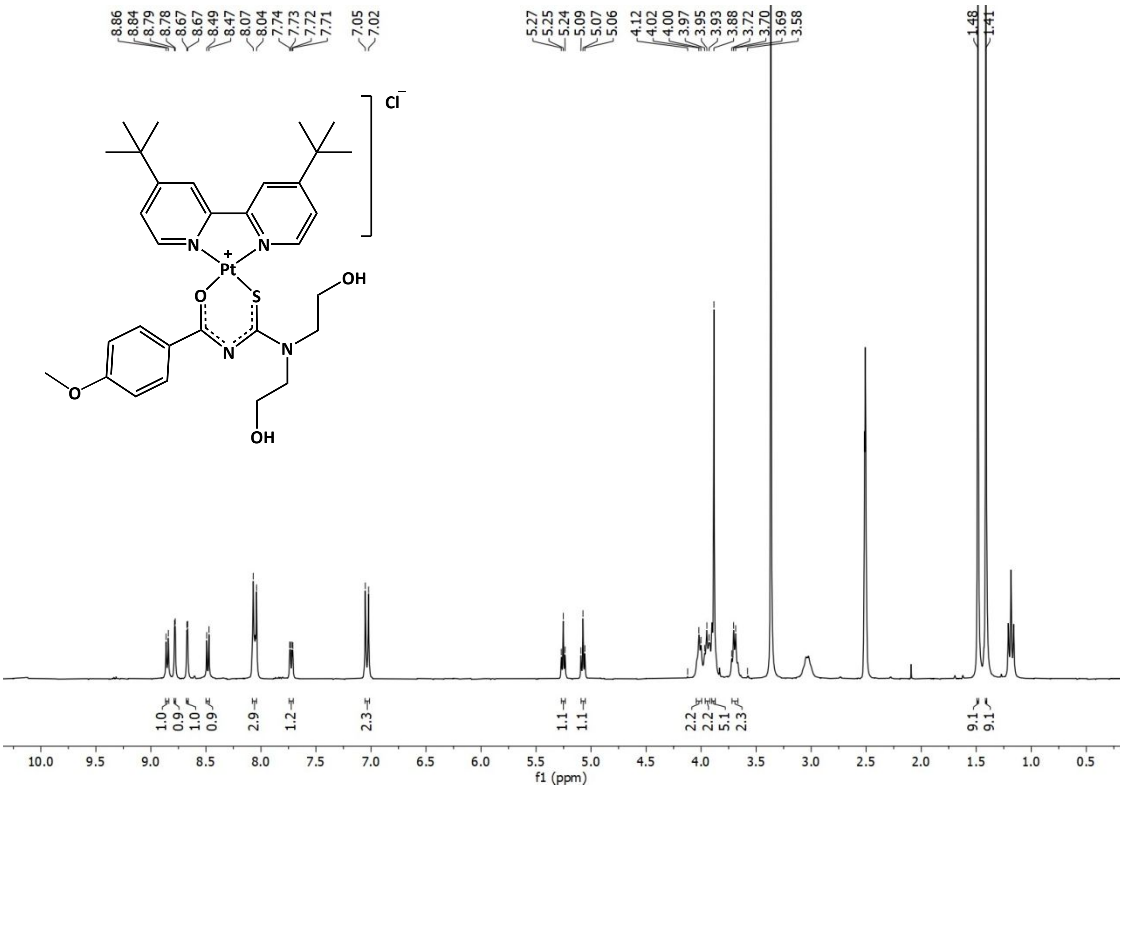

Figure S31. ^{1}H-NMR spectrum of compound **C12** in DMSO-d_6.

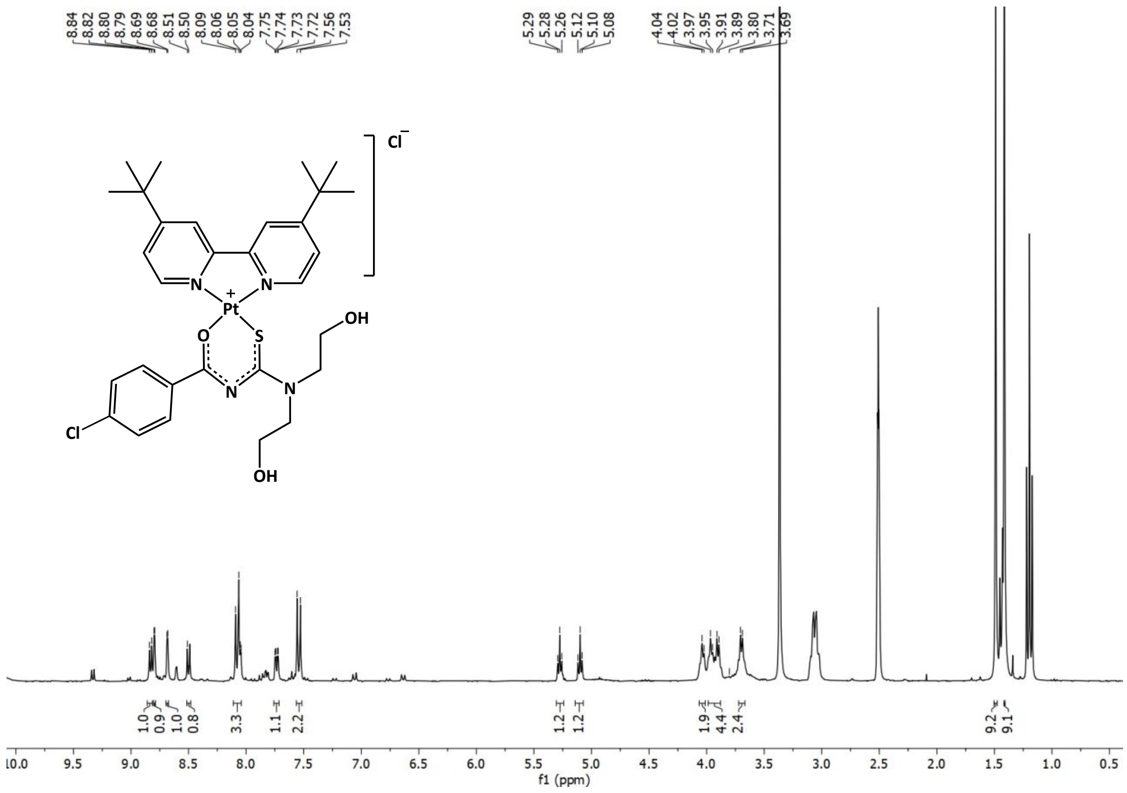

Figure S32. ^{1}H-NMR spectrum of compound **C13** in DMSO-d_6.

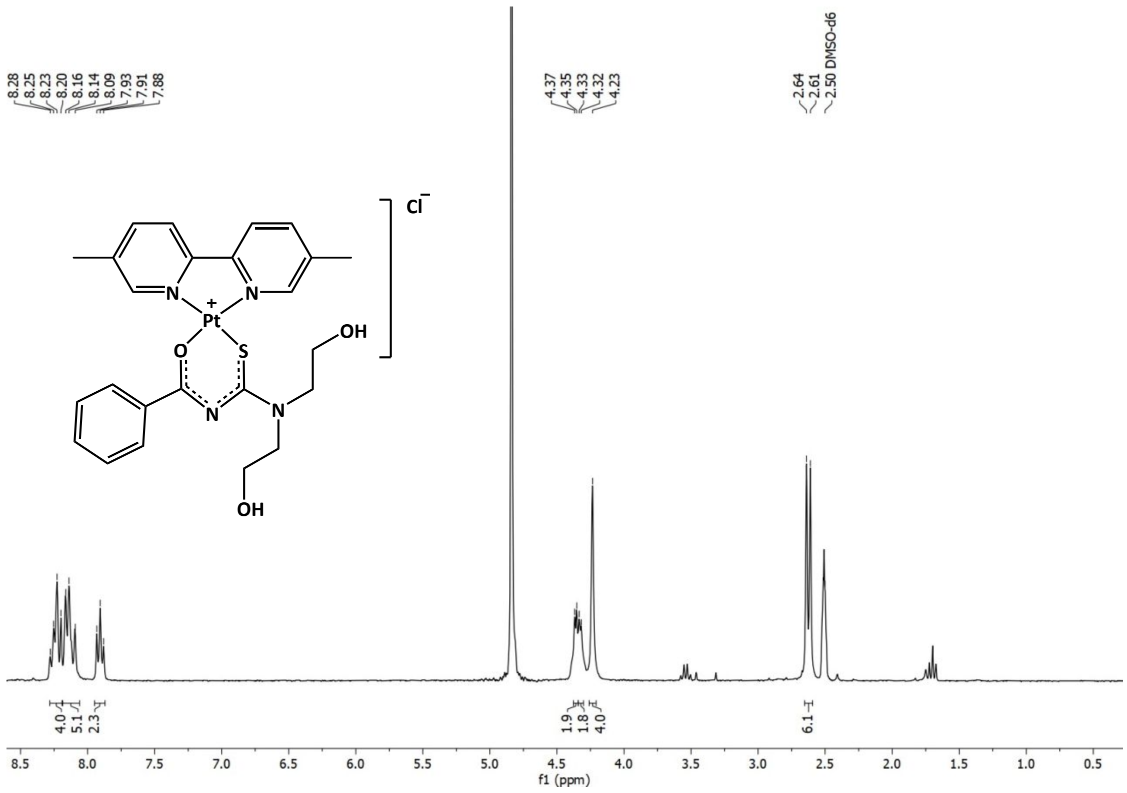

Figure S33. ^{1}H-NMR spectrum of compound **C14** in D_2O/CD_3CN-*d*$_3$.

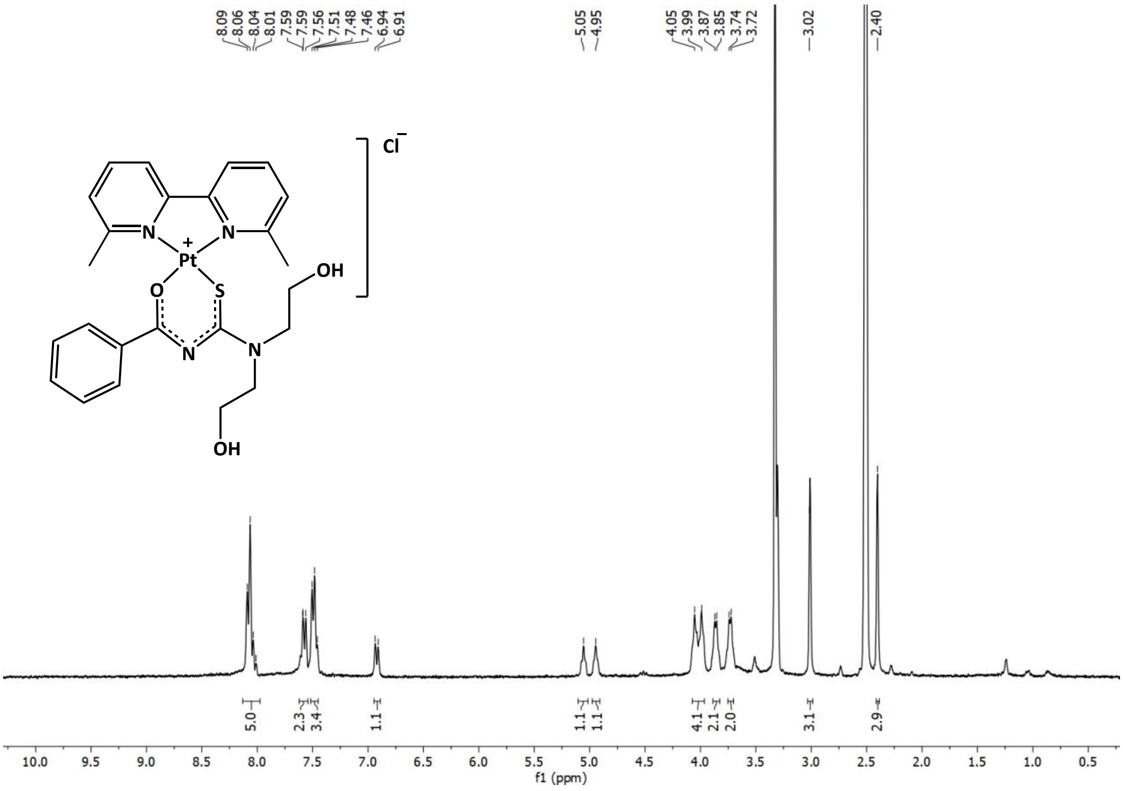

Figure S34. ^{1}H NMR spectrum of compound **C15** in DMSO-*d*$_6$.

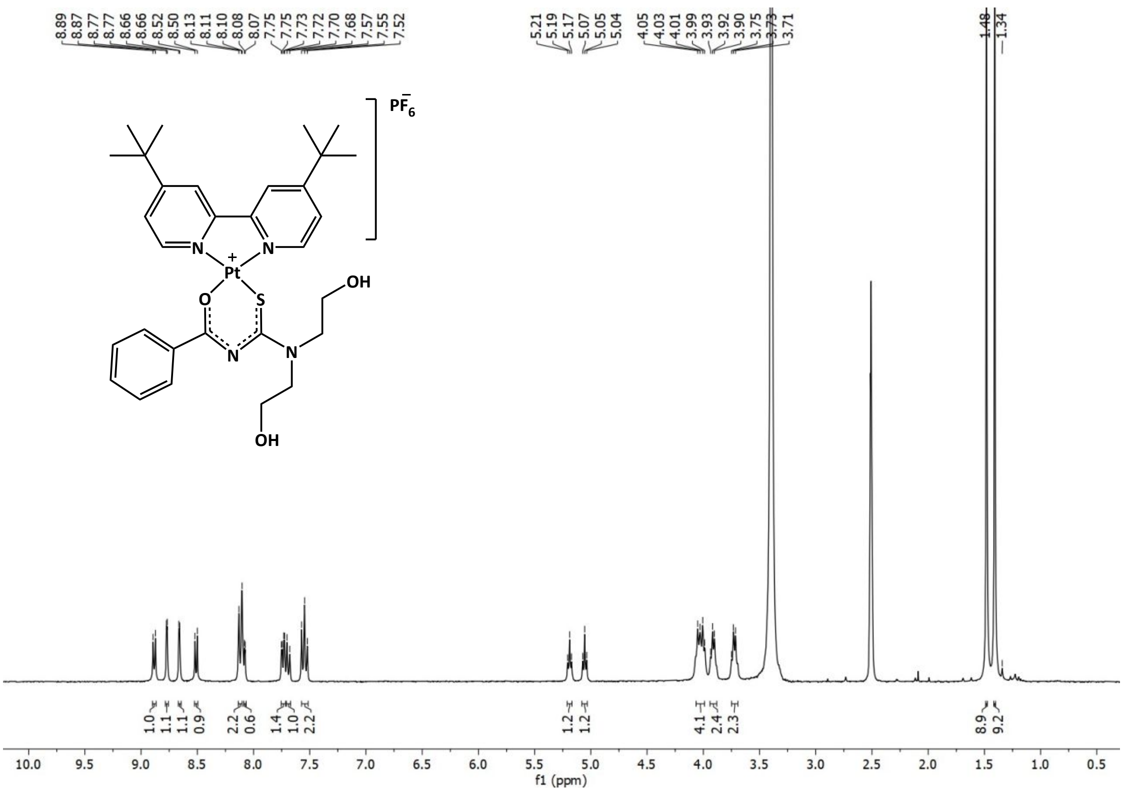

Figure S35. ^{1}H NMR spectrum of compound **C16** in DMSO-d_6.

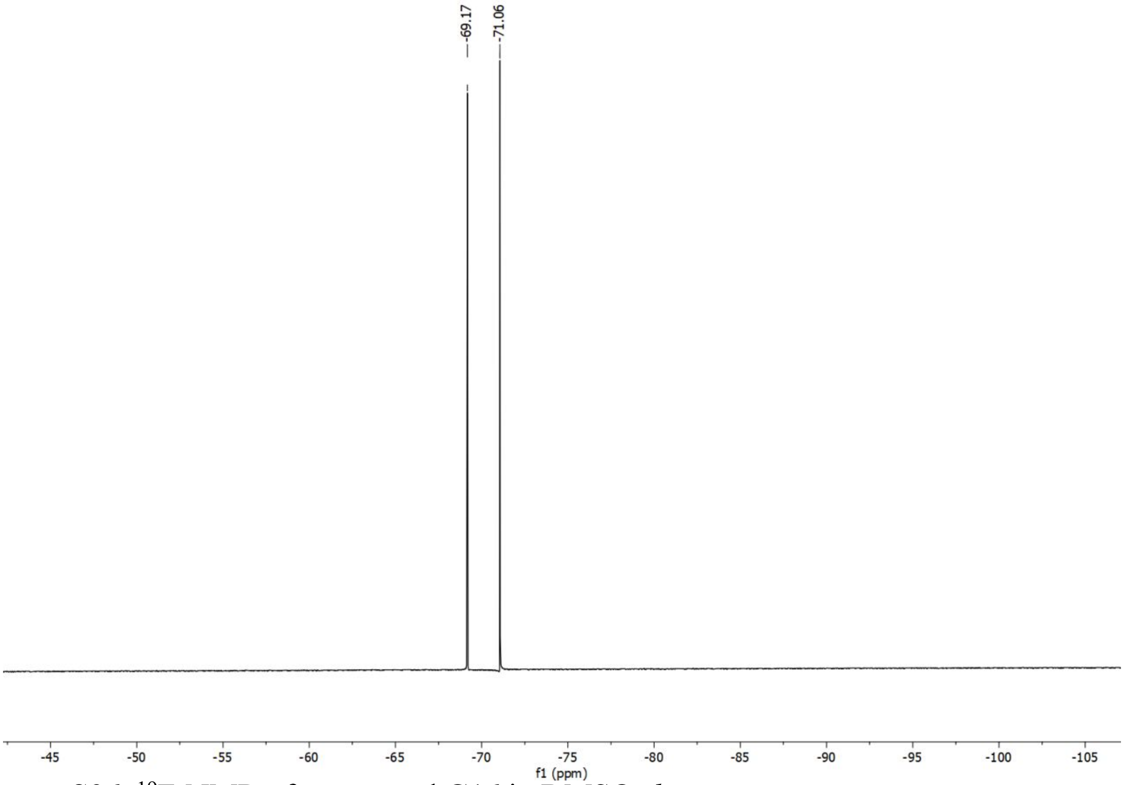

Figure S36. ^{19}F-NMR of compound **C16** in DMSO-d_6.

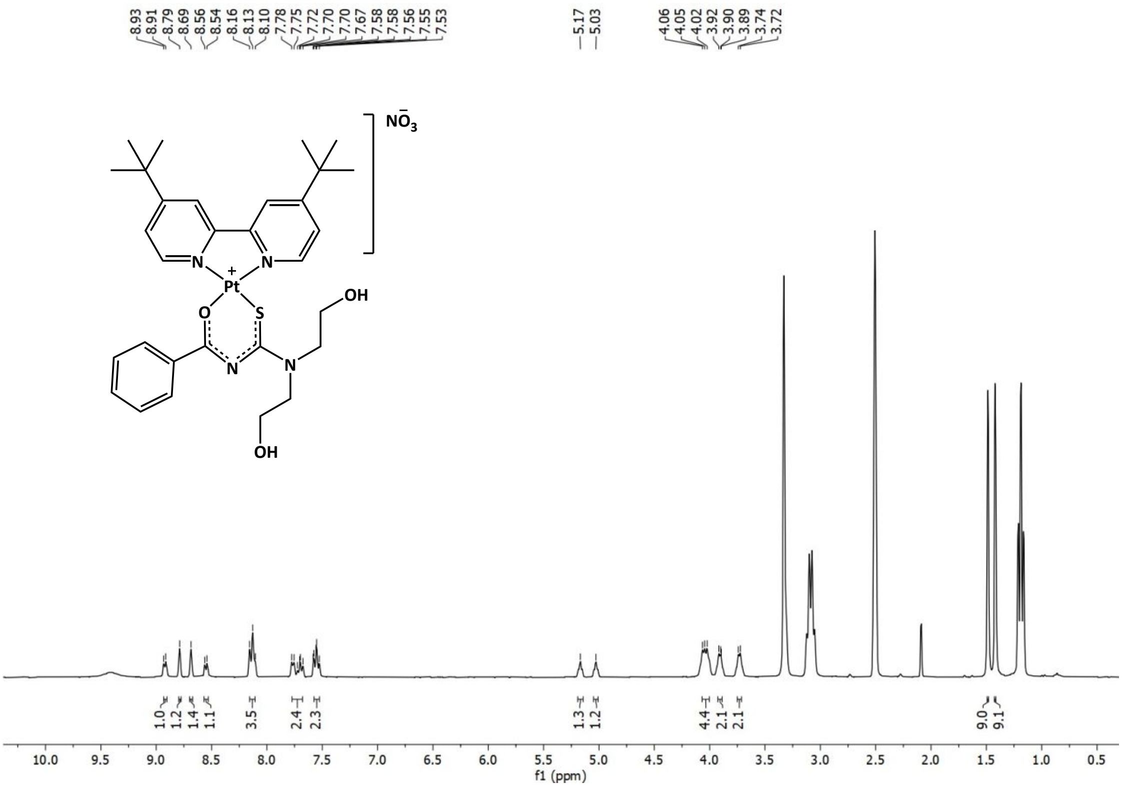

Figure S37. ^{1}H-NMR of compound **C17** in DMSO-d_6.

3. Cyclic voltammograms

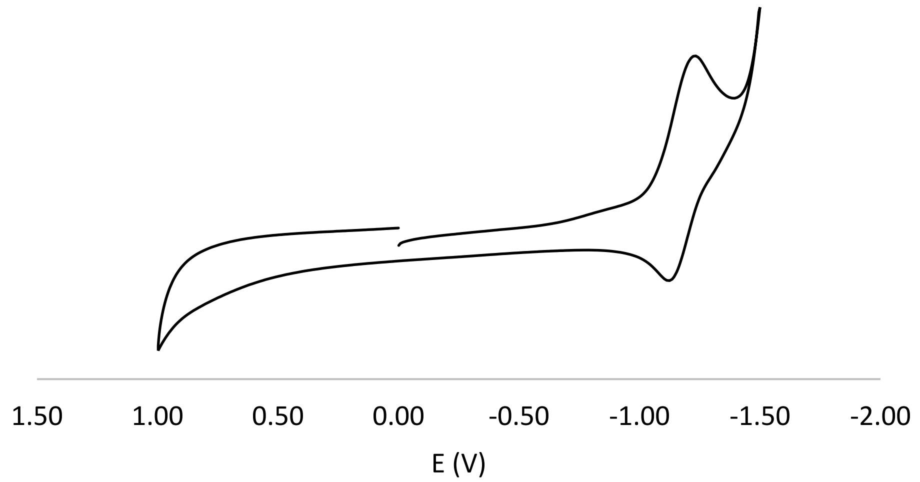

Figure S38. Cyclic voltammogram of compound **C1**

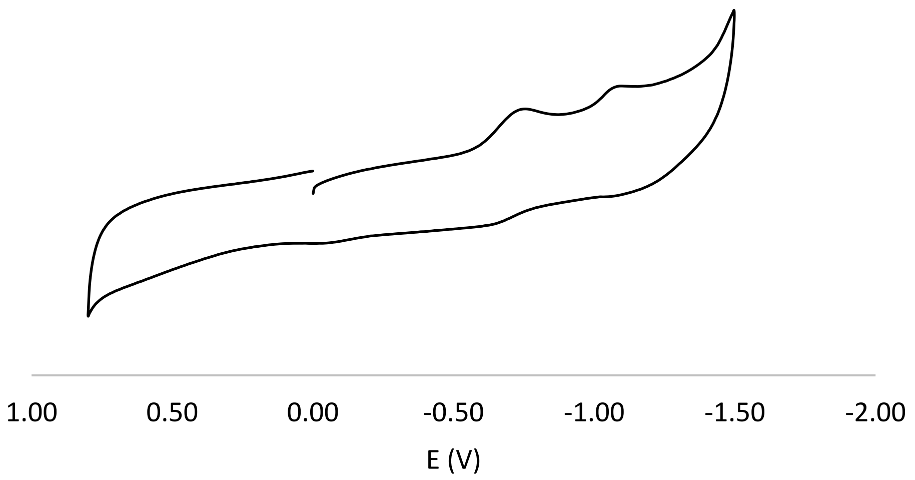

Figure S39. Cyclic voltammogram of compound **C2**

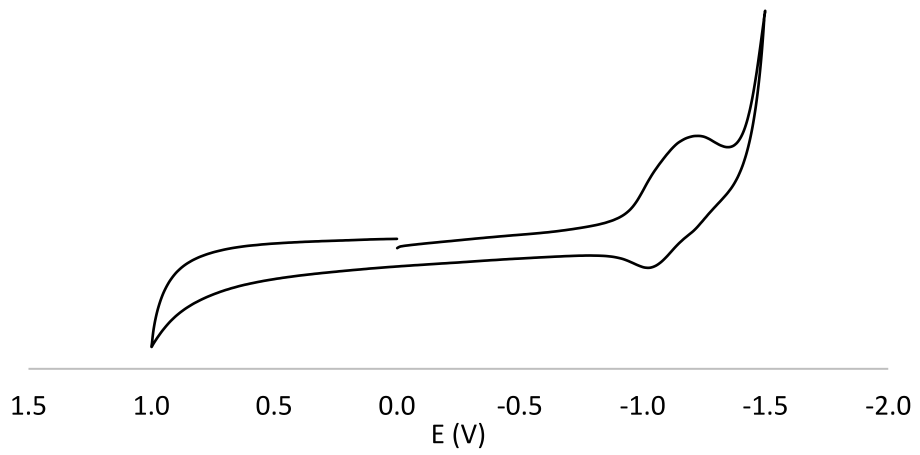

Figure S40. Cyclic voltammogram of compound **C3**

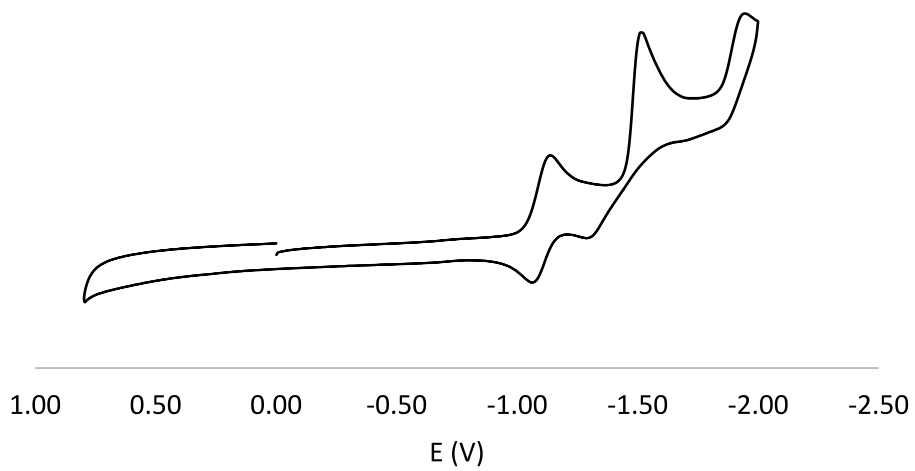

Figure S41. Cyclic voltammogram of compound **C4**

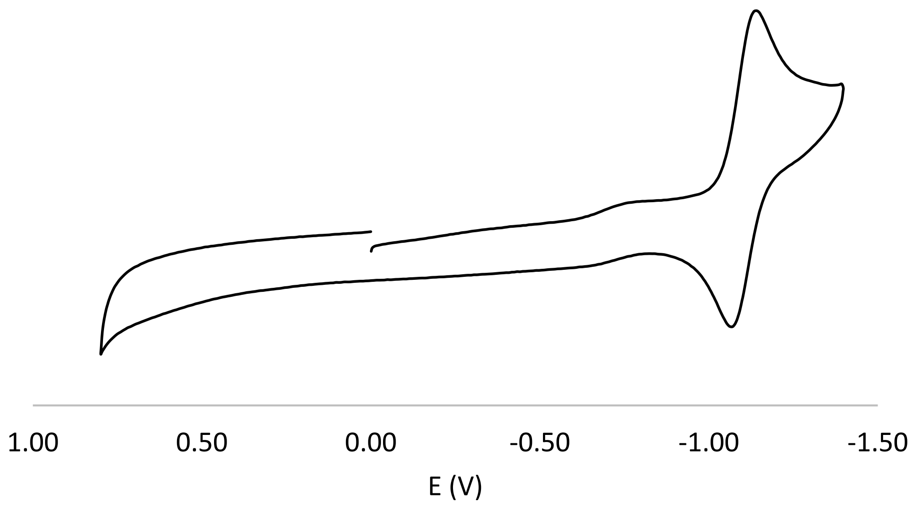

Figure S42. Cyclic voltammogram of compound **C6**

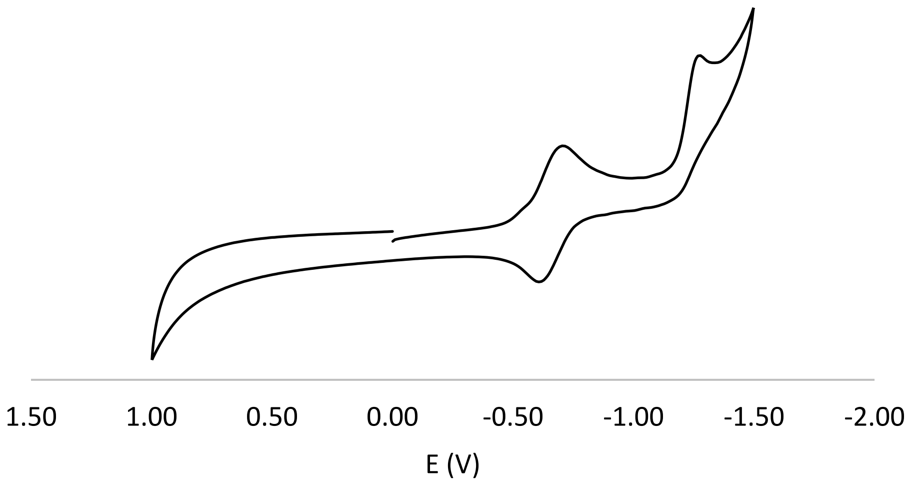

Figure S43. Cyclic voltammogram of compound **C8**

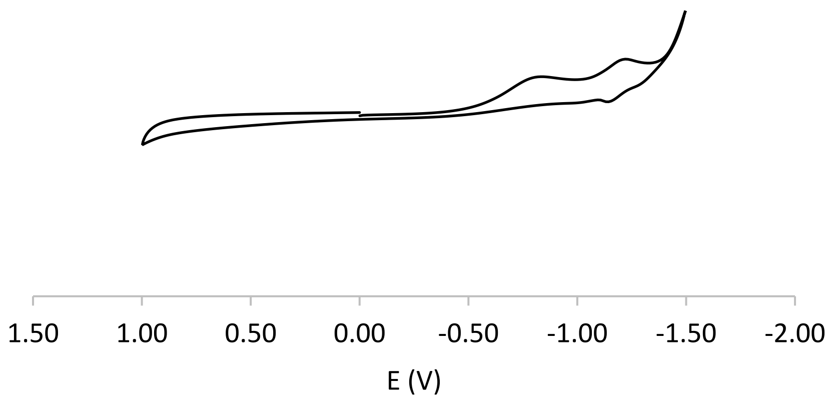

Figure S44. Cyclic voltammogram of compound **C11**

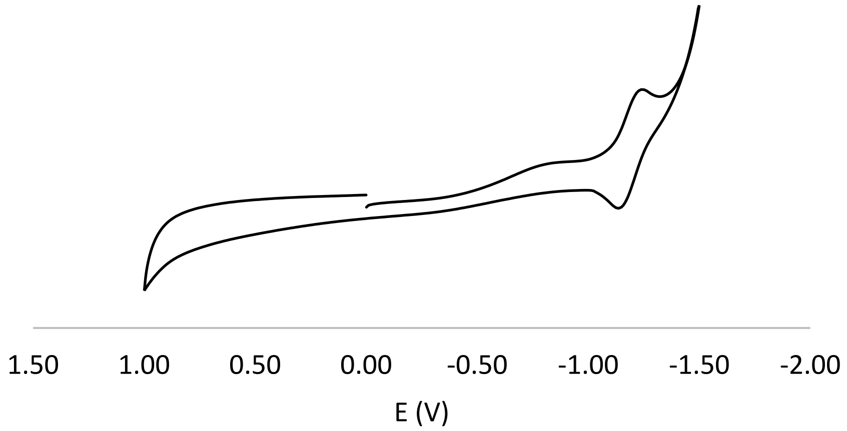

Figure S45. Cyclic voltammogram of compound **C12**

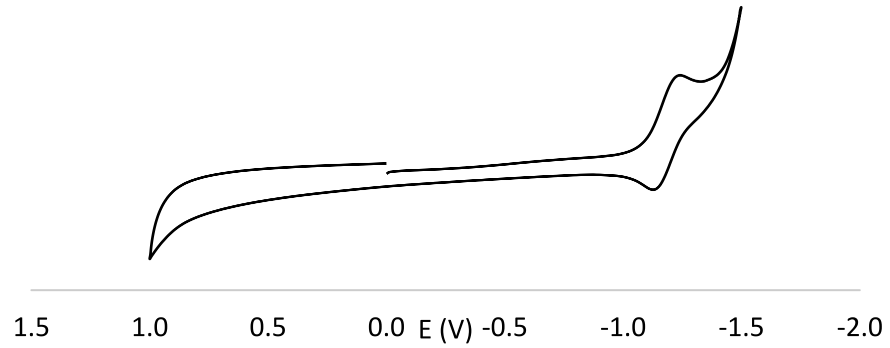

Figure S46. Cyclic voltammogram of compound **C13**

4. Glutathione binding and hemolysis studies

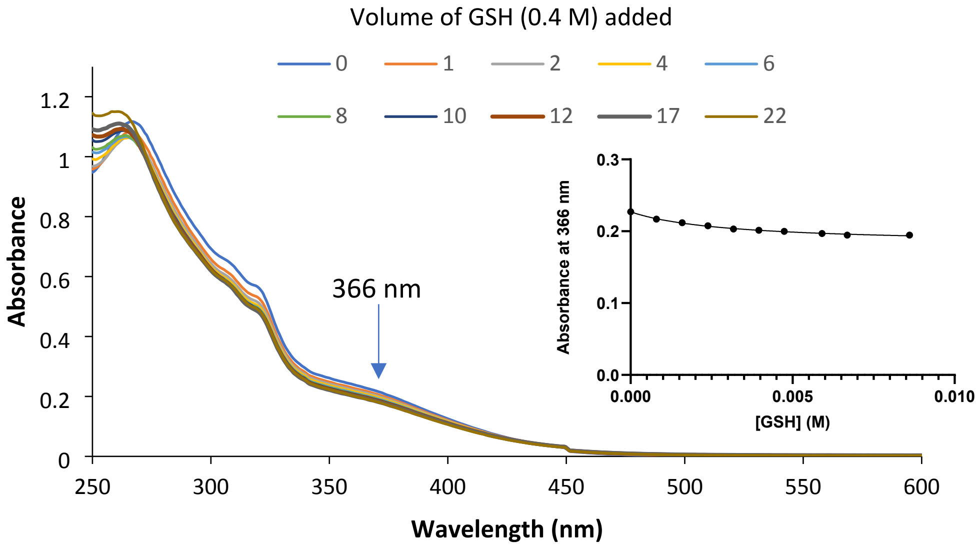

Figure S47. Representative spectrum showing changes in the UV-visible absorption spectrum of **C1** with increasing amounts of GSH (0.4 M) in PBS (pH 7.2) at 25 °C. Right side panel shows the decrease in absorbance at 366 nm with increasing concentrations of GSH. The UV-Vis spectra were corrected for dilution.

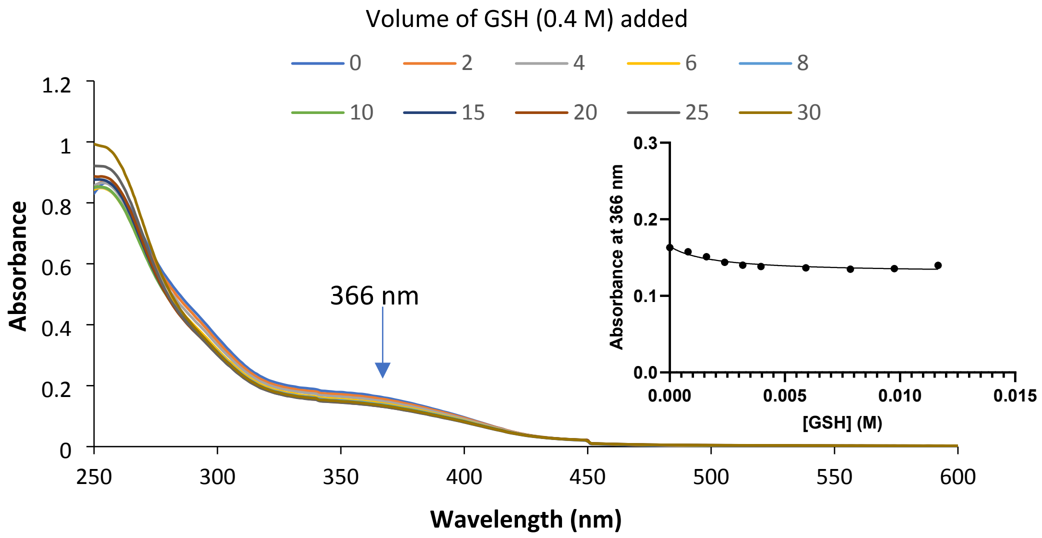

Figure S48. Representative spectrum showing changes in the UV-visible absorption spectrum of **C6** with increasing amounts of GSH (0.4 M) in PBS (pH 7.2) at 25 °C. Right side panel shows the decrease in absorbance at 366 nm with increasing concentrations of GSH. The UV-Vis spectra were corrected for dilution.

Table S1. Log*K* values for the binding of GSH to platinum(II) complexes. Experiments were conducted in triplicate (*N* = 3) with the mean and standard error of the mean (SEM) reported.

**Compound**	**LogK**			**Average logK ± SEM**
	***N* = 1**	***N* = 2**	***N* = 3**	
**C1**	2.48	2.53	2.33	2.45 ± 0.05
**C6**	2.80	2.21	2.74	2.58 ± 0.19

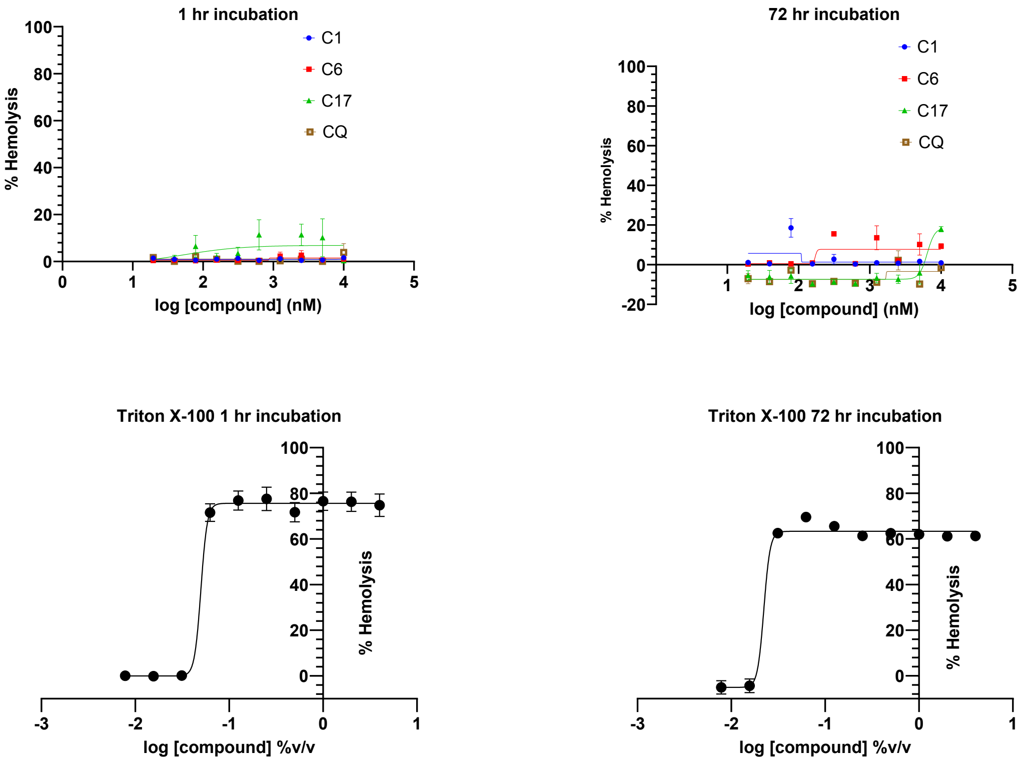

Figure S49. Graphs showing the percentage hemolysis induced in RBCs by compounds **C1**, **C6** and **C17** after 1 hour and 72 hours of incubation. The percentage hemolysis induced by chloroquine (negative control) and Triton X-100 (positive control) are also shown.

Table S2: Dual point activity of platinum(II) complexes against ABS parasites, early- and late-stage gametocytes. Values are averaged from technical triplicates performed for a single biological repeat.

	**ABS (% inhibition)**		**EG (% inhibition)**		**LG (% inhibition)**	
	**1 µM**	**5 µM**	**1 µM**	**5 µM**	**1 µM**	**5 µM**
**FA-4<br>C1**	91.4	105.2	7.0	86.1	45.2	86.0
**FA-27<br>C2**	70.9	104.0	19.9	7.9	11.3	25.5
**FA-24<br>C3**	68.7	103.1	26.9	0.0	14.8	39.8
**FA-31<br>C4**	67.1	99.8	6.8	9.1	14.8	54.4
**FA-56<br>C5**	0.0	11.0	0.0	0.0	0.0	0.5
**FA-86<br>C6**	100.7	100.2	13.7	20.1	42.3	32.4
**FA-54<br>C7**	2.7	57.3	2.2	4.1	0.0	0.4
**FA-76<br>C8**	10.6	29.9	26.2	50.0	80.6	85.8
**FA-53<br>C9**	0.0	24.5	0.0	4.5	0.0	2.4

**FA-79 C10**	7.11	17.3	3.7	4.2	0.6	6.1
**FA-101 C11**	98.7	99.9	ND	ND	ND	ND
**FA-99 C12**	92.5	99.7	ND	ND	ND	ND
**FA-97 C13**	86.3	99.2	ND	ND	ND	ND
**FA-133 C14**	ND	ND	0.0	0.0	39.4	56.6
**FA-167 C15**	0.0	89.6	2.8	43.9	54.8	84.0
**FA-154 C16**	ND	ND	0.0	49.5	0.0	29.2
**FA-178 C17**	99.5	109.3	17.3	89.7	31.3	94.7

5. References

(1) Leshabane, M.; Dziwornu, G. A.; Coertzen, D.; Reader, J.; Moyo, P.; Van Der Watt, M.; Chisanga, K.; Nsanzubuhoro, C.; Ferger, R.; Erlank, E.; Venter, N.; Koekemoer, L.; Chibale, K.; Birkholtz, L. M. Benzimidazole Derivatives Are Potent against Multiple Life Cycle Stages of Plasmodium Falciparum Malaria Parasites. *ACS Infect Dis* **2021**, *7* (7), 1945–1955. https://doi.org/10.1021/acsinfecdis.0c00910.

(2) Reader, J.; Botha, M.; Theron, A.; Lauterbach, S. B.; Rossouw, C.; Engelbrecht, D.; Wepener, M.; Smit, A.; Leroy, D.; Mancama, D.; Coetzer, T. L.; Birkholtz, L. M. Nowhere to Hide: Interrogating Different Metabolic Parameters of Plasmodium Falciparum Gametocytes in a Transmission Blocking Drug Discovery Pipeline towards Malaria Elimination. *Malar J* **2015**, *14* (1). https://doi.org/10.1186/s12936-015-0718-z.

(3) Reader, J.; van der Watt, M. E.; Birkholtz, L.-M. Streamlined and Robust Stage-Specific Profiling of Gametocytocidal Compounds Against Plasmodium Falciparum. *Front Cell Infect Microbiol* **2022**, *12*, 926460. https://doi.org/10.3389/fcimb.2022.926460.

(4) Di, L.; Kerns, E. *Drug-like Properties: Concepts, Structure Design and Methods: From ADME to Toxicity Optimization*, 1st ed.; Academic Press (Elsevier Inc), 2008.

(5) Zhou, L.; Yang, L.; Tilton, S.; Wang, J. Development of a High Throughput Equilibrium Solubility Assay Using Miniaturized Shake-Flask Method in Early Drug Discovery. *J Pharm Sci* **2007**, *96* (11), 3052–3071. https://doi.org/10.1002/jps.20913.

(6) Di, L.; Kerns, E. H.; Gao, N.; Li, S. Q.; Huang, Y.; Bourassa, J. L.; Huryn, D. M. Experimental Design on Single-Time-Point High-Throughput Microsomal Stability Assay. *J Pharm Sci* **2004**, *93* (6), 1537–1544. https://doi.org/10.1002/jps.20076.

(7) Obach, R. S. Prediction of Human Clearance of Twenty-Nine Drugs from Hepatic Microsomal Intrinsic Clearance Data: An Examination of in Vitro Half-Life Approach and Nonspecific Binding to Microsomes. *Drug Metab Dispos* **1999**, *27* (11), 1350–1359.

(8) Kanyora, A. K.; Omondi, R. O.; Ongoma, P.; Omolo, J. O.; Welsh, A.; Prince, S.; Gichumbi, J.; Mambanda, A.; Smith, G. S. Mononuclear H6-Arene Ruthenium(II) Complexes with Pyrazolyl–Pyridazine Ligands: Synthesis, CT-DNA Binding, Reactivity towards Glutathione, and Cytotoxicity. *JBIC Journal of Biological Inorganic Chemistry* **2024**, *29* (2), 251–264. https://doi.org/10.1007/s00775-024-02043-3.

(9) Egan, T. J.; Mavuso, W. W.; Ross, D. C.; Marques, H. M. Thermodynamic Factors Controlling the Interaction of Quinoline Antimalarial Drugs with Ferriprotoporphyrin IX. *J Inorg Biochem* **1997**, *68* (2), 137–145. https://doi.org/10.1016/s0162-0134(97)00086-x.
